# ACMG/AMP interpretation of *BRCA1* missense variants: Structure-informed scores add evidence strength granularity to the PP3/BP4 computational evidence

**DOI:** 10.1016/j.ajhg.2024.12.011

**Published:** 2025-04-14

**Authors:** Lobna Ramadane-Morchadi, Nitsan Rotenberg, Ada Esteban-Sánchez, Cristina Fortuno, Alicia Gómez-Sanz, Matthew J. Varga, Adam Chamberlin, Marcy E. Richardson, Kyriaki Michailidou, Pedro Pérez-Segura, Amanda B. Spurdle, Miguel de la Hoya

**Affiliations:** 1 Molecular Oncology Laboratory, Hospital Clínico San Carlos, IdISSC (Instituto de Investigación Sanitaria del Hospital Clínico San Carlos), 28040 Madrid, Spain; 2 University of Queensland, Brisbane, QLD, Australia; 3 Molecular Cancer Epidemiology Laboratory, QIMR Berghofer MRI, Herston, QLD 4006, Australia; 4 Ambry Genetics, Aliso Viejo, CA 92656, USA; 5 Biostatistics Unit, The Cyprus Institute of Neurology & Genetics, 2371 Nicosia, Cyprus

**Keywords:** ACMG/AMP, PP3/BP4, BRCA1, AlphaFold, AlphaMissense, ΔΔG, RSA, BayesDel, MAVE

## Abstract

Classification of missense variants is challenging. Lacking compelling clinical and/or functional data, ACMG/AMP lines of evidence are restricted to PM2 (rarity code applied at supporting level) and PP3/BP4 (computational evidence based mostly on multiple-sequence-alignment conservation tools). Currently, the ClinGen ENIGMA *BRCA1*/*2* Variant Curation Expert Panel uses BayesDel to apply PP3/BP4 to missense variants located in the BRCA1 RING/BRCT domains. The ACMG/AMP framework does not refer explicitly to protein structure as a putative source of pathogenic/benign evidence. Here, we tested the value of incorporating structure-based evidence such as relative solvent accessibility (RSA), folding stability (ΔΔG), and/or AlphaMissense pathogenicity to the classification of *BRCA1* missense variants. We used MAVE functional scores as proxies for pathogenicity/benignity. We computed RSA and FoldX5.0 ΔΔG predictions using as alternative input templates for either PDB files or AlphaFold2 models, and we retrieved pre-computed AlphaMissense and BayesDel scores. We calculated likelihood ratios toward pathogenicity/benignity provided by the tools (individually or combined). We performed a clinical validation of major findings using the large-scale BRIDGES case-control dataset. AlphaMissense outperforms ΔΔG and BayesDel, providing similar PP3/BP4 evidence strengths with lower rate of variants in the uninformative score range. AlphaMissense combined with ΔΔG increases evidence strength granularity. AlphaFold2 models perform well as input templates for ΔΔG predictions. Regardless of the tool, BP4 (but not PP3) is highly dependent on RSA, with benignity evidence provided only to variants targeting buried or partially buried residues (RSA ≤ 60%). Stratification by functional domain did not reveal major differences. In brief, structure-based analysis improves PP3/BP4 assessment, uncovering a relevant role for RSA.

## Graphical abstract

**Figure:**
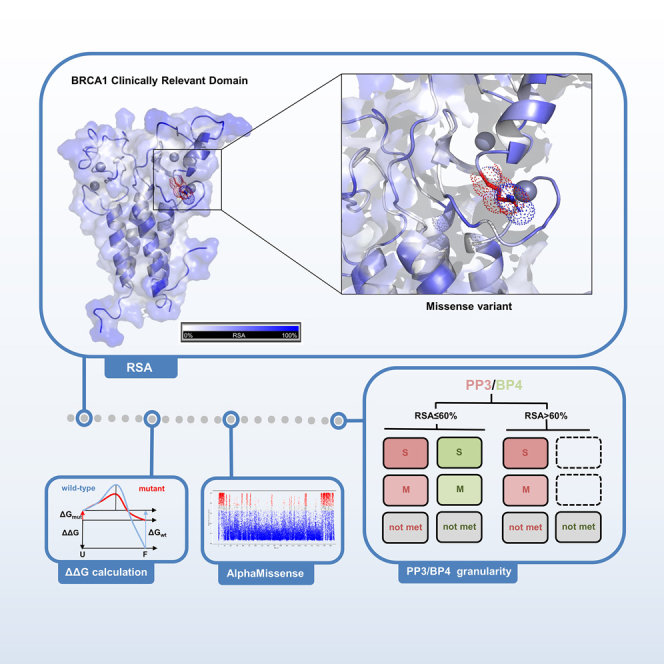

## Teaser

Specifications for the BRCA1 ACMG/AMP classification system recommend supporting strength for application of PP3/BP4 computational evidence. Focusing on missense variants targeting BRCA1 clinically relevant domains, we show that a structure-informed approach combining RSA with AlphaMissense and ΔΔG provides evidence strength granularity, with PP3/BP4 moderate or strong strength met by certain variants.

## Introduction

The introduction of next-generation sequencing in the clinical setting has revolutionized genetic diagnostics. However, the identification of an ever-growing number of genetic variants of uncertain significance (VUSs) presents a major challenge in the clinical interpretation of the findings. For genes where loss-of-function is the gene/disease association mechanism, most nonsense/frameshift variants are readily annotated as pathogenic, but assessment of missense changes is far more complex.

Mounting evidence suggests that a high proportion of missense changes have little or no effect on protein function, albeit some are severely damaging^1^ and, depending on the specific gene, the ratio of tolerated/damaging changes is very variable.^2^ Several studies have shown that reduced thermodynamic stability is a major driver of pathogenicity for missense variants.^1^^,^^3^^,^^4^^,^^5^^,^^6^

Studies conducted in cancer susceptibility genes have shown that missense variants predicted to be destabilizing are likely non-functional.^5^^,^^7^^,^^8^ However, the reverse is not necessarily true, as missense variants not disturbing stability may still cause loss of function via other mechanisms, such as perturbing critical protein-protein or protein-ligand interactions.^9^

Many computational algorithms predict Gibbs free energy changes (ΔΔG) in protein folding (or protein interaction) upon mutation, with FoldX^10^ outperforming others in identifying pathogenic missense variants.^11^ Since FoldX uses Protein Data Bank atomic coordinate (PDB) files as input templates, the availability of experimental structures might limit the ability of structure to provide evidence. Recently, deep-learning algorithms have dramatically improved the accuracy of structure predictions from amino acid sequences, potentially expanding the clinical application of ΔΔG predictions to any protein of interest. AlphaFold2 has demonstrated outstanding performance, predicting the structure of protein globular domains with an accuracy matching X-ray crystallography, nuclear magnetic resonance (NMR), or cryogenic electron microscopy data.^12^^,^^13^ Assessment of AlphaFold2 models as templates to evaluate protein structural features show results that (for high-confidence predicted regions) consistently match or surpass those obtained with experimental templates.^14^

Recently, Google DeepMind has developed AlphaMissense, a machine-learning tool that utilizes the AlphaFold2-based structural context to predict pathogenicity for all human proteome missense variants.^15^ A study comparing predictions with data from multiplexed assays of variant effect (MAVE) suggests that AlphaMissense outperforms existing multiple-sequence-alignment conservation (meta-)predictors.^16^

ClinGen Variant Curation Expert Panel (VCEP) classification using gene-level specifications of the American College of Medical Genetics and Genomics/Association for Molecular Pathology (ACMG/AMP) framework,^17^ a process that has Food and Drug Administration (FDA) recognition, is increasingly acknowledged as a gold standard for clinical classification of germline variants in Mendelian disease genes. Despite the comprehensiveness of the framework, classification of rare missense variants remains challenging, and many variants remain VUSs after VCEP review. In the absence of compelling clinical and/or functional data, ACMG/AMP pathogenicity/benignity lines of evidence are restricted to the absence/rarity code PM2 with strength level decreased by ClinGen to “supporting” (SVI recommendation for absence/rarity PM2 v.1.0, available at https://clinicalgenome.org/working-groups/sequence-variant-interpretation), and computational codes PP3/BP4, based mostly on multiple-sequence-alignment conservation (meta-)predictors.^18^^,^^19^ Surprisingly, the current ACMG/AMP framework does not refer explicitly to structural features as a putative source of pathogenic/benign evidence and certainly does not incorporate structure-based pathogenicity and/or benignity codes.

**Figure 1:**
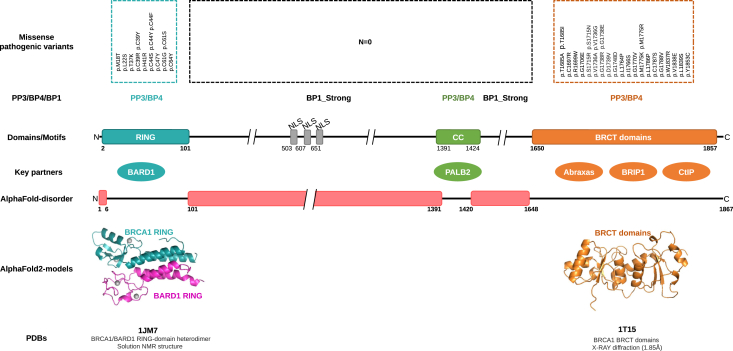
Summary of relevant structural, functional, and clinical annotations of the 1,867-aa BRCA1 protein The Missense pathogenic variants track displays all BRCA1 missense changes (*N* = 38) classified as pathogenic by the ClinGen ENIGMA BRCA1/2 VCEP (last consulted August 22, 2024) (scale not preserved). Note that pathogenic missense variants cluster at the RING and BRCT domains, with no pathogenic missense variants reported so far in other regions. The PP3/BP4/BP1 track summarizes ClinGen ENIGMA BRCA1/2 VCEP rules to apply ACMG/AMP predictive evidence to BRCA1 missense changes. Depending on BayesDel-noAF scores, PP3 (≥0.28) or BP4 (≤0.15) computational evidence is applied to missense variants targeting the RING, CC, or BRCT domains. By contrast, PP3/BP4 is not applied to missense variants targeting other regions (mostly, disordered regions). Instead, the BP1_Strong code is applied (regardless of computational predictions). The Domains/Motifs cartoon track represents BRCA1 conserved domains/motifs as defined by the ClinGen ENIGMA BRCA1/2 VCEP, with the RING and BRCT domains defined as clinically important functional domains and the CC (coiled-coil) motif as potentially clinically important (note that the precise boundaries of these domains might be slightly different according to other sources such as UniProt: P38398). The Key partners track shows BRCA1 key interacting proteins BARD1 (interacting with the RING domain), PALB2 (interacting with the CC motif), Abraxas, BRIP1 (also known as BACH1), and CtIP (the latter three interacting with the BRCT domains). The AlphaFold-disorder cartoon track represents BRCA1 disordered regions as deduced from the AlphaFold-2 model AF-P38398-F1 (p.LDDT score <70). The AlphaFold2-models track displays the BRCA1/BARD1 RING heterodimer and BRCT-domain AlphaFold2 models generated for this study. The PDBs track shows ID, descriptive name, and method for experimentally determined 3D structures used in this study.

Currently, the ClinGen ENIGMA *BRCA1* and *BRCA2* VCEP^20^ uses scores generated by the meta-predictor BayesDel^21^ to apply PP3/BP4 computational evidence to *BRCA1* missense variants located in the really interesting new gene (RING), coiled-coil (CC), and BRCA1 C-terminal (BRCT) domains (see Figure 1 for further details). Here, we tested the value of incorporating structural features, based on ΔΔG predictions and/or AlphaMissense pathogenicity scoring, for the ACMG/AMP classification of *BRCA1* missense variants. Overall, we show that regardless of the *BRCA1* functional domain analyzed (RING or BRCT), AlphaMissense outperforms BayesDel (PP3/BP4 moderate evidence strength applied to more variants), ΔΔG stratifies AlphaMissense evidence strength, and relative solvent accessibility (RSA) is a critical factor in evaluating the PP3/BP4 computational evidence (PP3/BP4 performing better for buried/partially buried residues than for exposed residues). In addition, our analysis suggests that regions other than the RING, CC, and BRCT domains might be functionally important.

## Material and methods

Starting with a *BRCA1* MAVE experiment reporting RNA and functional scores for 2,086 genetic variants annotated as missense,^22^ we generated a test cohort of 1,638 bona fide missense variants (536 targeting RING and 1,102 targeting BRCT residues) with associated functional data (MAVE dataset, see supplemental methods). The MAVE dataset included 1,182 variants scoring functional (FUNC, score > −0.748), 337 variants scoring non-functional (LoF, score < −1.328), and 119 variants scoring intermediate (INT, −1.328 < score < −0.748) (Table S1).

We predicted ΔΔG with FoldX5.0^10^ and six web-based methods. As input templates for FoldX5.0, we used experimental PDB files (ΔΔG^PDB^) and AlphaFold2 models (ΔΔG^AF^) (see supplemental methods for further details). All predicted ΔΔG values (*N* = 14,742) are shown in Table S1.

RSA was computed with the psa module of JOY.^23^ Computed RSA scores (*N* = 1,638) are shown in Table S1. Variants were stratified into those targeting buried (RSA < 30%), partially buried (30% ≤ RSA ≤ 60%), and exposed (RSA > 60%) residues. BayesDel scores (see Table S1) were retrieved from the database for non-synonymous SNPs’ functional predictions (dbNSFP) using the Ensembl Variant Effect Predictor (https://www.ensembl.org/Tools/VEP).^24^ Pre-computed AlphaMissense scores (see Table S1) were retrieved at console.cloud.google.com/storage/browser/dm_alphamissense.

Statistical analyses and graphical plots were performed using the statistical software R and packages ggplot2 (https://ggplot2.tidyverse.org), ROCR,^25^ and pROC.^26^ Statistical comparison of LoF, FUNC, and INT distributions was performed with non-parametric Wilcoxon test with the R ggpubr package (https://rpkgs.datanovia.com/ggpubr/). Differences between areas under the receiver-operating characteristics curve (auROCs) were assessed using the roc.test function of pROC through 10,000 bootstraps.^26^ The likelihood ratios toward pathogenicity or benignity provided by different AlphaMissense, ΔΔG, and BayesDel cutoff scores were assessed using an R-based online tool set up to simplify likelihood ratio (LR) calculations for bioinformatic prediction tool categories (gwiggins.shinyapps.io/lr_shiny). To transform LRs into evidence strengths, we followed recommendations arising from the Bayesian modeling of the ACMG/AMP rules.^27^ Accordingly, log_2_ LRs rounded to 1, 2, and 4 (point scores) equate to ACMG/AMP Supporting, Moderate, and Strong evidence strength, respectively.^28^ Stratified LR analysis was used to assign evidence weights to variants based on the combination of RSA, AlphaMissense, and ΔΔG (see supplemental methods for further details). Diagnostic test evaluation was performed online with MedCalc statistical software (www.medcalc.org/calc).

We retrieved variant-level counts for 122 bona fide missense variants targeting the BRCA1 RING or BRCT domains from the Breast Cancer After Diagnostic Gene Sequencing (BRIDGES) breast cancer association study^29^ (see supplemental methods).

## Results

We have evaluated missense variants targeting the *BRCA1* clinically relevant domains RING or BRCT and for which MAVE functional data were available (Table S1). In total, we have evaluated 1,638 missense variants (536 targeting the RING domain and 1,102 targeting the BRCT domain) with MAVE data indicating LoF (*n* = 337), INT (*n* = 119), or FUNC (*n* = 1,182). Stratification by functional domain did not reveal differences, with variants demonstrating impaired activity (LoF + INT) representing 28% of the variants in both domains. In contrast, stratification by RSA revealed major differences, with impaired activity variants representing 42% of the variants targeting buried residues (*n* = 879), 16% of the variants targeting partially buried residues (*n* = 331), and 7% of the variants targeting exposed residues (*n* = 428). We observed this trend in both domains, but it appeared most striking in the subgroup of variants targeting the BRCT domain. Table S2 and Figures S1A and S1B summarize relevant features of the MAVE dataset stratified by domain, RSA, functional category, or residue subtype (within the RING domain).

Using PDB files as input templates (ΔΔG^PDB^), FoldX5.0 predicted, on average, a strong destabilizing effect for LoF variants (ΔΔG = +5.77 kcal/mol), a mild destabilizing effect for FUNC variants (ΔΔG = +0.64 kcal/mol), and an intermediate destabilizing effect for INT variants (ΔΔG = +3.09 kcal/mol). Using AlphaFold2 models (ΔΔG^AF^), we observed similar results, with average destabilizing impacts of +6.55 kcal/mol for LoF, +1.04 kcal/mol for FUNC, and +3.41 kcal/mol for INT variants. ΔΔG^PDB^ (or ΔΔG^AF^) stratification per domain or per RSA suggested that the average destabilizing effect is higher both for variants targeting RING residues and for variants targeting buried residues (Table S2; Figures S2 and S3). Overall, the data suggest that MAVE functional class stratification by ΔΔG is better for variants targeting buried/partially buried residues (RSA ≤ 60%) than for variants targeting exposed residues (RSA > 60%). In the latter case, we did not observe statistically significant differences in the average ΔΔG^PDB^ (or ΔΔG^AF^) value of INT and FUNC variants (Figures S2 and S3). Stratification by functional domain (RING vs. BRCT) did not reveal major differences (Figures S2 and S3).

Interestingly, AlphaMissense and BayesDel showed similar trends, with INT variants displaying intermediate scores, variants targeting RING residues scoring higher than variants targeting BRCT residues, and variants targeting buried residues scoring higher than others (Table S2). As observed for ΔΔG, MAVE functional class stratification by AlphaMissense was better for variants targeting buried/partially buried residues (RSA ≤ 60%) than for variants targeting exposed residues (RSA > 60%), with no major differences between RING and BRCT domains (Figure S4). BayesDel performed similarly, with some evidence that it might outperform ΔΔG and AlphaMissense at variants targeting exposed (RSA>60%) residues (Figure S5).

Overall, the (negative) correlation of AlphaMissense with MAVE functional scores (*r* = −0.67) is higher compared to the correlation with BayesDel (*r* = −0.61), ΔΔG^AF^(*r* = −0.55), and ΔΔG^PDB^ (*r* = −0.51). Note that ΔΔG^AF^ correlated better than ΔΔG^PDB^ (Figure S6).

**Figure 2:**
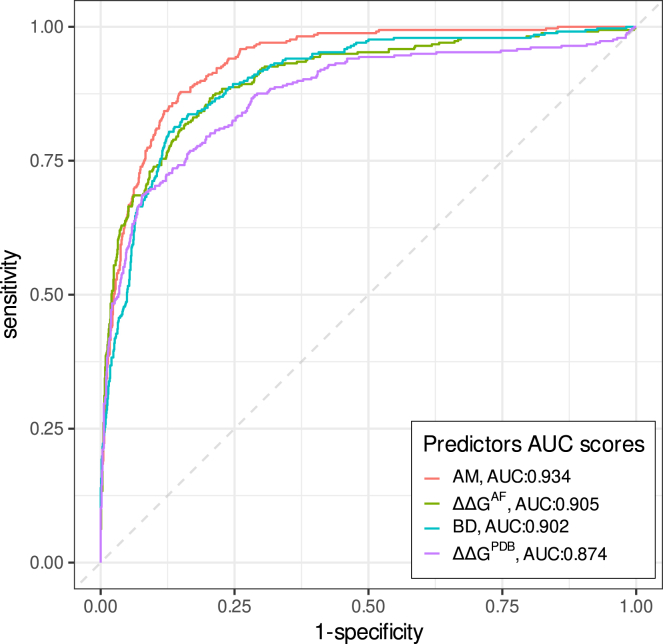
AlphaMissense, ΔΔG^PDB^, ΔΔG^AF^, and BayesDel performance at discriminating LoF and FUNC variants at the RING and BRCT domains The figure displays ROC plots and the corresponding auROC value. Overall, AlphaMissense (AM) provides the best discrimination. ΔΔG^AF^ outperforms ΔΔG^PDB^.

We next tested the performance of ΔΔG^AF^, ΔΔG^PDB^, AlphaMissense, and BayesDel to distinguish LoF (used here as a proxy for pathogenicity) from FUNC variants (proxy for benignity). For this analysis, we filtered out INT variants (the association of partial activity with disease predisposition is unclear), restricting our analysis to an MAVE cohort of 1,519 variants (337 LoF and 1,182 FUNC). Overall, AlphaMissense (auROC = 0.93) outperformed ΔΔG^AF^ (auROC = 0.91, *p* = 0.003), BayesDel (auROC = 0.90, *p* = 0.0001), and ΔΔG^PDB^ (auROC = 0.87, *p* = 1.2 × 10^−6^) (Figure 2).

Six web-based ΔΔG predictors perform poorly, with auROCs ranging from 0.64 (CUPSAT) to 0.78 (INPS3D), and correlation with MAVE functional scores ranging from *r* = −0.21 (CUPSAT) to *r* = −0.44 (INSP3D) (Figures S6 and S7).

Stratification by functional domain showed that: (1) AlphaMisense and ΔΔG^AF^ (each with auROC = 0.94) outperformed BayesDel (auROC = 0.92, *p* not significant) and ΔΔG^PDB^ (auROC = 0.86, *p* = 0.003) at discriminating LoF and FUNC variants at the RING domain (*N* = 408); and (2) AlphaMissense (auROC = 0.95) outperformed BayesDel (auROC = 0.90, *p* = 1.2 × 10^−5^), ΔΔG^AF^ (auROC = 0.89, *p* = 3.9 × 10^−6^), and ΔΔG^PDB^ (auROC = 0.88, *p* = 3.7 × 10^−8^) at discriminating LoF and FUNC variants in the BRCT (*N* = 1,032) domain. Note that overall, ΔΔG^AF^ outperformed ΔΔG^PDB^, mostly due to a better performance in the RING domain (Figure S8).

**Figure 3:**
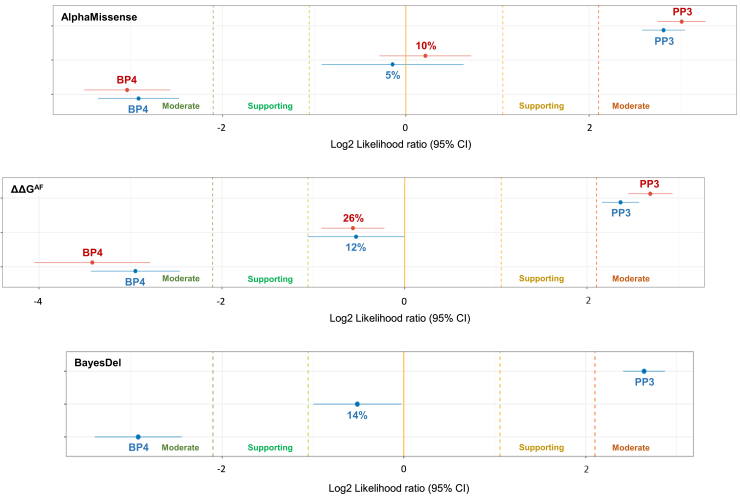
PP3/BP4 computational evidence strengths provided by AlphaMissense, ΔΔG^AF^, and BayesDel AlphaMissense evidence strengths using ≤0.6 (BP4)//≥0.8 (PP3) (red) or ≤0.65 (BP4)//≥0.75 (PP3) (blue) thresholds (top). ΔΔG^AF^ evidence strengths using ≤+1 kcal/mol (BP4)//≥+3 kcal/mol (PP3) (red) or ≤+1.5 kcal/mol (BP4)//≥+2.5 kcal/mol (PP3) (blue) thresholds (middle). BayesDel evidence strength with ClinVar ENIGMA BRCA1/2 VCEP recommended thresholds (bottom). Percent of variants falling in the non-informative score range. log_2_ LR calculations and plot (including Log2 LR 95% confidential intervals) generated at gwiggins.shinyapps.io/lr_shiny/.

We next evaluated the performance of computational evidence based on AlphaMissense or ΔΔG (FoldX5.0 predictions) and how it compared with the BayesDel-based PP3/BP4 evidence currently specified for the ACMG/AMP classification of *BRCA1* missense variants. To start with, we analyzed the evidence strength against (or toward) pathogenicity provided by AlphaMissense at various thresholds, including an uninformative score-range category (i.e., bioinformatic evidence not applicable) centered at the optimal binary cutpoint. A trade-off iterative process aimed at maximizing strength of evidence and minimizing the proportion of variants in the uninformative range led us to conclude that AlphaMissense performed well with benignity/pathogenicity thresholds set near 0.7. Applying ≤0.65 (benignity) and ≥0.75 (pathogenicity) thresholds, 1,015 variants would receive evidence in the benign direction (log_2_ LR = −2.91) and 426 variants in the pathogenic direction (log_2_ LR = +2.81), while only 78 variants (5%) would fall in the uninformative score range (Table 1 and Figure 3).

**Table 1:** PP3/BP4 computational evidence based on AlphaMissense, ΔΔG^AF^, ΔΔG^PDB^, or BayesDel at different benignity and pathogenicity cutoff thresholds

	**Benignity evidence (BP4)**		**No bioinformatic code applicable**	**Pathogenicity evidence (PP3)**	
	**Threshold**	**Evidence strength, log**_**2**_**LR (95% CI)**		**Threshold**	**Evidence strength, log**_**2**_**LR (95% CI)**
AM	≤0.34^a^	−4.603 (−5.535 to −3.671)	12%	≥0.56^a^	+2.083 (+1.919 to +2.247)
	≤0.60	−3.038 (−3.508 to −2.569)	10%	≥0.80	+3.007 (+2.746 to +3.269)
	≤0.65	−2.914 (−3.354 to −2.474)	5%	≥0.75	+2.810 (+2.578 to +3.042)
ΔΔG^AF^^b^	≤+1.0	−3.417 (−4.051 to −2.784)	26%	≥+3.0	+2.691 (+2.448 to +2.933)
	≤+1.5	−2.946 (−3.433 to −2.456)	8%	≥+2.5	+2.367 (+2.164 to +2.570)
ΔΔG^PDB^^b^	≤+1.0	−2.663 (−3.135 to −2.191)	26%	≥+3.0	+2.780 (+2.515 to +3.045)
	≤+1.5	−2.264 (−2.632 to −1.895)	12%	≥+2.5	+2.309 (+2.093 to +2.525)
BD	≤0.15^c^	−2.923 (−3.401 to −2.444)	14%	≥0.28^c^	+2.643 (+2.415 to +2.872)

a Generic thresholds as per AlphaMissense developers (note that benignity evidence strength is very strong but pathogenicity evidence strength is weaker, and rate of variants in the uninformative score range is high).

b FoldX5.0 predictions.

c BP4/PP3 BRCA1 VCEP thresholds.

A similar trade-off iterative process led us to conclude that ΔΔG^AF^ performed well with benignity/pathogenicity thresholds set near +2.0 kcal/mol. Applying ≤+1.5 and ≥+2.5 kcal/mol thresholds, 869 variants received evidence of benignity and 462 variants received evidence of pathogenicity (log_2_ LR = −2.95 and +2.37, respectively), with 12% of the variants falling in the uninformative score range. See Table 1 and Figure 2 for further details. Of note, ΔΔG^PDB^ did not outperform ΔΔG^AF^ either in benignity/pathogenicity evidence strengths (log_2_ LRs) or in the rate of variants in the uninformative range (Table 1).

Using current *BRCA1* VCEP BayesDel thresholds for BP4 and PP3, 883 missense variants receive evidence of benignity (and 420 evidence of pathogenicity) (log_2_ LR = −2.92 and +2.64), and 216 variants (14%) fall in the uninformative score range (0.15 < BayesDel < 0.28). See Table 1 and Figure 2 for further details.

In brief, AlphaMissense, ΔΔG (FoldX5.0 predictions), and BayesDel provide evidence toward pathogenicity (+2 < log_2_ LR < +4) and benignity (−4 < log_2_ LR < −2) with evidence strengths in the moderate to strong range. A diagnostic test evaluation does not reveal major differences between the tools (Table 2).

**Table 2:** Diagnostic test evaluation

**Threshold**	**Sensitivity**	**Specificity**	**PPV**	**NPV**	**Accuracy**
**AM**					

≥0.75	84.3 (79.9–88.0)	88.0 (86.0–89.8)	66.7 (63.0–70.1)	95.2 (93.8–96.2)	87.2 (85.4–88.8)

**ΔΔG^AF^**^a^					

≥+3	89.1 (84.9–92.5)	85.9 (83.5–88.0)	64.0 (60.2–67.6)	96.6 (92.3–97.5)	86.6 (84.6–88.4)
≥+2.5	81.6 (77.1–85.6)	84.2 (81.2–86.2)	59.5 (56.1–62.8)	94.1 (92.8–95.3)	83.7 (81.7–85.4)

**ΔΔG^PDB^**^a^					

≥+3	87.7 (83.1–91.4)	85.9 (83.4–88.2)	63.7 (59.7–67.6)	96.1 (94.7–97.2)	86.3 (84.2–88.3)
≥+2.5	74.2 (69.2–78.8)	85.0 (82.9–87.0)	58.5 (54.9–62.1)	92.0 (90.6–93.3)	82.6 (80.6–84.5)

**BD**					

≥0.28	79.8 (75.1–84.0)	87.2 (85.2–89.1)	64.1 (60.3–67.6)	93.8 (92.5–94.9)	85.6 (83.7–87.3)

With the indicated thresholds, the table shows the performance of AlphaMissense, ΔΔG^AF^, ΔΔG^PDB^, and BayesDel discriminating MAVE LoF variants. AM, AlphaMissense; BD, BayesDel; PPV, positive predictive value; NPV, negative predictive value.

a FoldX5.0 predictions.

We conclude that an AlphaMissense-based PP3/BP4 evidence would outperform BayesDel-based (or ΔΔG-based) PP3/BP4 evidence, since it would provide similar pathogenicity and benignity evidence strengths but with a lower rate of variants in the uninformative score range (Table 1). Stratification by functional domain (RING vs. BRCT) did not reveal major differences, with all three computational tools supporting moderate to strong pathogenicity/benignity evidence strength (and AlphaMissense providing a lower rate of variants in the uninformative score range) in both domains (Table S3).

**Figure 4:**
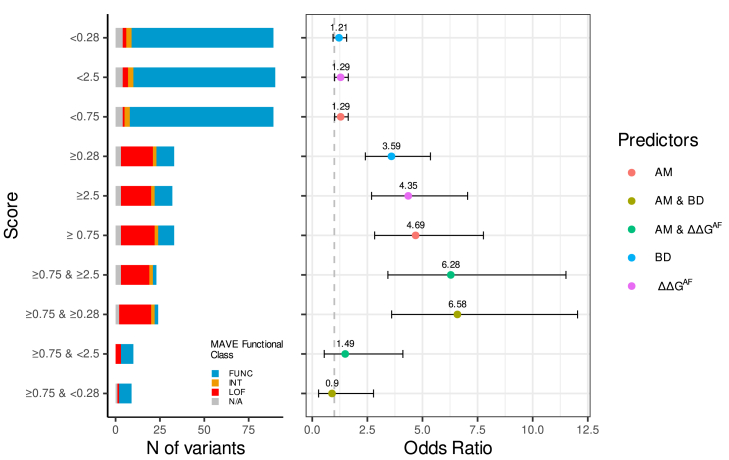
BRIDGES-based breast cancer risk estimates (burden analysis) stratified by computational scores The plot on the right displays breast cancer ORs (and 95% confidential intervals) for variants ≥ (<) the indicated cutoff. The plot on the left shows corresponding distribution of MAVE functional classes. Note that higher ORs correspond to higher proportion of MAVE LoF variants. AM, AlphaMissense; BD, BayesDel; N/A, BRIDGES variants not assessed in MAVE.

Burden-type association analysis (see material and methods) confirmed that, on average, AlphaMissense ≥ 0.75 (odds ratio [OR] = 4.69), ΔΔG^AF^ ≥ 2.5 kcal/mol (OR = 4.35), ΔΔG^PDB^ ≥ 2.5 kcal/mol (OR = 3.62), and BayesDel ≥ 0.28 (OR = 3.59) each identify *BRCA1* missense variants with clinically actionable BC risk levels.^30^ Equally relevant, the analysis confirmed that variants scoring below PP3 thresholds are not associated with clinically actionable risk levels (ORs < 1.3) (Table 3 and Figure 4).

**Table 3:** BRIDGES-based breast cancer risk estimates (burden analysis) stratified by AlphaMissense, ΔΔG^AF^, ΔΔG^PDB^, and BayesDel scoring

**Threshold**	**Unique *BRCA1* missense variants**	**BC**^a^	**Controls**^b^	**OR (95% CI)**	***p***
**AM**					

≥0.75	33	94	18	^∗^4.69 (2.83–7.76)	^∗^4.15 × 10^−9^
0.65 < AM < 0.75	7	22	16	1.23 (0.68–2.35)	0.53
≤0.65	82	146	101	1.30 (1.01–1.66)	0.04

**ΔΔG^AF^**^c^					

≥+2.5	32	97	20	^∗^4.35 (2.69–7.05)	^∗^4.52 × 10^−9^
+1.5 < ΔΔG < +2.5	23	43	24	1.61 (0.98–2.65)	0.06
≤+1.5	67	122	91	1.20 (0.92–1.58)	0.18

**ΔΔG^PDB^**^c^					

≥+2.5	35	121	30	^∗^3.62 (2.43–5.40)	^∗^7.04 × 10^−10^
+1.5 < ΔΔG < +2.5	19	16	15	0.96 (0.47–1.93)	1.08
≤+1.5	68	125	90	1.25 (0.95–1.63)	0.11

**BD**					

≥0.28	33	120	30	^∗^3.59 (2.41–5.36)	^∗^9.25 × 10^−10^
0.15 < BD < 0.28	19	54	30	1.61(1.03–2.52)	0.04
≤0.15	70	88	75	1.05 (0.77–1.43)	0.75

Asterisk (^∗^) indicates statistically significant. See Figure 3 and Table S4 for further information.

a 53,572 population-based breast cancer (BC) cases.

b 48,048 matched controls.

c FoldX5.0 predictions

Stratification by domain (RING vs. BRCT) did not reveal major differences (Table S4). Further, the analysis revealed that ΔΔG (or BayesDel) scoring provides clinically relevant risk stratification to AlphaMissense ≥ 0.75 variants. On average, concordant scores identified high-risk variants (ORs > 6.0), while discordant scores identified variants not associated with risk (Figure 4).

Since average ΔΔG, AlphaMissense, and BayesDel scoring of LoF, INT, and FUNC variants was influenced by RSA, we suspected that pathogenicity/benignity evidence strength might be RSA dependent too. To test the hypothesis, we first analyzed ΔΔG^AF^ discriminating performance stratifying target residues into buried (RSA ≤ 30%), partially buried (30% < RSA ≤ 60%), and exposed (RSA > 60%). Overall, we observe that ΔΔG^AF^ (≥+2.5 kcal/mol) provides evidence toward pathogenicity regardless of RSA. By contrast, ΔΔG^AF^ (≤+1.5 kcal/mol) provides evidence toward benignity for buried/partially buried but not for exposed residues (Table S5). ΔΔG^PDB^-based analysis provided similar results (Table S5). Interestingly, the target residue RSA influenced AlphaMissense-based and BayesDel-based computational evidence similarly. For variants targeting exposed residues, neither AlphaMissense nor BayesDel provided statistically significant evidence toward benignity (Table S5). This RSA influence on computational evidence was observed in both the RING and BRCT domains (Table S6).

We next explored the possibility of combining AlphaMissense with other computational scores to maximize PP3/BP4 code utility in the ACMG/AMP classification of *BRCA1* missense variants. Since RSA, ΔΔG^PDB^, and ΔΔG^AF^ correlations with AlphaMissense are moderate (*r* = −0.49, +0.46, and +0.50, respectively), while BayesDel correlation (*r* = +0.74) is high (Figure S6), we focused our analysis on combining AlphaMissense with RSA and ΔΔG. We tested different approaches. Computational evidence based on AlphaMissense/ΔΔG concordance provided Strong level of pathogenic and benign evidence at the cost of a high rate of variants (31%) in the uninformative score range (Figure S9). RSA stratification revealed that AlphaMissense/ΔΔG concordance does not provide statistically significant benignity evidence at even Supporting strength to variants targeting exposed residues (Figure S10), confirming the relevance of RSA stratification in computational scoring. In our hands, cascade stratification by RSA, AlphaMissense, and ΔΔG performed well for variants targeting buried/partially buried residues, providing pathogenic/benign evidence strength stratification into Strong and Moderate, with only 16% of variants falling in the uninformative score range (Figure S11). Regarding variants targeting exposed residues, cascade stratification improved the rate of variants with evidence of pathogenicity (Moderate or Strong strength) but failed to provide evidence of benignity with Supporting strength (Figure S11).

Finally, we performed a protein-wide AlphaMissense analysis to investigate whether pathogenic *BRCA1* missense variants might cluster in regions other than the RING and BRCT domains. The analysis suggested that, in addition to CC residues 1,391–1,424 (already highlighted as “potentially clinically important” in the BRCA1/2 VCEP specifications, see Figure 1), BRCA1 regions spanning residues 127–133, 180–185, 378–386, 458–465, 515–519, and 853–869 might be candidates for further investigation and potentially important for impact on protein function (Figure S12).

## Discussion

In the present study, we have evaluated the AlphaMissense-based and ΔΔG-based PP3/BP4 computational evidence contribution to the ACMG/AMP classification of *BRCA1* missense variants located at the RING and BRCT domains and how these compare with the current BayesDel-based PP3/BP4 applied by the *BRCA1* VCEP. The analysis is based on a *BRCA1* MAVE study,^22^ using LoF and FUNC categories as proxies for pathogenicity and benignity. We showed that, overall, PP3/BP4 evidence strengths provided by AlphaMissense, ΔΔG, and BayesDel are similar (in all cases in the moderate to strong range), but AlphaMisense outperformed ΔΔG and BayesDel in the lower proportion of missense variants with uninformative scores (PP3/BP4 not applicable). Our data are compatible with recent studies indicating that AlphaMissense correlates better with MAVE data for five genes (*DDX3X*, *BRCA1*, *MSH2*, *PTEN*, and *KCNQ4*) than earlier prediction algorithms, including BayesDel.^16^

AlphaMissense developers recommend benignity/pathogenicity default score thresholds of ≤0.34 and ≥0.56, specifying that (depending on the desired use) different gene-level cutoffs may improve trade-offs and general performance.^15^ Here, we showed that AlphaMissense achieves best PP3/BP4 performance with benignity/pathogenicity score thresholds set much higher, at ≤0.65 and ≥0.75, respectively.

ΔΔG achieved the best PP3/BP4 performance with thresholds near +2 kcal/mol. This is in agreement with previous studies in other proteins indicating that ΔΔG values >+3 kcal/mol predict pathogenicity.^7^^,^^9^ Interestingly, ΔΔG^AF^ and ΔΔG^PDB^ performed similarly. To what extent AlphaFold2 models can substitute experimental structures in predicting the impact of missense variants is currently a matter of debate.^31^^,^^32^

Our data on BRCA1 suggest that AlphaFold2-generated models may replace experimental PDBs in the very specific task of applying FoldX5.0-predicted ΔΔG-based PP3/BP4 computational evidence. The observation is remarkable but not necessarily true for other proteins and, in particular, for proteins lacking experimental structural data (it is possible to argue that ΔΔG^AF^ performs well for human BRCA1 RING and BRCT domains precisely because existing experimental data contribute to generation of high-quality AlphaFold2 models).

Perhaps more relevant is the finding that, in the subset of variants targeting the BRCT domain, ΔΔG^AF^ and ΔΔG^PDB^ performed similarly, while ΔΔG^AF^ outperformed ΔΔG^PDB^ in the subset of variants targeting the RING domain. The difference might be related to the fact that the BRCT structure PDB: 1T15 was solved by X-ray diffraction,^33^ while the human BRCA1/BARD1 RING heterodimer structure PDB: 1JM7 was solved by solution NMR.^34^ First, it is known that FoldX ΔΔG prediction accuracy is higher in crystallographic structures.^10^ Second, on computing ΔΔG^PDB^ for variants targeting the RING domain, we used as template the best ranked out of 14 conformers. Perhaps the corresponding AlphaFold2 model reflects the average conformation more accurately than each individual NMR conformer, thus generating more accurate ΔΔG predictions. That said, it is worth mentioning that computing ΔΔG^AF^ at the RING domain was challenging and that initial attempts using a model of the BRCA1 RING monomer (equivalent to AF-P38398-F1) performed poorly (data not shown). We achieve good ΔΔG^AF^ PP3/BP4 performance at the RING domain only after creating a model of the BRCA1/BARD1 heterodimer that was latterly modified to introduce four critical Zn^2+^ atoms (supplemental methods). In this regard, is worth mentioning that AlphaFold3 (an AlphaFold2 update that simplifies the modeling of metalloproteins and other biomolecular entities) has been released very recently.^35^

Our study points to RSA as a relevant factor to evaluate ΔΔG-based PP3/BP4 performance. Overall, the data indicate that ΔΔG provides computational pathogenicity evidence (PP3) regardless of RSA but benignity evidence (BP4) only for variants targeting buried/partially buried (RSA ≤ 60%) residues. The finding is not without a rationale. ΔΔG identifies pathogenic variants only if causing reduced thermodynamic stability, i.e., predicts benignity if stability is not impacted. Since most core pathogenic variants are likely to lead to loss of stability, the absence of destabilization is predictive of benignity for core missense variants. By contrast, surface pathogenic variants may act through protein destabilization (explaining that ΔΔG provides pathogenicity evidence) but also through alternative mechanisms such as impairing critical protein-protein interactions (without affecting folding/stability).^9^^,^^14^ Consequently, absence of destabilization does not necessarily guarantee functionality for protein changes at the surface (explaining that ΔΔG does not provide benignity evidence).

In principle, AlphaMissense and BayesDel identify pathogenic variants regardless of the underlying driving mechanism, so that RSA is not an obvious factor in modulating PP3/BP4 performance. Yet we find somewhat unexpectedly that both tools, similarly to ΔΔG, are RSA modulated, and that neither of them provides computational benignity evidence (BP4) to variants targeting exposed residues. The finding probably reflects the fact that, indirectly, AlphaMissense and BayesDel (and other computational tools) capture stability-related features (unsurprisingly, since tools have been trained to discriminate pathogenic and benign missense variants and most missense pathogenic variants impact stability). At any rate, the putative relevance of RSA on evaluating PP3/BP4 computational evidence warrants further analyses in other clinically relevant proteins.

As expected, AlphaMissense shows a positive correlation with ΔΔG and BayesDel scores (Figure S6). The correlation with BayesDel is strong (*r* = 0.74), but the correlation with ΔΔG is weaker (*r* = 0.46), opening the possibility of independent AlphaMissense and ΔΔG contributions to variant classification. Indeed, we show that combined use of AlphaMissense and ΔΔG (and RSA) adds granularity to the pathogenicity and benignity evidence strengths provided by computational tools (Figures S9–S11).

Apart from increasing PP3/BP4 evidence strength granularity, the combined analysis with AlphaMissense and ΔΔG may provide additional information. For instance, we speculate that AlphaMissense and ΔΔG discordance may encapsulate relevant information contributing to the identification of INT missense variants (INT variants might be associated with AlphaMissense and ΔΔG discordance; Figure S13). Further, AlphaMissense pathogenicity scores do not provide direct mechanistic interpretability. Combining analysis with ΔΔG may provide such interpretability, contributing to the mapping of protein regions/residues with relevant functions other than protein stability (Figures S14 and S15).

Finally, we think that our study (limited to missense variants in two functional domains of the tumor-suppressor gene *BRCA1*) illustrates some general principles and recommendations that might be relevant in evaluating PP3/BP4 computational evidence in other proteins.

(1) PP3/BP4 assessment benefits from a structure-based analysis.
(2) AlphaMissense likely outperforms earlier computational tools, but optimal gene-specific pathogenic/benign cutoffs might be very different from the generic cutoffs originally proposed.
(3) Regardless of the computational tool under assessment, we recommend a comprehensive evaluation of performance stratified by RSA (anticipating that meeting computational benignity evidence BP4 for changes at the protein surface will be challenging for many proteins).
(4) ΔΔG (FoldX5.0 predictions) adds evidence strength granularity to AlphaMissense-based computational evidence.
(5) If structural data available for the protein of interest is based on NMR, we recommend considering ΔΔG (FoldX5.0 predictions) based on an AlphaFold model as an alternative (provided that the model reflects the physiologically relevant monomeric, homo[hetero]-dimeric, or multimeric structure, and that, in the case of modeling metalloproteins, metal ions have been added).

To what extent these five principles are truly generic (applicable to other proteins), or BRCA1 specific, warrants further studies.

## Data and code availability

The datasets generated during this study are available in Table S1.

## Acknowledgments

M.d.l.H.’s research activity has been funded by 10.13039/501100004587Instituto de Salud Carlos III grants PI20/00110 and PI24/00267 co-funded by the 10.13039/501100000780European Union (10.13039/501100008530ERDF/ESF, “A way to make Europe”/“Investing in your future”) and a 10.13039/100000002National Institutes of Health (NIH) grant 5U24CA258058-02. N.R. and A.B.-S. were supported by 10.13039/501100000925NHMRC funding (APP177524). C.F. was supported by funding from the 10.13039/501100001026National Breast Cancer Foundation, Australia (IIRS-21-102).

## Author contributions

L.R.-M.: methodology, formal analysis, investigation, data curation, writing – review & editing, and visualization. N.R.: methodology and writing – review & editing. A.E.-S.: data curation and writing – review & editing. C.F.: methodology and writing – review & editing. A.G.-S.: investigation and writing – review & editing. M.J.V.: methodology and writing – review & editing. A.C.: methodology and writing – review & editing. M.E.R.: conceptualization, writing – review & editing, and project administration. K.R.: methodology and writing – review & editing. P.P.-S.: funding acquisition and writing – review & editing. A.B.S.: conceptualization, methodology, and writing – review & editing. M.d.l.H.: conceptualization, methodology, formal analysis, writing – original draft, writing – review & editing, and funding acquisition.

## Declaration of interests

M.J.V. is an employee of Ambry Genetics. A.C. is an employee of Ambry Genetics. M.E.R. is an employee of Ambry Genetics.

## Web resources

Ensembl Variant Effect Predictor, https://www.ensembl.org/Tools/VEP
ggplot2, https://ggplot2.tidyverse.org
MedCalc statistical software, www.medcalc.org/calc
R ggpubr package, https://rpkgs.datanovia.com/ggpubr/
SVI recommendation for absence/rarity PM2—version 1.0, https://clinicalgenome.org/working-groups/sequence-variant-interpretation

## Supplemental information

Document S1. Figures S1–S15, Tables S2–S6, and supplemental methods

Table S1. Structure-informed scores for 1,638 *BRCA1* missense variants with functional data

Document S2. Article plus supplemental information

## References

[1] Stein A., Fowler D.M., Hartmann-Petersen R., Lindorff-Larsen K. (2019). Biophysical and Mechanistic Models for Disease-Causing Protein Variants. Trends Biochem. Sci..

[2] Schaafsma G.C.P., Vihinen M. (2017). Large differences in proportions of harmful and benign amino acid substitutions between proteins and diseases. Hum. Mutat..

[3] Casadio R., Vassura M., Tiwari S., Fariselli P., Luigi Martelli P. (2011). Correlating disease-related mutations to their effect on protein stability: a large-scale analysis of the human proteome. Hum. Mutat..

[4] Pal L.R., Moult J. (2015). Genetic Basis of Common Human Disease: Insight into the Role of Missense SNPs from Genome-Wide Association Studies. J. Mol. Biol..

[5] Petrosino M., Novak L., Pasquo A., Chiaraluce R., Turina P., Capriotti E., Consalvi V. (2021). Analysis and Interpretation of the Impact of Missense Variants in Cancer. Int. J. Mol. Sci..

[6] Redler R.L., Das J., Diaz J.R., Dokholyan N.V. (2016). Protein Destabilization as a Common Factor in Diverse Inherited Disorders. J. Mol. Evol..

[7] Nielsen S.V., Stein A., Dinitzen A.B., Papaleo E., Tatham M.H., Poulsen E.G., Kassem M.M., Rasmussen L.J., Lindorff-Larsen K., Hartmann-Petersen R. (2017). Predicting the impact of Lynch syndrome-causing missense mutations from structural calculations. PLoS Genet..

[8] Reza M.N., Ferdous N., Emon M.T.H., Islam M.S., Mohiuddin A.K.M., Hossain M.U. (2021). Pathogenic genetic variants from highly connected cancer susceptibility genes confer the loss of structural stability. Sci. Rep..

[9] Høie M.H., Cagiada M., Beck Frederiksen A.H., Stein A., Lindorff-Larsen K. (2022). Predicting and interpreting large-scale mutagenesis data using analyses of protein stability and conservation. Cell Rep..

[10] Schymkowitz J., Borg J., Stricher F., Nys R., Rousseau F., Serrano L. (2005). The FoldX web server: an online force field. Nucleic Acids Res..

[11] Gerasimavicius L., Liu X., Marsh J.A. (2020). Identification of pathogenic missense mutations using protein stability predictors. Sci. Rep..

[12] Tunyasuvunakool K., Adler J., Wu Z., Green T., Zielinski M., Žídek A., Bridgland A., Cowie A., Meyer C., Laydon A. (2021). Highly accurate protein structure prediction for the human proteome. Nature.

[13] Jumper J., Evans R., Pritzel A., Green T., Figurnov M., Ronneberger O., Tunyasuvunakool K., Bates R., Žídek A., Potapenko A. (2021). Highly accurate protein structure prediction with AlphaFold. Nature.

[14] Akdel M., Pires D.E.V., Pardo E.P., Jänes J., Zalevsky A.O., Mészáros B., Bryant P., Good L.L., Laskowski R.A., Pozzati G. (2022). A structural biology community assessment of AlphaFold2 applications. Nat. Struct. Mol. Biol..

[15] Cheng J., Novati G., Pan J., Bycroft C., Žemgulytė A., Applebaum T., Pritzel A., Wong L.H., Zielinski M., Sargeant T. (2023). Accurate proteome-wide missense variant effect prediction with AlphaMissense. Science.

[16] Ljungdahl A., Kohani S., Page N.F., Wells E.S., Wigdor E.M., Dong S., Sanders S.J. (2023). AlphaMissense is better correlated with functional assays of missense impact than earlier prediction algorithms. bioRxiv.

[17] Richards S., Aziz N., Bale S., Bick D., Das S., Gastier-Foster J., Grody W.W., Hegde M., Lyon E., Spector E. (2015). Standards and guidelines for the interpretation of sequence variants: a joint consensus recommendation of the American College of Medical Genetics and Genomics and the Association for Molecular Pathology. Genet. Med..

[18] Tian Y., Pesaran T., Chamberlin A., Fenwick R.B., Li S., Gau C.L., Chao E.C., Lu H.M., Black M.H., Qian D. (2019). REVEL and BayesDel outperform other in silico meta-predictors for clinical variant classification. Sci. Rep..

[19] Pejaver V., Byrne A.B., Feng B.J., Pagel K.A., Mooney S.D., Karchin R., O'Donnell-Luria A., Harrison S.M., Tavtigian S.V., Greenblatt M.S. (2022). Calibration of computational tools for missense variant pathogenicity classification and ClinGen recommendations for PP3/BP4 criteria. Am. J. Hum. Genet..

[20] Parsons M.T., de la Hoya M., Richardson M.E., Tudini E., Anderson M., Berkofsky-Fessler W., Caputo S.M., Chan R.C., Cline M.S., Feng B.J. (2024). Evidence-based recommendations for gene-specific ACMG/AMP variant classification from the ClinGen ENIGMA BRCA1 and BRCA2 Variant Curation Expert Panel. Am. J. Hum. Genet..

[21] Feng B.J. (2017). PERCH: A Unified Framework for Disease Gene Prioritization. Hum. Mutat..

[22] Findlay G.M., Daza R.M., Martin B., Zhang M.D., Leith A.P., Gasperini M., Janizek J.D., Huang X., Starita L.M., Shendure J. (2018). Accurate classification of BRCA1 variants with saturation genome editing. Nature.

[23] Mizuguchi K., Deane C.M., Blundell T.L., Johnson M.S., Overington J.P. (1998). JOY: protein sequence-structure representation and analysis. Bioinformatics.

[24] McLaren W., Gil L., Hunt S.E., Riat H.S., Ritchie G.R.S., Thormann A., Flicek P., Cunningham F. (2016). The Ensembl Variant Effect Predictor. Genome Biol..

[25] Sing T., Sander O., Beerenwinkel N., Lengauer T. (2005). ROCR: visualizing classifier performance in R. Bioinformatics.

[26] Robin X., Turck N., Hainard A., Tiberti N., Lisacek F., Sanchez J.C., Müller M. (2011). pROC: an open-source package for R and S+ to analyze and compare ROC curves. BMC Bioinf..

[27] Tavtigian S.V., Greenblatt M.S., Harrison S.M., Nussbaum R.L., Prabhu S.A., Boucher K.M., Biesecker L.G., ClinGen Sequence Variant Interpretation Working Group ClinGen SVI (2018). Modeling the ACMG/AMP variant classification guidelines as a Bayesian classification framework. Genet. Med..

[28] Tavtigian S.V., Harrison S.M., Boucher K.M., Biesecker L.G. (2020). Fitting a naturally scaled point system to the ACMG/AMP variant classification guidelines. Hum. Mutat..

[29] Dorling L., Carvalho S., Allen J., González-Neira A., Luccarini C., Wahlström C., Pooley K.A., Parsons M.T., Fortuno C., Breast Cancer Association Consortium (2021). Breast Cancer Risk Genes - Association Analysis in More than 113,000 Women. N. Engl. J. Med..

[30] Spurdle A.B., Greville-Heygate S., Antoniou A.C., Brown M., Burke L., de la Hoya M., Domchek S., Dörk T., Firth H.V., Monteiro A.N. (2019). Towards controlled terminology for reporting germline cancer susceptibility variants: an ENIGMA report. J. Med. Genet..

[31] Buel G.R., Walters K.J. (2022). Can AlphaFold2 predict the impact of missense mutations on structure?. Nat. Struct. Mol. Biol..

[32] Pak M.A., Markhieva K.A., Novikova M.S., Petrov D.S., Vorobyev I.S., Maksimova E.S., Kondrashov F.A., Ivankov D.N. (2023). Using AlphaFold to predict the impact of single mutations on protein stability and function. PLoS One.

[33] Clapperton J.A., Manke I.A., Lowery D.M., Ho T., Haire L.F., Yaffe M.B., Smerdon S.J. (2004). Structure and mechanism of BRCA1 BRCT domain recognition of phosphorylated BACH1 with implications for cancer. Nat. Struct. Mol. Biol..

[34] Brzovic P.S., Rajagopal P., Hoyt D.W., King M.C., Klevit R.E. (2001). Structure of a BRCA1-BARD1 heterodimeric RING-RING complex. Nat. Struct. Biol..

[35] Abramson J., Adler J., Dunger J., Evans R., Green T., Pritzel A., Ronneberger O., Willmore L., Ballard A.J., Bambrick J. (2024). Accurate structure prediction of biomolecular interactions with AlphaFold 3. Nature.

