# Supplementary material for: ACMG/AMP interpretation of BRCA1 missense variants: Structure-informed scores add evidence strength granularity to the PP3/BP4 computational evidence
Source: Am J Hum Genet. 2025 Apr 14;112(5):993–1002. doi: 10.1016/j.ajhg.2024.12.011 (PMC12120176; doi:10.1016/j.ajhg.2024.12.011)
Supplement: Document S1. Figures S1–S15, Tables S2–S6, and supplemental methods [file mmc1.pdf]

The American Journal of Human Genetics, Volume *112*

Supplemental information

ACMG/AMP interpretation of *BRCA1* missense variants: Structure-informed scores add evidence strength granularity to the PP3/BP4 computational evidence

Lobna Ramadane-Morchadi, Nitsan Rotenberg, Ada Esteban-Sánchez, Cristina Fortuno, Alicia Gómez-Sanz, Matthew J. Varga, Adam Chamberlin, Marcy E. Richardson, Kyriaki Michailidou, Pedro Pérez-Segura, Amanda B. Spurdle, and Miguel de la Hoya

Supplemental Figures

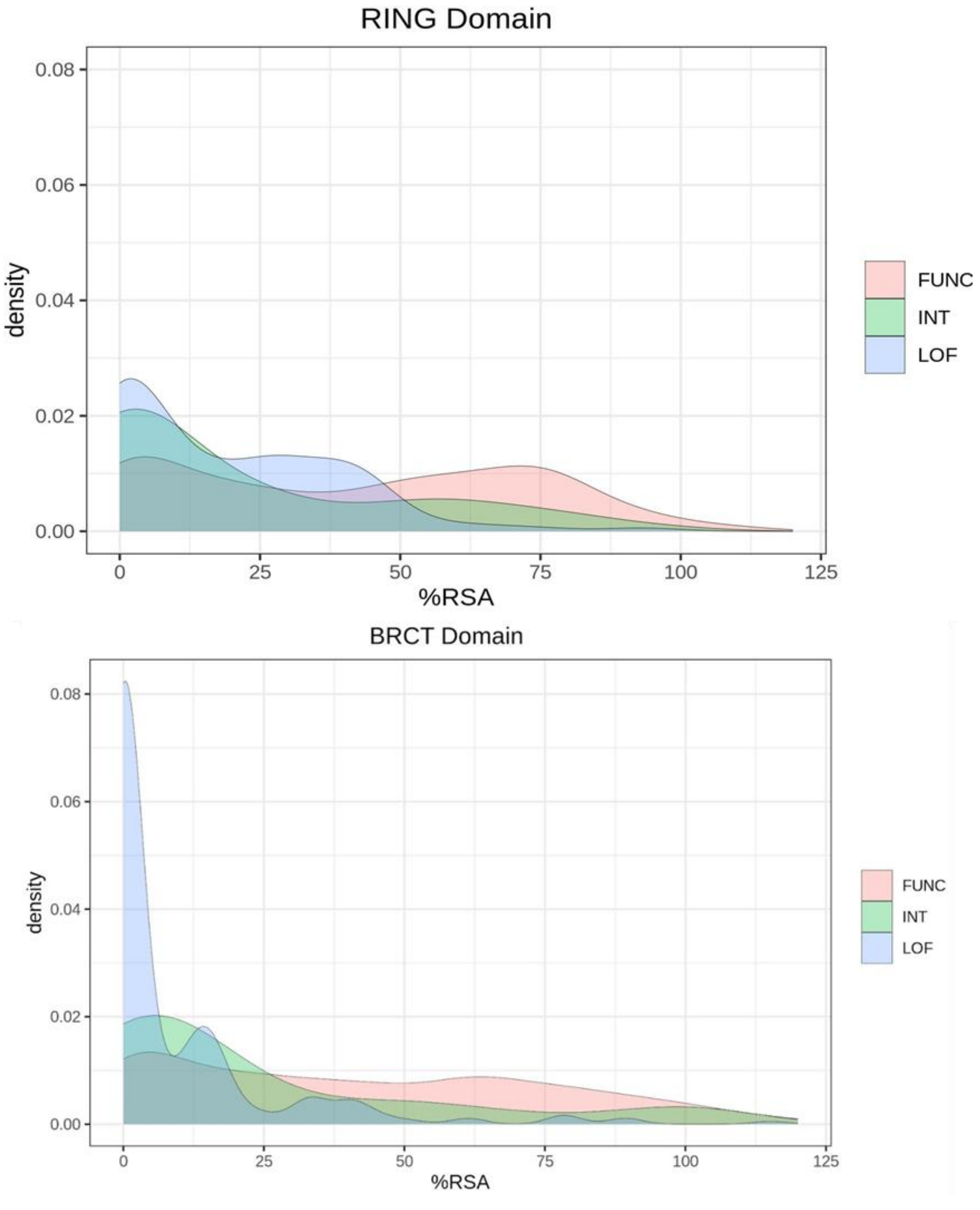

Figure S1A. The density plots show for the RING (top) and BRCT (bottom) domains, the distribution of residue solvent accessibility (%RSA) stratified by MAVE functional class. Residues targeted by functional (FUNC), intermediate (INT), and loss-of-function (LOF) variants are shown. Overall, INT and LOF variants tend to target buried residues. This trend is particularly striking for LOF variants targeting BRCT residues.

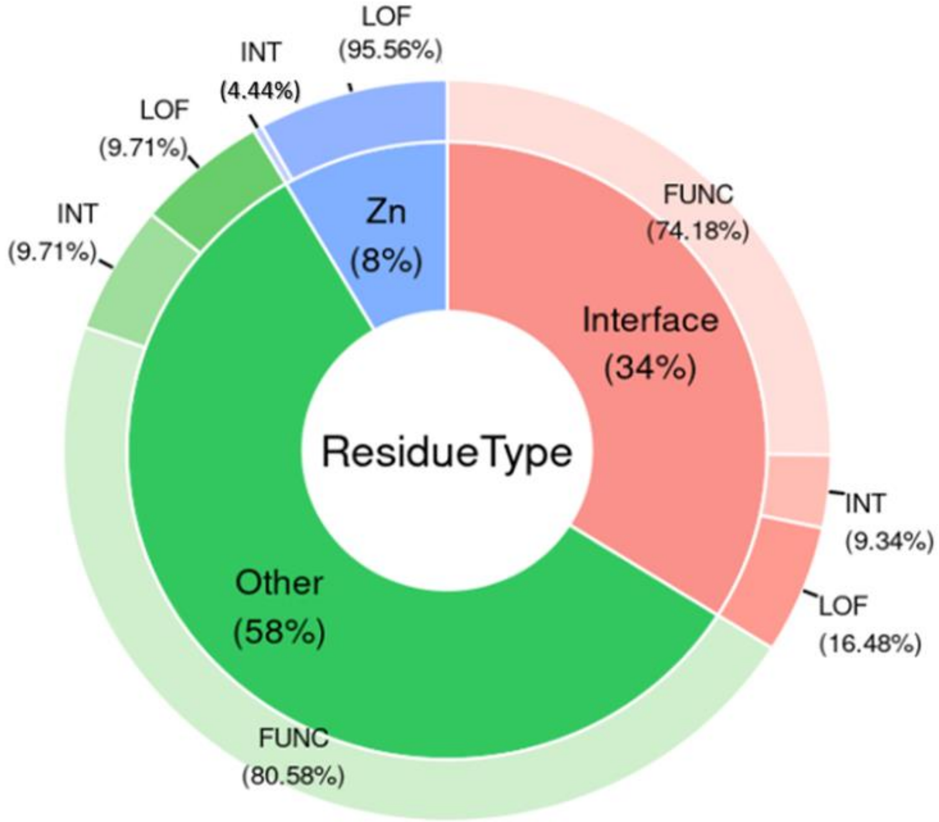

Figure S1B. The donut plot shows MAVE functional categories stratified by residue subtype in the BRCA1 RING domain. The inner circle shows RING residues manually stratified into the following subtypes: Zn-interacting, located in the interface with BARD1, and others. The external circle shows the proportion of MAVE functional categories per residue subtype. Essentially, all variants targeting Zn-interacting residues are LOF. The proportion of LOF variants targeting interface residues is higher than the proportion of LOF variants targeting other residues (16.5% vs. 9.7%). Interestingly, the proportion of INT variants targeting interface or other residues is similar.

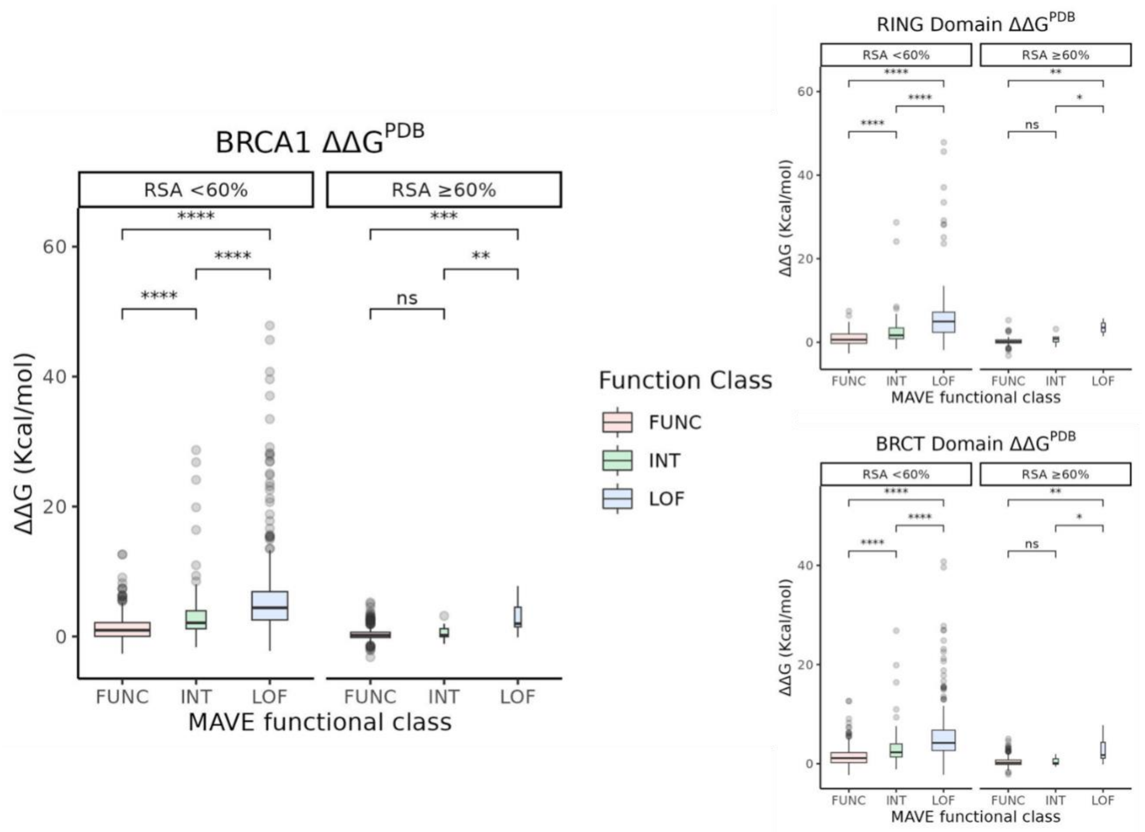

Figure S2. The boxplots show **$\Delta\Delta G^{PDB}$** distribution *per* MAVE functional class. Left panel shows stratification by RSA into buried-partially buried (RSA ≤60%) and exposed (RSA>60%) residues. Right panel shows additional stratification by functional domain into RING (top) and BRCT (bottom) variants.Box sizes are proportional to the number of variants (N) in each class. Upper and lower box hinges correspond to Q1 and Q3 (25% and 75% percentiles), while the upper and lower whiskers extends to +/- 1.5*IQR (interquartile range). Dots correspond to outlier values**.** Wilcoxon test p-values for pair comparisons are represented as: ns for p> 0.05 (non-significant),* p≤ 0.05, ** p≤ 0.01, *** p≤ 0.001 and **** p≤ 0.0001.

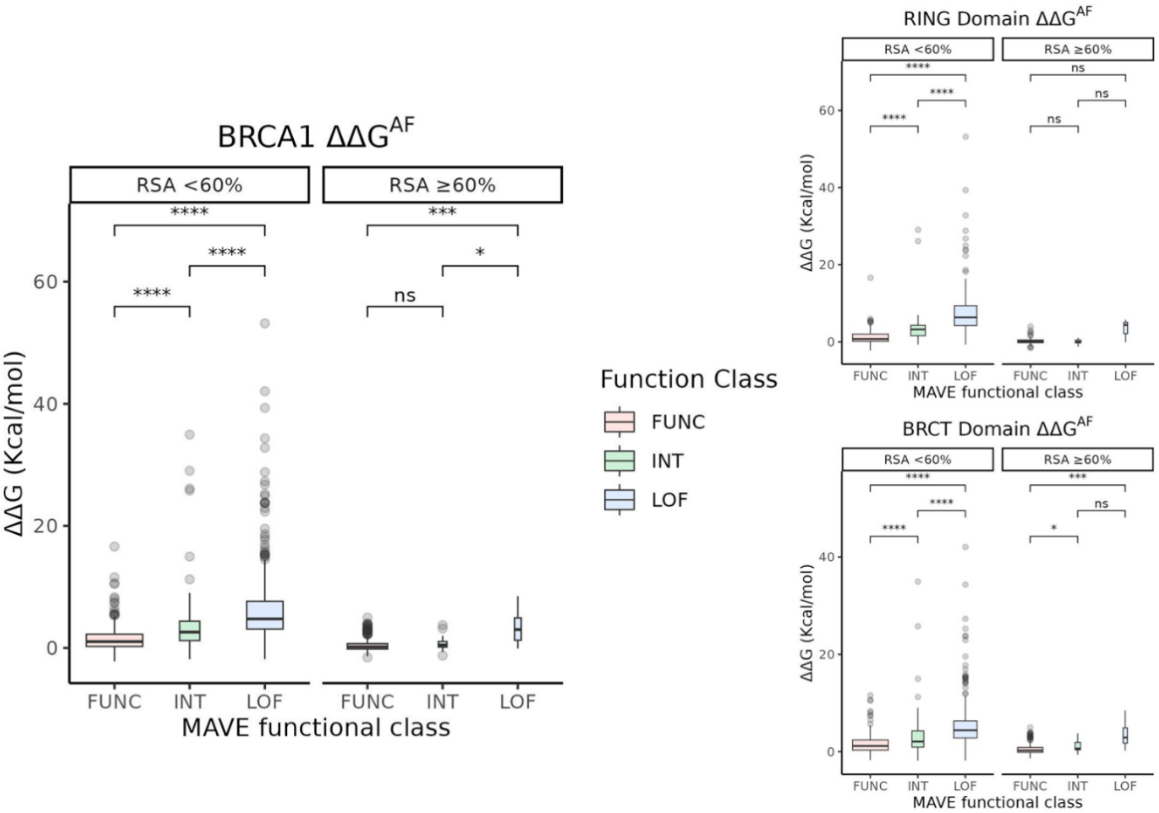

Figure S3. The boxplots show **$\Delta\Delta G^{AF}$** distribution *per* MAVE functional class. Left panel shows stratification by RSA into buried/partially buried (RSA ≤60%) and exposed (RSA>60%) residues. Right panel shows additional stratification by functional domain into RING (upper) and BRCT (bottom) variants.Box sizes are proportional to the number of variants (N) in each class. Upper and lower box hinges correspond to Q1 and Q3 (25% and 75% percentiles), while the upper and lower whiskers extends to +/- 1.5*IQR (interquartile range). Dots correspond to outlier values**.** Wilcoxon test p-values for pair comparisons are represented as: ns for $p > 0.05$ (non-significant),* $p \leq 0.05$, ** $p \leq 0.01$, *** $p \leq 0.001$ and **** $p \leq 0.0001$.

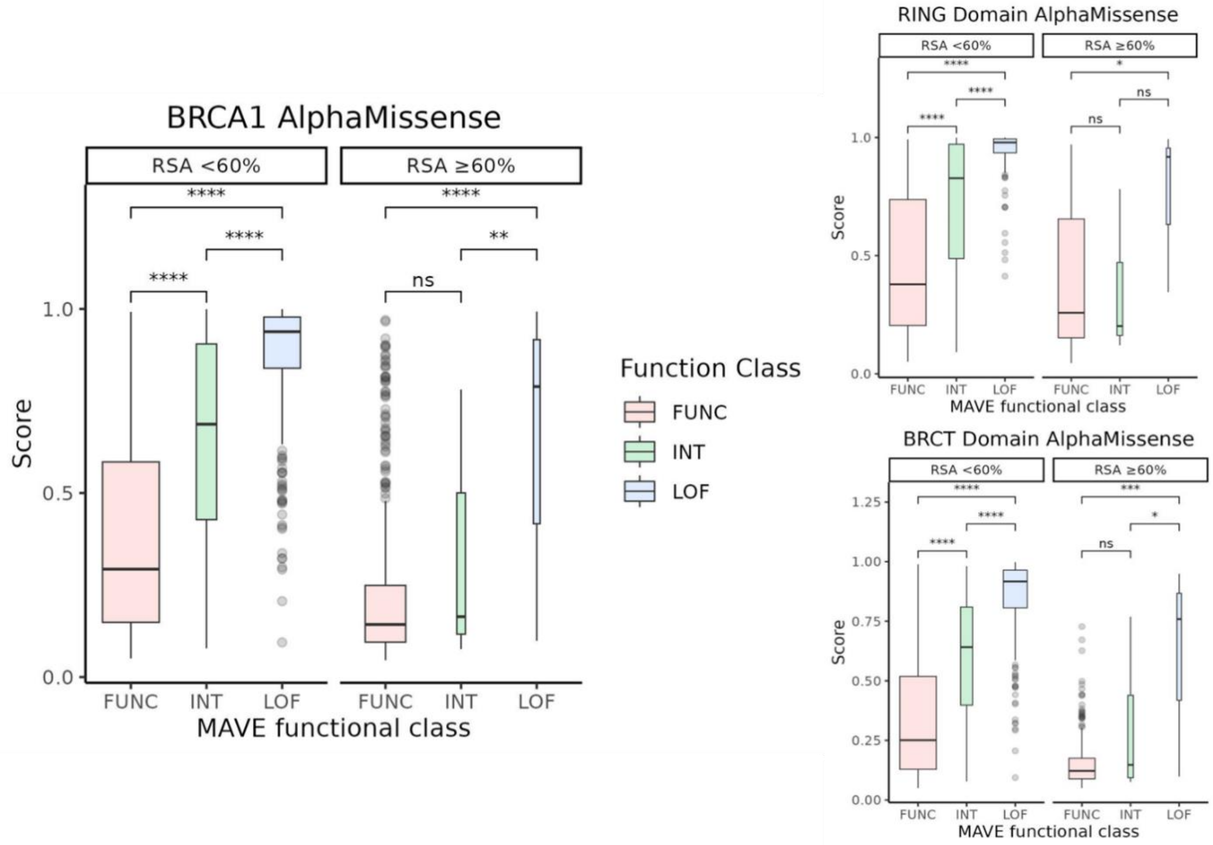

Figure S4. The boxplots show **AlphaMissense** pathogenicity score distribution *per* MAVE functional class. Left panel shows stratification by RSA into buried/partially buried (RSA ≤60%) and exposed (RSA>60%) residues. Right panel shows stratification by functional domain into RING (upper) and BRCT (bottom) variants.Box sizes are proportional to the number of variants (N) in each class. Upper and lower box hinges correspond to Q1 and Q3 (25% and 75% percentiles), while the upper and lower whiskers extends to +/- 1.5*IQR (interquartile range). Dots correspond to outlier values**.** Wilcoxon p-values for pair comparisons are represented as: ns for p> 0.05 (non-significant),* p≤ 0.05, ** p≤ 0.01, *** p≤ 0.001 and **** p≤ 0.0001.

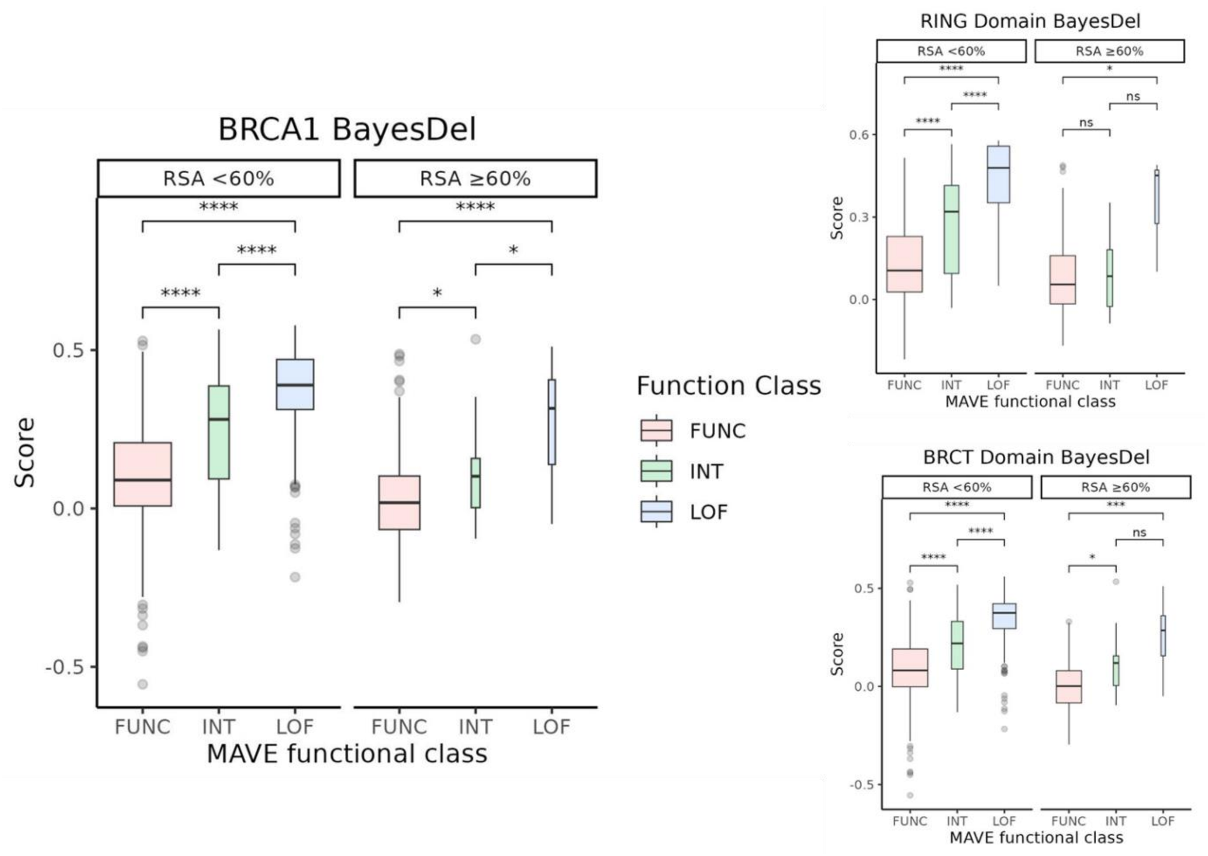

Figure S5. The boxplots show **BayesDel** score distribution *per* MAVE functional class. Left panel shows stratification by RSA into buried/partially buried (RSA ≤60%) and exposed (RSA>60%) residues. Right panel shows additional stratification by functional domain into RING (top) and BRCT (bottom) variants.Box sizes are proportional to the number of variants (N) in each class. Upper and lower box hinges correspond to Q1 and Q3 (25% and 75% percentiles), while the upper and lower whiskers extends to +/- 1.5*IQR (interquartile range). Dots correspond to outlier values**.** Wilcoxon p-values for pair comparisons are represented as: ns for p> 0.05 (non-significant),* p≤ 0.05, ** p≤ 0.01, *** p≤ 0.001 and **** p≤ 0.0001.

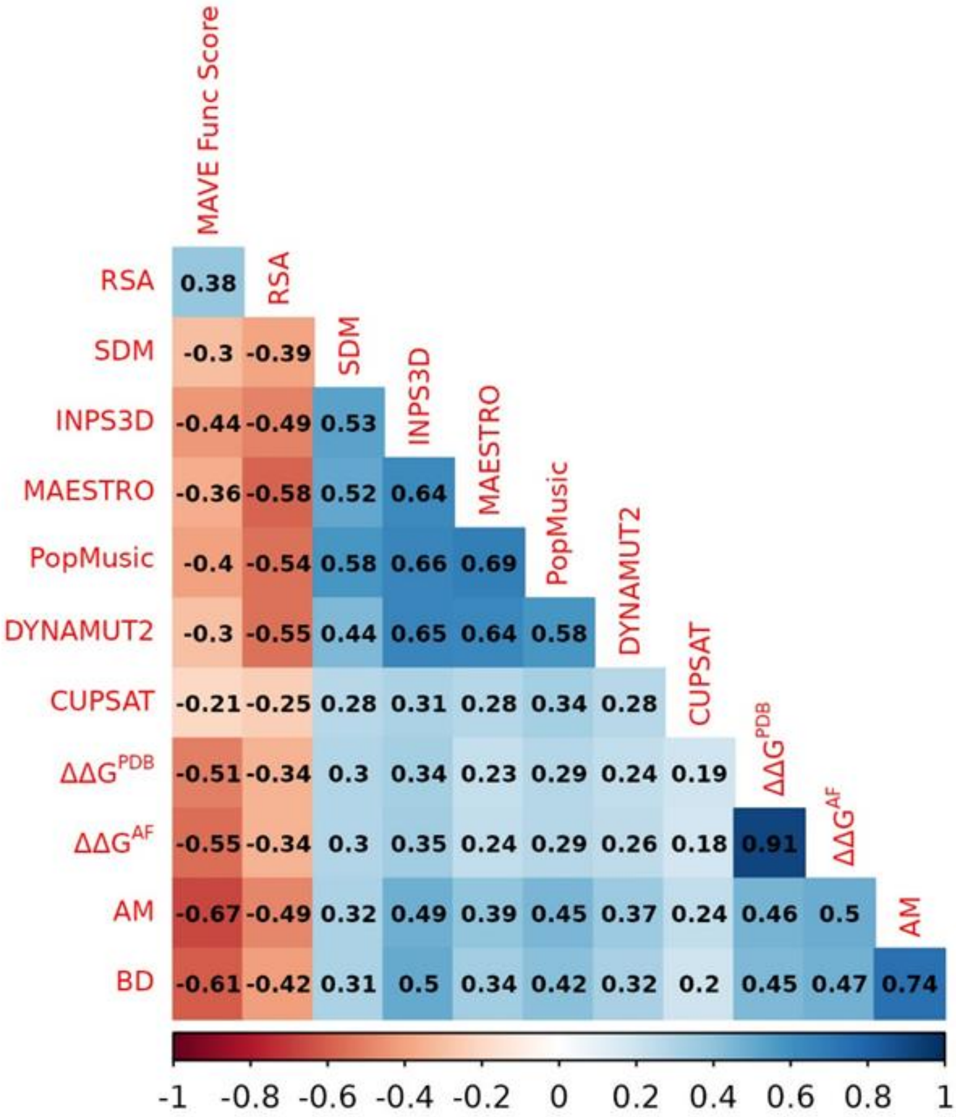

Figure S6. The plot summarizes the linear dependence (Pearson correlation coefficient **r**) between pairs of variables analysed in the present study. Note that correlation between FoldX5.0 ΔΔG predictions using experimental or AF2 templates (**$\Delta\Delta G^{PDB}$** vs. **$\Delta\Delta G^{AF}$)** is very high (r=0.91), but correlation between FoldX5.0 and web-based ΔΔG predictions is much lower (**r** ranging from 0.18 to 0.35). For the present correlation analysis, MAVE functional scores include INT variants. **RSA** (Residue solvent accessibility). **AM** (AlphaMissense). **BD** (BayesDel). **SDM, INPS3D, MAESTRO, PopMusic, DYNAMUT2, CUPSAT** (web-based ΔΔG predictors).

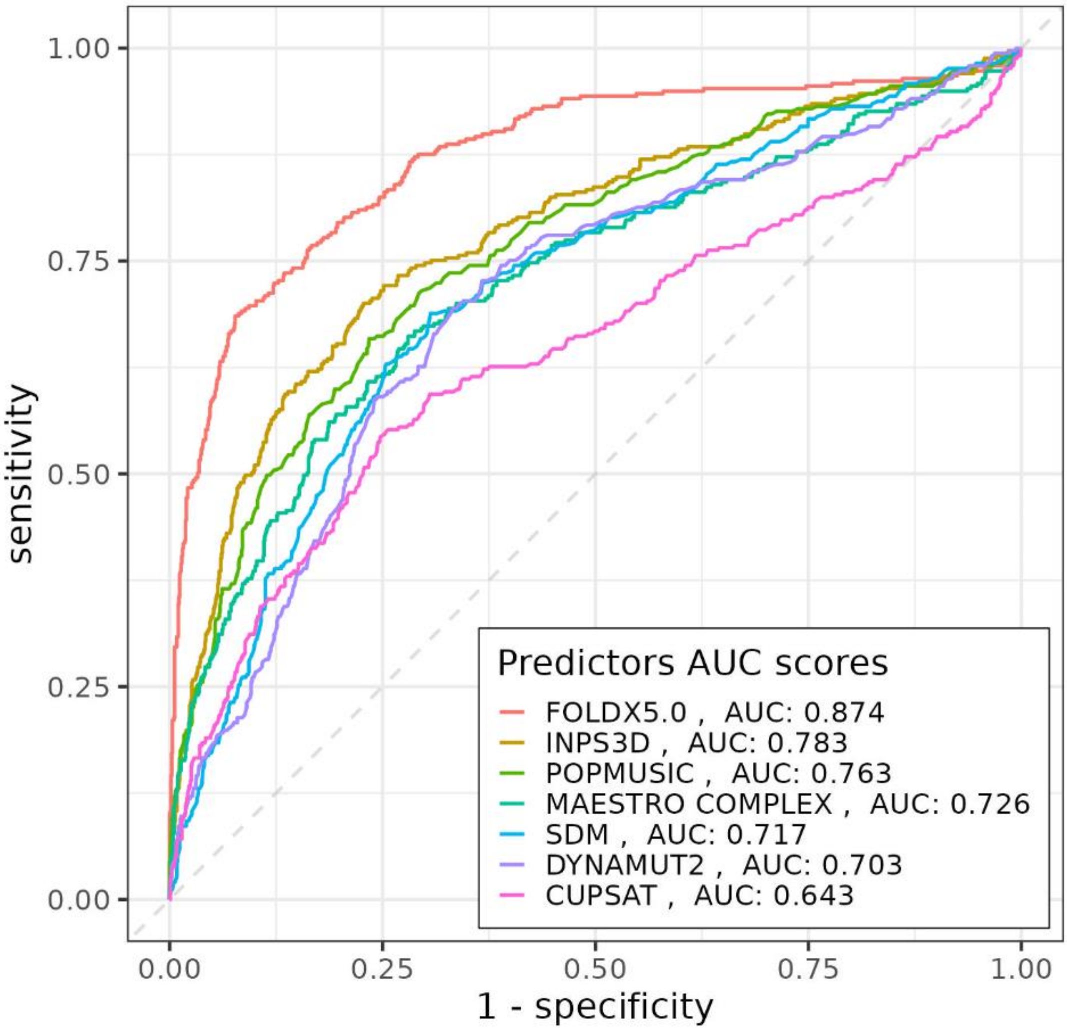

Figure S7. Analysis of FoldX5.0 $\Delta\Delta G^{PDB}$ and six web-based ΔΔG predictors (SDM, INPS3D, POPMUSIC, DYNAMUT, MAESTRO and CUPSAT) at discriminating LOF and FUNC variants at the RING and BRCT domains (MAVE dataset with INT variants filtered-out). For each predictor, a ROC plot and the corresponding auROC value are displayed. Overall, FoldX5.0 $\Delta\Delta G^{PDB}$ outperforms web-based programs. Delong's test shows a statistically significant difference between $\Delta\Delta G^{PDB}$ and INPS3D, the best web-based predictor (p-value = 3.35×10^{-10}). We performed an alternative analysis in which ΔΔG-stability was replaced by ΔΔG-interaction in the subset of variants targeting the BRCA1-BARD1 heterodimer interface, but no improvement on auROC was observed (data not shown).

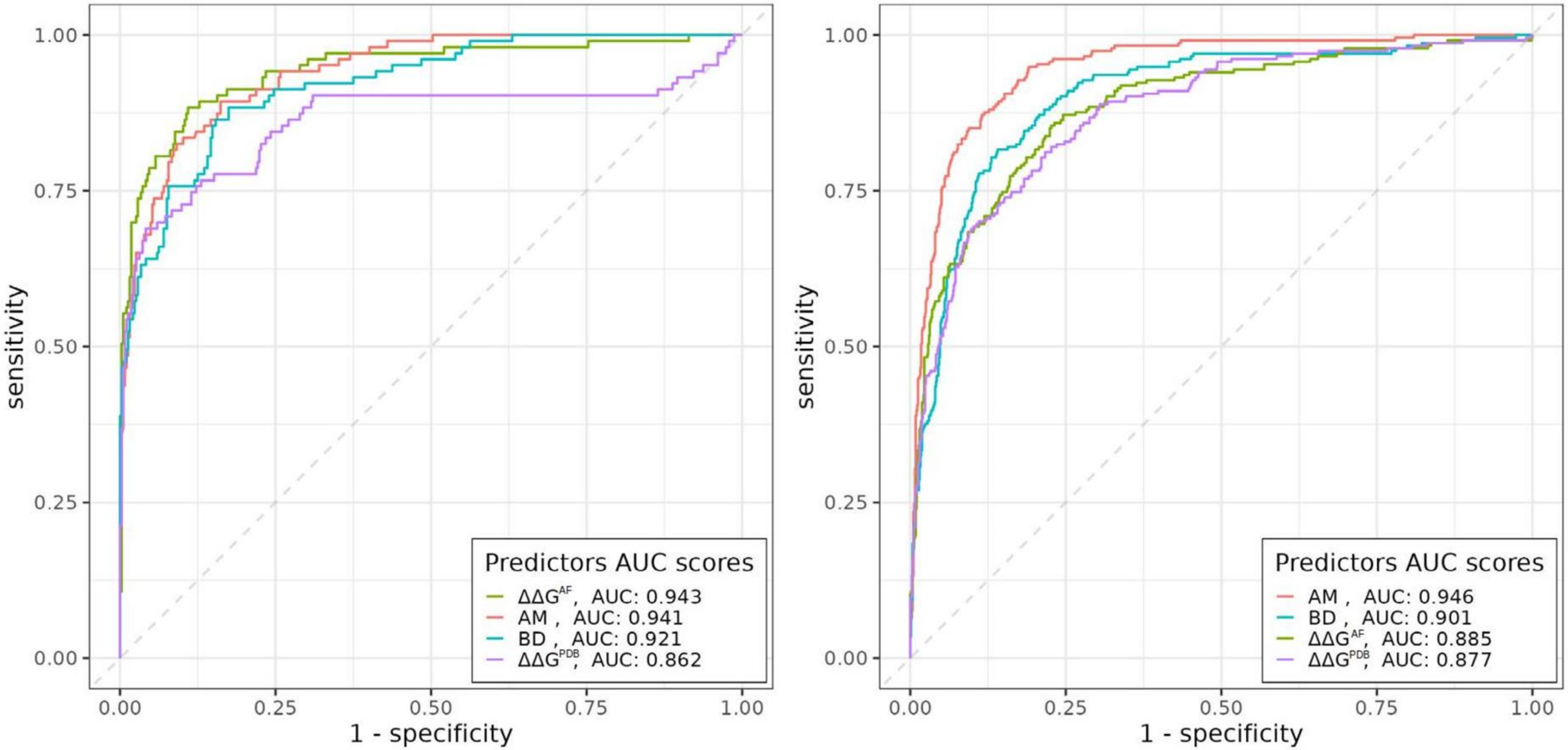

Figure S8. Analysis of AM**,** $\Delta\Delta G^{PDB}$, $\Delta\Delta G^{AF}$, and BayesDel performance at discriminating LOF and FUNC variants (MAVE dataset with INT variants filtered-out) at the RING (left panel) and BRCT domains (right panel). For each predictor, a ROC plot and the corresponding auROC value are displayed. Overall, $\Delta\Delta G^{AF}$ provides the best discrimination at the RING domain, while AM provides the best discrimination at the BRCT domain. Interestingly, $\Delta\Delta G^{AF}$ outperforms $\Delta\Delta G^{PDB}$, in particular at the RING domain (AUC 0.943 vs. 0.862). See text for further details.

	BD-based PP3/BP4			AM-based PP3/BP4			(AM+ΔΔG^{AF})-based PP3/BP4		
score	<=.15	><	>=.28	<=.65	><	>=.75	<=.65<=1.5	><	>=.75>=2.5
Log2 LR	-2.9	-0.5	+2.6	-2.9	**	+2.8	-4.5	-0.5	+4.2
N	883	216	420	1015	78	426	748	478	293
LR-based strength	BP4_M	n/a	PP3_M	BP4_M	n/a	PP3_M	BP4_S	n/a	PP3_S
Recommended strength	BP4	n/a	PP3	BP4	n/a	PP3	BP4_M	n/a	PP3_M

Figure S9. BayesDel, AlphaMissense, and (AlphaMissense+ΔΔG)-based PP3/BP4 computational evidence. Our study indicates that the BayesDel-based PP3/BP4 computational evidence (**BD-based PP3/BP4**) currently recommended by the *ClinGen ENIGMA BRCA1 and BRCA2 Variant Curation Expert Panel* (VCEP) for *BRCA1* missense variants at the RING and BRCA domains provides pathogenicity and benignity evidence with **LR-based strength** in the moderate to strong range (±2<Log2 LR<±4). This is in agreement with previous studies by the VCEP estimating at least moderate evidence for BayesDel. However, the VCEP opted, conservatively, to downgrade the evidence strength one level, applying the ACMG/AMP baseline supporting. Similarly, here we recommend downgrading evidence strength one level (**recommended strength**). An AlphaMissense-based PP3/BP4 computational evidence (**AM-based PP3/BP4**) is superior in that provides very similar evidence strengths to more variants (in our MAVE cohort of 337 LOF and 1182 FUNC variants, 216 variants have no PP3/BP4 evidence applicable with BD, while only 78 variants have no PP3/BP4 evidence applicable with AlphaMissense). A ΔΔG-based computational evidence performs very similar to BayesDel (**see Figure 2**). Concordant AlphaMissense and ΔΔG^{AF} scoring [**(AM+ ΔΔG^{AF})-based PP3/BP4**] provides BP4 and PP3 computational evidences with strong strengths (Log2 LRs -4.5 and +4.2, respectively), but the proportion of variants with no PP3/BP4 evidence strength applicable is higher (478 MAVE cohort variants have no PP3/BP4 evidence applicable with this combined approach). For clarity, ACMG/AMP Strong (S), moderate (M) and supporting (P) evidences are color-coded (green for benignity and brown for pathogenicity). (><) scoring in the uncertain zone between benignity and pathogenicity thresholds. (**) No statistically significant benign or pathogenic evidence (Log2 LR 95%CI includes 0). (n/a) not applicable. (**BD**) BayesDel. (**AM**) AlphaMissense. (**ΔΔG^{AF}**) FoldX5.0 ΔΔG predictions using AlphaFold2 PDB templates. **(><)** Bioinformatic code not applicable (**N**) MAVE dataset variants in each scoring category.

	(AM+$\Delta\Delta G^{AF}$)-based PP3/BP4					
**RSA score**	**<=60%**			**>60%**		
**N**	1109			410		
**AM + ΔΔG score**	**<=.65<=1.5**	**><**	**>=.75>=2.5**	**<=.65<1.5**	**><**	**>=.75>=2.5**
**Log2 LR**	-4.9	-0.7	+3.7	-1.6*	**	+7.5
**N**	426	396	287	322	82	6
**LR-based strength**	**BP4_S**	n/a	**PP3_M**	**BP4**	n/a	**PP3_S**
**Recommended strength**	**BP4_M**	n/a	**PP3**	n/a	n/a	**PP3_M**

Figure S10. Impact of RSA in (AlphaMissense+$\Delta\Delta G^{AF}$)-based PP3/BP4 computational evidence. In the subgroup of variants targeting exposed residues (RSA>60%), the approach did not provide statistically significant benignity evidence of supporting strength (**Log2 LR=-1.55, 95%CI -2.94 to -0.16**). For clarity, ACMG/AMP strong (S), moderate (M) and supporting (P) evidence strengths are color-coded green (benignity) and brown (pathogenicity). Following *ClinGen ENIGMA BRCA1 and BRCA2 Variant Curation Expert Panel* recommendations, we distinguish LR-based and recommended (conservative instance) strengths. (*****) No statistically significant benignity evidence (Log2 LR 95% CI overlaps -1). (******) No statistically significant benignity or pathogenicity evidence (Log2 LR 95% CI overlaps 0). **(RSA)** Residue Solvent Accessibility. **(AM)** AlphaMissense. **(><)** Bioinformatic code not applicable. **(N)** MAVE dataset variants in each score category.

(RSA/AM/$\Delta\Delta G^{AF}$)-based PP3/BP4

**RSA score**	**<=60%**							**>60%**						
**N**	**1109**							**410**						
**AM score**	**<=.65**			**><**	**>=.75**			**<=.65**			**><**	**>=.75**		
**Log2 LR**	-3.0				+2.5			-1.3*				+3.1		
**N**	649			66	394			366			12	32		
**ΔΔG score**	**<=1.5**	**><**	**>=3.5**		**<=1.5**	**><**	**>=3.5**	**<=0**	**><**	**>=2**		**<=0**	**><**	**>=2**
**Log2 LR**	-1.9		+2.4		-2.5		+1.7	-0.6**		+1.9**		-2.1**		+3.5
**N**	426	167	56	66	63	97	234	139	203	24	12	8	16	8
**Log2 LR + Log2 LR**	-4.9	-3.0	-0.6	n/a	0	+2.5	+4.2	-1.3*			n/a	+3.1		+6.6
**Evidence Strength:**														
**LR-based**	**BP4_S**	**BP4_M**	**n/a**			**PP3_M**	**PP3_S**	**n/a**				**PP3_M**		**PP3_S**
**Recommended**	**BP4_M**	**BP4**	**n/a**			**PP3**	**PP3_M**	**n/a**				**PP3**		**PP3_M**
**N**	426	167	185			97	234	378				24		8

Figure S11. Cascade stratification by RSA, AM and ΔΔG provide PP3/BP4 evidence strength granularity. We first stratified 1519 MAVE functional scores by **RSA** (60% cut-off) into variants targeting buried-exposed residues (N=1109) and variants targeting very exposed residues (N=410). We next stratified by **AM** score applying the indicated benignity (<=0.65) and pathogenicity (>=0.75) thresholds. Evidence strengths (Log2 LR) provided by **AM** are in the moderate to strong range (2<Log2 LR<4), except for benignity evidence in the very exposed subgroup (N=366), that reaches supporting strength only (Log2 LR=-1.3). We next stratified by **$\Delta\Delta G^{AF}$** (FoldX5.0 prediction) using different benignity and pathogenicity thresholds depending on **RSA**. In the (RSA<=60+AM<=0.65)-subgroup of variants (N=649), **$\Delta\Delta G^{AF}$** provides additional benignity evidence with near moderate strength (Log2 LR=-1.9) to 426 variants and, remarkably, pathogenicity evidence with strength in the moderate to strong range (Log2 LR=+2.4) to 56 variants. Similarly, **$\Delta\Delta G^{AF}$** provides benignity and pathogenicity evidences to the (RSA<=60+AM>=0.75)- and (RSA>60+AM>=0.75)-subgroups of variants. We next combine **AM** and **$\Delta\Delta G^{AF}$** evidence strengths (Lo2 LR + Log2 LR) in each subgroup of variants, and transform Log2 LR values into **ACMG/AMP** Strong (**S**), Moderate (**M**), Supporting (**P**), and no evidence strength (see methods). For clarity, benignity and pathogenicity evidence strengths are color-coded (green and brown palettes, respectively).(**N**) MAVE variants in each subgroup. (**n/a**) not applicable. (**><**) MAVE variants in the no evidence score range. (*****) Log2 LRs 95 CI overlaps -1. (******) Log2 LRs 95 CI overlaps zero.

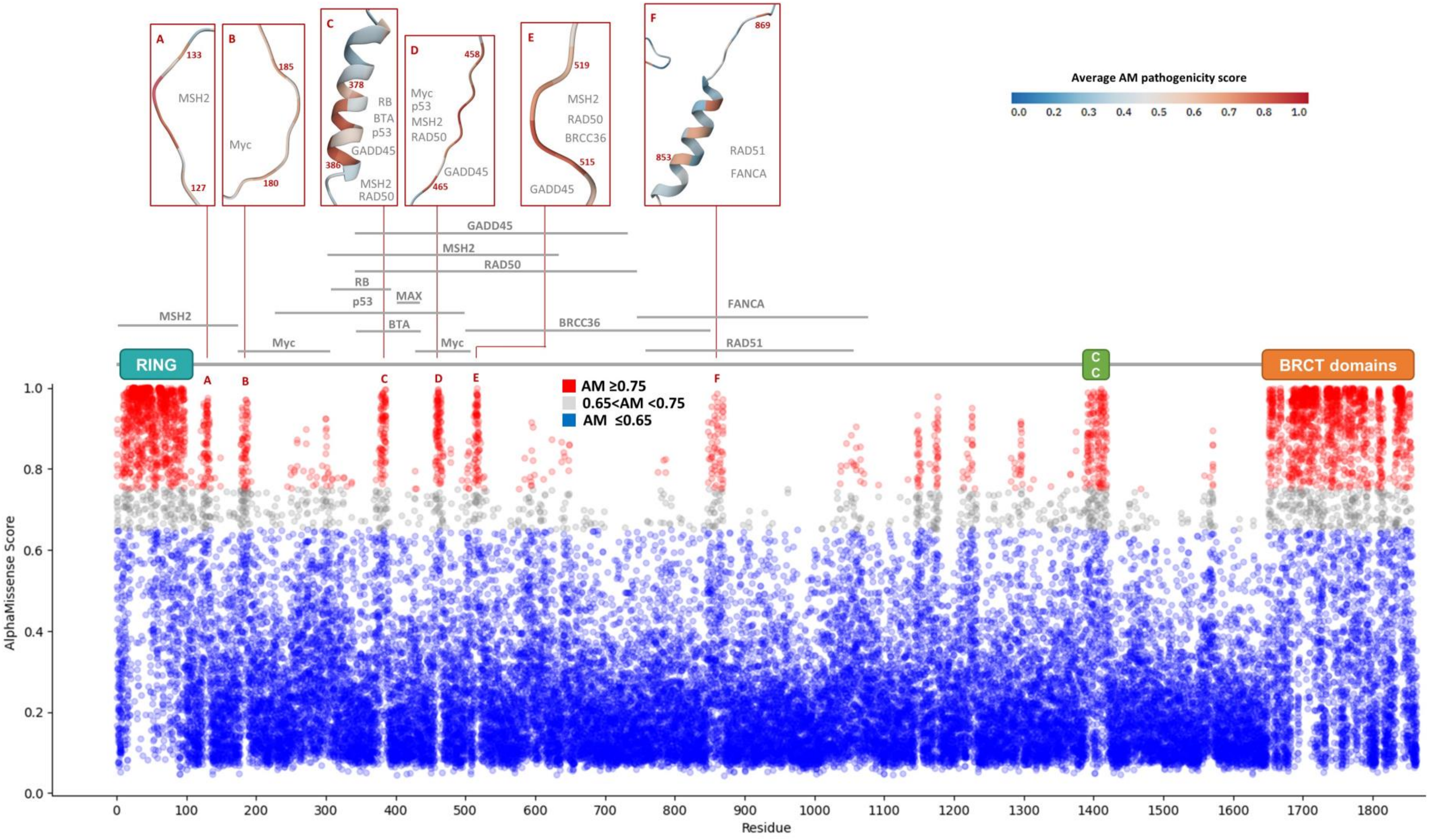

A
133
MSH2
127
B
185
Myc
180
C
378
RB
BTA
p53
GADD45
386
MSH2
RAD50
D
458
Myc
p53
MSH2
RAD50
GADD45
465
E
519
MSH2
RAD50
BRCC36
515
GADD45
F
869
853
RAD51
FANCA
Average AM pathogenicity score
0.0 0.2 0.3 0.4 0.5 0.6 0.7 0.8 1.0
GADD45
MSH2
RAD50
RB
p53
MAX
BTA
MSH2
Myc
Myc
BRCC36
FANCA
RAD51
RING
C C
BRCT domains
AM ≥0.75
0.65<AM <0.75
AM ≤0.65
AlphaMissense Score
Residue

Figure S12. The bottom plot (generated with Matplotlib) shows a graphical representation of AlphaMissense (AM) pathogenicity scores for each of the 19 possible substitutions at each of the 1863 BRCA1 residues. BRCA1-specific cut-offs for pathogenicity/benignity evidence are color-coded red/blue as indicated (no evidence color-coded grey). The plot shows hot AM clusters (pathogenicity scores enriched/benignity scores depleted) at the **RING** (residues 2-103), **CC** (residues 1391-1424), and **BRCT** (residues 1650-1857) domains, while the rest of the protein (mostly intrinsically disordered regions) show a very low rate of pathogenicity scores. Overall, this is in agreement with *expert panel* ACMG/AMP criteria specifications that apply PP3/BP4 computational evidence to missense variants targeting RING, CC and BRCT residues only, applying BP1_Strong to missense variants targeting other residues. Yet, the plot shows as well hot AM clusters spanning residues 127-133 (**A**), 180-185 (**B**), 378-386 (**C**), 458-465 (**D**), 515-519 (**E**) and 857-869 (**F**), suggesting that these BRCA1 regions might be relevant, and that BP1_Strong might not be warranted for missense variants targeting these hot AM clusters. Unfortunately, the scarcity of missense variants targeting the hot AM clusters in the BRIDGES dataset did not allow us to perform any clinical validation. As far as we know, no high quality BRCA1 functional annotation maps to any of these regions (Uniprot P38398, feature viewer, last access 22/08/2024). However, we hypothesize that these hot AM clusters might be functionally and clinically relevant, as they overlap BRCA1 regions with *in vitro* data supporting binding to partners proteins (1–4). Data is summarized in the central part of the figure. Cluster A (disordered region) overlaps a **MSH2** binding region (residues 1-175). Cluster B (disordered region) overlaps a **Myc** binding region (175-303). Cluster C (α–helix) overlaps **p53** (224-500), **Rb** (304-394), **RAD50** (341-758), **MSH2** (303-625), and **GADD45** (341-748) binding regions, as well as the BRCA1 basal transcriptional activation (**BTA**) region (4). Cluster D (disordered region) overlaps **Myc** (433-511), **p53** (224-500), **RAD50** (341-758), **MSH2** (303-625), and **GADD45** (341-748) binding regions. Cluster E (disordered region) overlaps **BRCC36** (502-852), **RAD50** (341-758), **MSH2** (303-625), and **GADD45** (341-748) binding regions. Finally, Cluster F (α–helix plus disordered region) overlaps **RAD51** (758-1064) and **FANCA** (740-1083) regions. For clarity, we have indicated BRCA1 interactions potentially disturbed by missense changes at the hot AM clusters in the corresponding top panels.

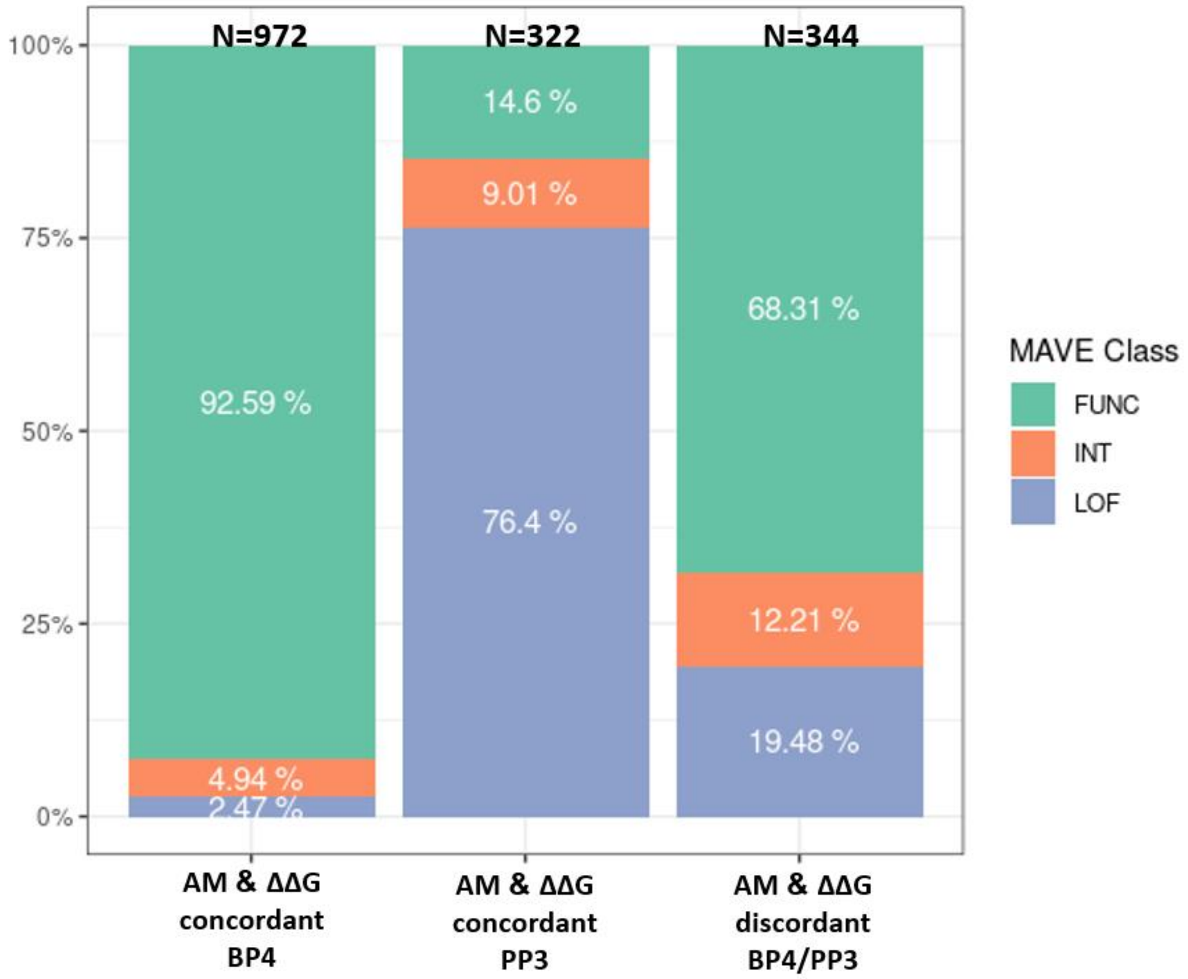

Figure S13. The bar plot shows the distribution of MAVE functional classes in the subgroup of variants (N=972) with concordant AlphaMissense- and $\Delta\Delta G^{AF}$-based BP4 evidence (left), in the subgroup of variants (N=322) with concordant AlphaMissense- and $\Delta\Delta G^{AF}$-based PP3 evidence (center), and in the subgroup of variants (N=344) with discordant AlphaMissense- and $\Delta\Delta G^{AF}$-based computational evidence. Interestingly, the higher proportion of INT variants (12.2%) is observed in the subgroup of variants with AM and $\Delta\Delta G^{AF}$ discordance. For the purpose of this analysis, concordant AM- and $\Delta\Delta G^{AF}$-based BP4 evidence if AM<0.75 and $\Delta\Delta G^{AF}$ <2.5, concordant AM- and $\Delta\Delta G^{AF}$-based PP3 evidence if AM ≥0.75 and $\Delta\Delta G^{AF}$ ≥2.5), and discordant AM- and $\Delta\Delta G^{AF}$ if AM<0.75 and $\Delta\Delta G^{AF}$ ≥2.5 (or AM ≥0.75 and $\Delta\Delta G^{AF}$ <2.5).

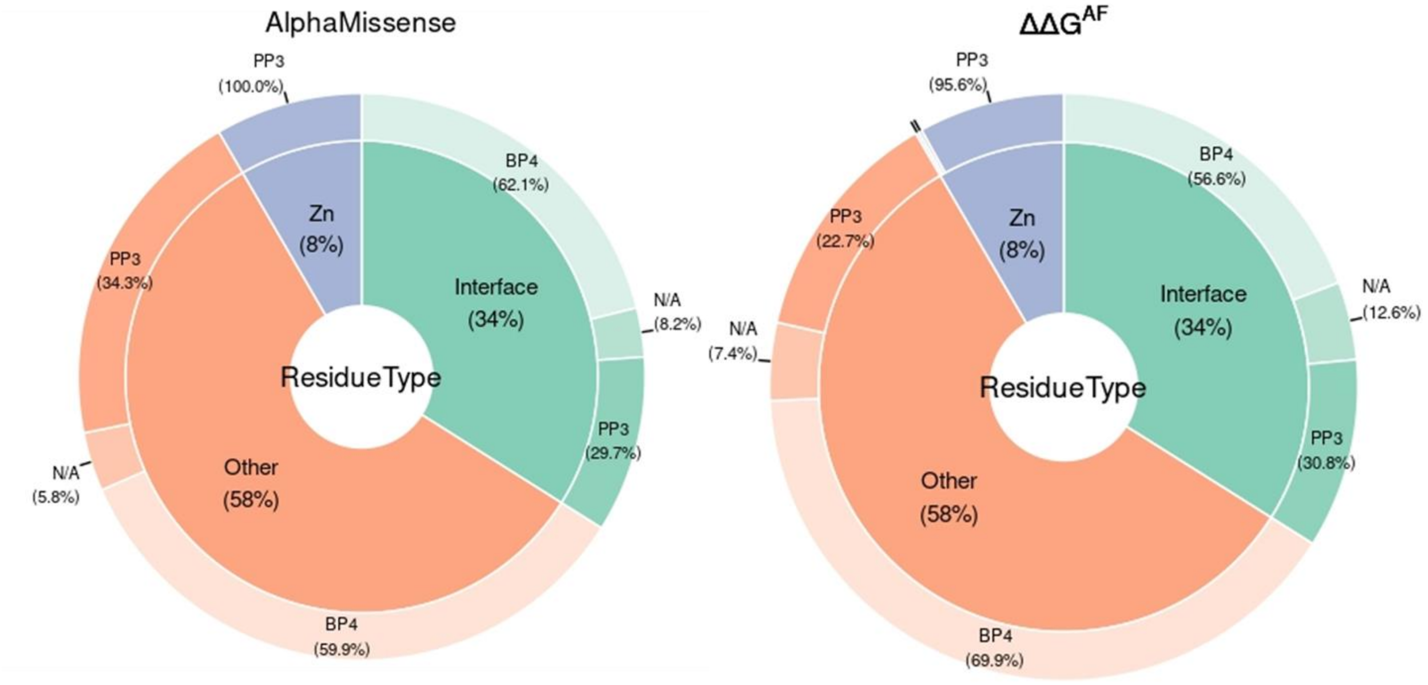

Figure S14. The inner circle of the donut plots show BRCA1 RING residues manually stratified into three residue subtypes: Zn-interacting, BARD1 interface, and others. The external circle (left) shows the proportion of AlphaMissense-based PP3, BP4, and no computational code applicable (N/A) missense variants *per* subtype. The external circle (right) shows the proportion of $\Delta\Delta G^{AF}$-based PP3, BP4, and no computational code applicable (N/A) missense variants *per* class. Note that no major differences between AlphaMissense and $\Delta\Delta G^{AF}$ are observed in the subtype of variants targeting Zn-interacting residues (essentially, all variants score PP3), or BARD1 interacting residues. By contrast, AlphaMissense provides a higher proportion of PP3 variants (34% vs. 23%) in the subtype of variants targeting "other residues". The data suggest that most BRCA1 missense variants targeting Zn- or BARD1-interacting residues are pathogenic via impact on protein stability, while a significant proportion of missense variants targeting "other residues" might be pathogenic via mechanisms other than destabilization.

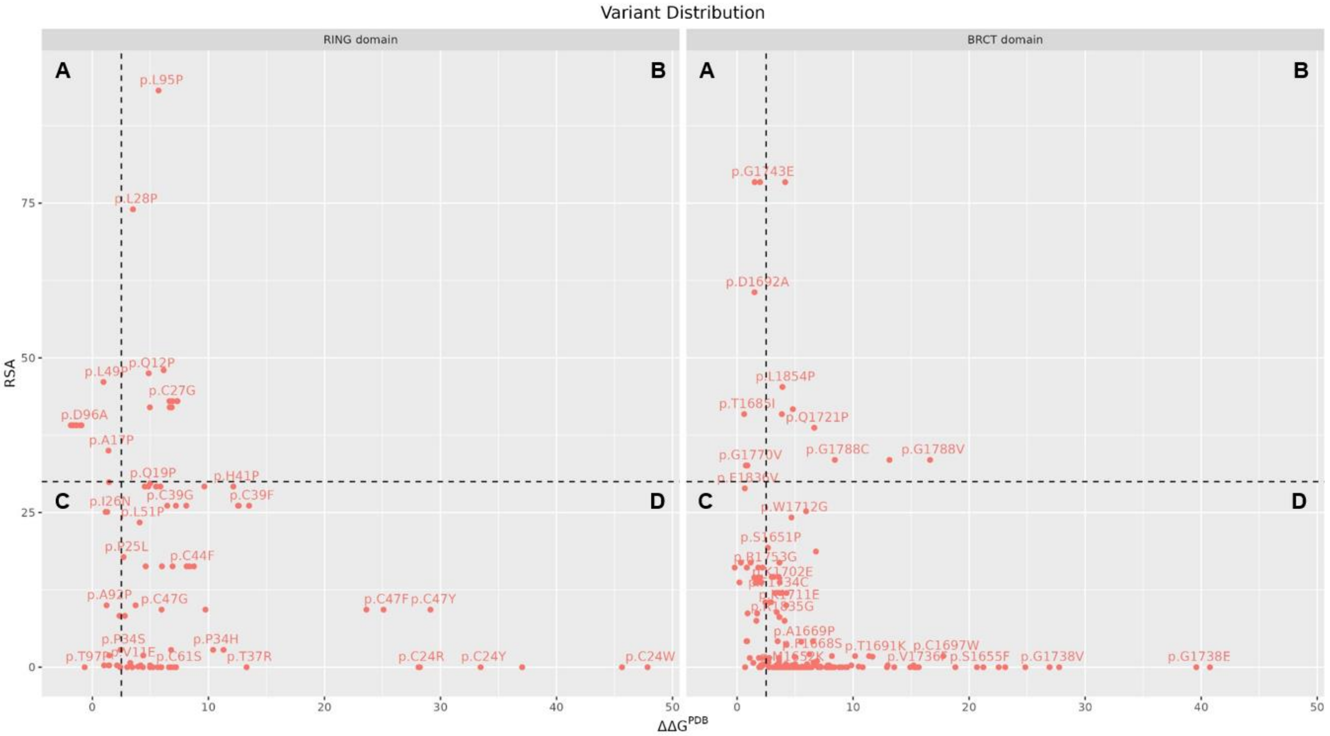

Figure S15. All displayed variants score LOF (MAVE) and PP3 (AlphaMissense). For the purpose of this example, we will refer to pathogenic variants. The left (RING domain) and right (BRCT domain) plots show BRCA1 missense variants (91 RING and 188 BRCT) distributed by RSA and $\Delta\Delta G^{PDB}$ (FoldX5.0 prediction). Dashed lines set at 30% RSA and ΔΔG +2.5 Kcal/mol define four plot sectors named A (non-destabilizing pathogenic variants targeting exposed residues), B (destabilizing pathogenic variants targeting exposed residues), C (non-destabilizing pathogenic variants targeting buried residues), and D (destabilizing pathogenic variants targeting buried residues). Most RING (64%) and BRCT (77%) pathogenic variants cluster in D, supporting that reduced thermodynamic stability is a major driver of pathogenicity for BRCA1 missense variants at the RING and BRCT domains. As expected, non-destabilizing pathogenic variants tend to target exposed residues (45% of A+B variants vs. 16% of C+D variants). A+C variants target residues that probably have relevant functions other than contributing to stabilization of the folded domain. For instance, A variants (partially exposed) might target surface residues involved in interactions with relevant protein partners, and C variants (buried) might be relevant for intermediate steps of the folding process.

Supplemental Tables

Table S1. Structure-informed scores for 1638 *BRCA1* missense variants with functional data (see accompanying Excel file)

Location of target residues	MAVE functional class	N	MAVE functional score	RSA (%)	$\Delta\Delta G^{PDB}$ (kcal/mol)	$\Delta\Delta G^{AF}$ (kcal/mol)	AM	BD
Domain stratification								
**RING**	LOF	103	-2.29	18.27	7.09	8.51	0.93	0.43
	INT	49	-1.01	21.16	3.00	3.54	0.67	0.25
	FUNC	384	-0.16	41.65	0.64	0.85	0.44	0.12
**BRCT**	LOF	234	-2.06	9.42	5.77	5.69	0.84	0.34
	INT	70	-1.02	24.41	3.16	3.31	0.55	0.20
	FUNC	798	-0.07	41.81	1.08	1.13	0.28	0.06
RSA stratification								
**Buried** (RSA<30%)	LOF	286	-2.11	5.53	6.61	6.74	0.88	0.37
	INT	87	-1.03	6.83	3.99	4.22	0.69	0.26
	FUNC	506	-0.15	9.81	1.54	1.70	0.44	0.13
**Partially Buried** (30%≤RSA≤60%)	LOF	40	-2.17	40.56	3.93	6.09	0.81	0.39
	INT	14	-0.98	47.76	0.82	1.72	0.44	0.08
	FUNC	277	-0.09	45.30	0.70	0.79	0.27	0.07
**Exposed** (RSA>60%)	LOF	11	-1.97	80.20	2.93	3.36	0.66	0.28
	INT	18	-0.96	82.39	0.56	0.77	0.30	0.11
	FUNC	399	-0.05	79.81	0.35	0.38	0.23	0.03

Table S2 displays relevant MAVE dataset average scores stratified by functional domain (RING vs. BRCT), or residue solvent accessibility (**RSA**). MAVE functional categories and functional scores as reported in the original publication (see methods). **$\Delta\Delta G^{PDB}$** (FoldX5.0 ΔΔG predictions using experimental PDBs as input templates). **$\Delta\Delta G^{AF}$** (FoldX5.0 ΔΔG predictions using AlphaFold2 models as input templates). **AM** (AlphaMissense pathogenicity scores). **BD** (BayesDel scores).

Tool	BRCA1 domain	**Benignity evidence (BP4)**		**Bioinformatic code not applicable** (%)	**Pathogenicity evidence** (**PP3**)	
		threshold	Evidence strength Log2 LR (95% CI)		threshold	strength Log2 LR (95% CI)
**AM**	RING	<=0.65	**-3.599** (-4.723 to -2.476)	6%	>=0.75	**+2.043** (+1.760 to +2.328)
	BRCT		**-2.741** (-3.216 to -2.267)	5%		**+3.507** (+3.135 to +3.878)
**$\Delta\Delta G^{AF}$**	RING	<=1.5	**-3.312** (-4.274 to -2.348)	8%	>=2.50	**+2.778** (+2.393 to +3.163)
	BRCT		**-2.789** (-3.353 to -2.225)	14%		**+2.187** (+1.947 to +2.428)
**BD**	RING	<=0.15	**-3.038** (-4.004 to -2.018)	17%	>=0.28	**+2.397** (+2.053 to +2.741)
	BRCT		**-2.888** (-3.438 to -2.339)	13%		**+2.802** (+2.500 to +3.104)

Table S3. The table shows the performance of **AM** (AlphaMissense), **$\Delta\Delta G^{AF}$** (FoldX5.0 predictions), and **BD** (BayesDel)-based PP3/BP4 computational evidence stratified by BRCA1 domain (RING vs. BRCT). We use the MAVE dataset (INT variants filtered-out) as proxy for pathogenicity and benignity. For **AM** and **$\Delta\Delta G^{AF}$,** we use optimal cut-off thresholds (see Table 1 and Figure 2). For **BD**, we used cut-off thresholds defined by the ClinGen ENIGMA *BRCA1* and *BRCA2* variant curation expert panel. Regardless of the BRCA1 functional domain, the computational tools provide benignity and pathogenicity evidence strengths in the moderate to strong range. AM outperforms other computational tools in providing similar evidence strengths with a lower proportion of variants in the BP4/PP3 not applicable range.

<table>
<tr><th></th><th>threshold</th><th>BRCA1 Domain</th><th>unique missense variants</th><th>BC</th><th>controls</th><th>OR (95% CI)</th><th>p</th></tr>
<tr><td rowspan="5">AM</td><td rowspan="2">>=0.75</td><td>RING</td><td>12</td><td>61</td><td>14</td><td>3.9 (2.2-7.0)</td><td>$5.5x10^{-6}$</td></tr>
<tr><td>BRCT</td><td>21</td><td>33</td><td>4</td><td>7.4 (2.4-20.9)</td><td>$1.7x10^{-4}$</td></tr>
<tr><td colspan="7"></td></tr>
<tr><td rowspan="2"><=0.65</td><td>RING</td><td>24</td><td>33</td><td>23</td><td>1.3 (0.8-2.2)</td><td>n.s.</td></tr>
<tr><td>BRCT</td><td>58</td><td>113</td><td>78</td><td>1.3 (1.0-1.7)</td><td>n.s.</td></tr>
<tr><td colspan="8"></td></tr>
<tr><td rowspan="5">$\Delta\Delta G^{AF}$</td><td rowspan="2">>=+2.5</td><td>RING</td><td>10</td><td>62</td><td>11</td><td>5.1 (2.7-9.6)</td><td>$1.1x10^{-6}$</td></tr>
<tr><td>BRCT</td><td>22</td><td>35</td><td>9</td><td>3.5 (1.8-7.3)</td><td>$8.7x10^{-4}$</td></tr>
<tr><td colspan="7"></td></tr>
<tr><td rowspan="2"><=+1.5</td><td>RING</td><td>20</td><td>26</td><td>18</td><td>1.3 (0.7-2.4)</td><td>n.s.</td></tr>
<tr><td>BRCT</td><td>47</td><td>96</td><td>73</td><td>1.2 (0.9-1.6)</td><td>n.s.</td></tr>
<tr><td colspan="8"></td></tr>
<tr><td rowspan="5">$\Delta\Delta G^{PDB}$</td><td rowspan="2">>=+2.5</td><td>RING</td><td>10</td><td>59</td><td>13</td><td>4.1 (2.2-7.4)</td><td>$6.1x10^{-6}$</td></tr>
<tr><td>BRCT</td><td>25</td><td>62</td><td>17</td><td>3.3 (1.9-5.6)</td><td>$1.8x10^{-5}$</td></tr>
<tr><td colspan="7"></td></tr>
<tr><td rowspan="2"><=+1.5</td><td>RING</td><td>24</td><td>32</td><td>23</td><td>1.3 (0.7-2.1)</td><td>n.s.</td></tr>
<tr><td>BRCT</td><td>44</td><td>93</td><td>67</td><td>1.2 (0.9-1.1)</td><td>n.s.</td></tr>
<tr><td colspan="8"></td></tr>
<tr><td rowspan="5">BD</td><td rowspan="2">>=0.28</td><td>RING</td><td>7</td><td>58</td><td>9</td><td>5.8 (2.9-11.7)</td><td>$1.4x10^{-6}$</td></tr>
<tr><td>BRCT</td><td>26</td><td>62</td><td>21</td><td>2.6 (1.6-4.3)</td><td>$1.3x10^{-4}$</td></tr>
<tr><td colspan="7"></td></tr>
<tr><td rowspan="2"><=0.15</td><td>RING</td><td>21</td><td>16</td><td>17</td><td>0.8 (0.4-1.7)</td><td>n.s.</td></tr>
<tr><td>BRCT</td><td>49</td><td>72</td><td>58</td><td>1.1 (0.8-1.6)</td><td>n.s.</td></tr>
</table>

Table S4. BRIDGES-based Breast Cancer risk estimates stratified by AM, $\Delta\Delta G^{AF}$, and BD scoring. The BRIDGES-based burden analysis (population-based only, 53,572 **BC** cases and 48,048 matched **controls**, see methods for details) demonstrates that, on average, RING or BRCT missense variants scoring AM≥0.75, $\Delta\Delta G^{AF}$≥2.5, $\Delta\Delta G^{PDB}$≥2.5, or BD≥0.28 are risk associated, supporting PP3 evidence. Similarly, the study supports BP4 for RING or BRCT missense variants scoring AM ≤0.65, $\Delta\Delta G^{AF}$≤1.5, $\Delta\Delta G^{PDB}$≤1.5, or BD ≤0.15. AM (AlphaMissense). (n.s.) no statistically significant

Tool	Benignity evidence (**BP4**) Log2 LR (95% CI)	RSA	Pathogenicity evidence (**PP3**) Log2 LR (95% CI)
AM	**-3.11** (-3.66 to-2.55)	<=30%	**+2.10** (+1.85 to +2.36)
	**-2.00** (-2.84 to-1.17)	30-60%	**+3.89** (+3.11 to +4.67)
	-1.32 (-2.45 to -0.19)*	>60%	**+3.07** (+2.12 to +4.01)
$\Delta\Delta G^{AF}$	**-2.62** (-3.18 to -2.05)	<=30%	**+1.52** (+1.31 to +1.74)
	**-3.02** (-4.36 to -1.68)	30-60%	**+3.35** (+2.76 to +3.95)
	-1.69 (-3.09 to -0.30)*	>60%	**+3.67** (+2.77 to +4.56)
$\Delta\Delta G^{PDB}$	**-2.24** (-2.73 to -1.76)	<=30%	**+1.57** (+1.34 to +1.80)
	**-2.11** (-2.50 to -1.73)	30-60%	**+2.51** (+1.87 to +3.95)
	-1.73 (-3.12 to -0.34)*	>60%	**+3.41** (+2.27 to +3.14)
BD	**-2.83** (-3.39 to -2.27)	<=30%	**+1.93** (+1.67 to +2.18)
	**-2.61** (-3.80 to -1.43)	30-60%	**+3.16** (+2.56 to +3.76)
	-1.63 (-3.02 to -0.24)*	>60%	**+3.52** (+2.56 to +4.52)

Table S5. The table shows the performance of **AM** (AlphaMissense)-, $\Delta\Delta\mathbf{G}^{\mathbf{AF}}$ (FoldX5.0 predictions)-, $\Delta\Delta\mathbf{G}^{\mathbf{PDB}}$ (FoldX5.0 predictions)-, and **BD** (BayesDel)-based PP3/BP4 computational evidence stratified by RSA. We use the MAVE dataset (INT variants filtered-out) as proxy for pathogenicity and benignity. For **AM**, $\Delta\Delta\mathbf{G}^{\mathbf{AF}}$**,** and $\Delta\Delta\mathbf{G}^{\mathbf{PDB}}$ we use optimal cut-off thresholds (see Table 1 and Figure 2). For BD, we use cut-off thresholds defined by the ClinGen ENIGMA BRCA1 and BRCA2 variant curation expert panel. Note that no bioinformatic tool provides statistically significant benignity evidence of supporting strength for missense variants targeting exposed (RSA>60%) residues (in all cases the Log2 LR 95% CI overlaps -1).

	RSA	Benignity evidence (**BP4**) Log2 LR (95% CI)	Tool	Pathogenicity evidence (**PP3**) Log2 LR (95% CI)
RING	≤60% (N=354)	**-3.79** (-5.03 to -2.55)	AM	**+1.99** (+1.65 to +2.31)
		**-3.27** (-4.31 to -2.23)	$\Delta\Delta G^{AF}$	**+2.28** (+1.90 to +2.66)
		**-3.10** (-4.15 to -2.06)	BD	**+2.23** (+1.84 to +2.62)
	>60% (N=134)	-1.35 (-3.76 to +1.05)*	AM	+1.52 (+0.12 to +2.92)**
		-1.73 (-4.12 to +0.65)*	$\Delta\Delta G^{AF}$	**+4.63** (+2.58 to +6.71)
		-1.30 (-3.70 to +1.09)*	BD	+2.22 (+0.75 to +3.68)**
BRCT	≤60% (N=755)	**-2.75** (-3.25 to -2.24)	AM	**+2.93** (+2.57 to +3.30)
		**-2.67** (-3.27 to -2.08)	$\Delta\Delta G^{AF}$	**+1.80** (+1.55 to +2.04)
		**-2.81** (-3.39 to -2.23)	BD	**+2.28** (+1.98 to +2.58)
	>60% (N=227)	-1.49 (-2.80 to -0.18)*	AM	**+8.16** (+4.05 to +12.25)
		-1.76 (-3.49 to -0.03)*	$\Delta\Delta G^{AF}$	**+3.31** (+2.28 to +4.32)
		-1.86 (-3.59 to -0.13)*	BD	**+5.49** (+3.58 to +7.39)

Table S6. The table shows the performance of **AM** (AlphaMissense), $\Delta\Delta$**G**^{AF} (FoldX5.0 predictions), and **BD** (BayesDel)-based PP3/BP4 computational evidence stratified by BRCA1 functional domain (RING vs. BRCT) and RSA (≤60% vs. >60%). We use the MAVE dataset (INT variants filtered-out) as proxy for pathogenicity and benignity. (N) Sample size (number of MAVE variants in each category) is indicated. For **AM** and $\Delta\Delta$**G**^{AF}**,** we use optimal cut-off thresholds (see Table 1 and Figure 2). For **BD**, we use cut-off thresholds defined by the ClinGen ENIGMA BRCA1 and BRCA2 variant curation expert panel. (*) The bioinformatics tool does not provide statistically significant benignity evidence of supporting strength for missense variants targeting very exposed (RSA>60%) residues (Log2 LR 95% CI overlaps -1). (**) The bioinformatics tool do not provides statistically significant pathogenicity evidence of supporting strength for missense variants targeting exposed (RSA>60%) residues (Log2 LR 95% CI overlaps +1)

Supplemental Methods

Alamut Visual Plus

We used Alamut Visual Plus version v1.6.1 (© 2022 SOPHiA GENETICS) to generate SpliceAI and VEP input vcf files.

SpliceAI-based splicing predictions

SpliceAI Δ scores [donor loss (DL), acceptor loss (AL), acceptor gain (AG), and donor gain (DG)] were calculated locally using the following parameters: genome version hg38, score type raw, and max distance ±4999. Variants were annotated as bonafide missense (i.e. spliceogenicity discarded) only if none of the four Δ scores was above the 0.20 high recall threshold.

MAVE dataset

We worked with a *BRCA1* MAVE experiment reporting RNA and functional scores for 2086 genetic variants annotated as missense (5); 651 target residues mapping to the RING domain (residues 1-101), and 1318 target residues mapping to the BRCT domain (residues 1649-1855). We calculated SpliceAI Δ scores (6) for all 2086 variants. We filtered out variants targeting the initiation codon, and variants predicted spliceogenic (MAVE RNA scores ≤-3 and/or SpliceAI Δ score ≥0.2) to generate a cohort of bonafide missense variants that included mostly singletons, but some redundant doubletons too (two different single nucleotide variants coding the same amino-acid change). For each doubleton with concordant functional scores, we filtered out the variant with the lowest RNA score. For doubletons with discordant functional scores, we filtered-out both variants. We ended up with a cohort of 1638 bonafide missense variants.

AlphaFold2 models

Models of the BRCA1 RING and BRCT domains were generated using protein prediction software AlphaFold2. We modeled the RING domain as a BRCA1/BARD1 RING-domain heterodimer, and the BRCT domain as a monomer. In brief, we generate AlphaFold2 models on LatchBio (https://console.latch.bio/) with default parameters. The pipeline generates 10 structures (five relaxed and five unrelaxed) ranked by average pLDDT. As output for Δ*ΔG* predictions, we used the best ranked (highest average pLDDT) relaxed model. Metal3D tool (https://colab.research.google.com/github/lcbc-epfl/metal-site-prediction/blob/main/Metal3D/ColabMetal.ipynb#scrollTo=IsKnyVvLXcj1) was used with default parameters to introduce Zn atoms in the correct conformational space of the BRCA1/BARD1 RING-domain heterodimer model(7). Afterwards, intramolecular distance and correct coordination bonding with Cys and His residues was evaluated using Pymol and Protein-Ligand Interaction Profiler Web tool (https://plip-tool.biotec.tu-dresden.de/plip-web/plip/index)(8).

ΔΔG predictions

To compute $\Delta\Delta G^{PDB}$ at the RING domain, we used the only available structure: a NMR solution structure (PDB 1JM7) of the human BRCA1/BARD1 RING-domain heterodimer (9). To compute $\Delta\Delta G^{PDB}$ at the BRCT domain, we selected a high-resolution (1.85 Å) X-Ray diffraction structure (PDB 1T15) of the human BRCA1 BRCT Domains in Complex with the Phosphorylated Interacting Region from Bach1 Helicase (10). To compute $\Delta\Delta G^{AF}$, we generate AlphaFold2 models for a BRCA1/BARD1 RING-domain heterodimer and for a BRCA1 BRCT monomer.

We run FoldX5.0 locally. First, we perform a preprocessing step of the input PDB file with the RepairPDB command. Later, we run the BuildModel command in triplicate to compute an average ΔΔG per missense variant.

Command line executed:

foldx –command RepairPDB –pdb protein_structure.pdb

foldX –command BuildModel –pdb protein_structure_Repair.pdb –mutant-file individual_list.txt –numberOfRuns 3

Protein_structure.pdb corresponds to experimental PDBs 1JM7and 1T15, and AF models for ΔΔG PDB and ΔΔG AF, respectively.

In the subgroup of variants targeting the RING domain, we perform the following additional analyses: (i) we label interface residues using AnalyseComplex, (ii) we label Zn-interacting residues manually (9), and (iii) we compute ΔΔG for protein-protein interaction (ΔΔGint) using the PSSM command.

Command line executed:

foldX –command AnalyseComplex –pdb protein_structure_Repair.pdb

foldX –command PSSM –pdb protein_structure_Repair.pdb

In addition, we predicted ΔΔG for the entire MAVE dataset by running SDM (11), INPS-3D (12), POPMUSIC (13), Dynamut2 (14), MAESTRO (15), and CUPSAT (16) in their corresponding web-servers (default parameters). For SDM, DYNAMUT2, INPS3D and CUPSAT we reversed the output sign, so that positive ΔΔG changes denote reduced stability. Collectively, we will refer to web-based ΔΔGs.

Case-control Validation Dataset

BRIDGES (17) variant-level counts from breast cancer cases (population-based only, N=53,572) and matched controls (N=48,048) were retrieved from bcac.ccge.medschl.cam.ac.uk/bcacdata/ (last accessed 22/10/2023). In total, 765 missense variants annotated as missense were obtained. Of these, 135 variants targeted the RING or BRCT domains. After filtering-out likely

spliceogenic (SpliceAI Δ score ≥0.2) variants, and one common variant targeting the BRCT domain [c.4956G>A p.(Met1652Ile), MAF=0.014], we end-up with a cohort of 122 bona-fide missense variants with case-control counts (**BRIDGES dataset**). Statistical analyses were performed in R. Odds ratio (OR) of cases and controls for these genes was calculated using selected ΔΔG cutoffs and methodology based on Altman, 1991(18).

Stratified LR analysis

To assign evidence weights based on the combination of RSA, AlphaMissense and ΔΔG we used the approaches summarized in Figures S9-S11.

In the first approach (**Figure S9**), we simply annotated all variants under assessment as concordant benign (if AlphaMissense ≤0.65 and $\Delta\Delta G^{AF}$ ≤Δ1.5), concordant pathogenic (if AlphaMissense ≥0.75 and $\Delta\Delta G^{AF}$ ≥Δ2.5), and others (variants with any other combination of AlphaMissense and $\Delta\Delta G^{AF}$ scores). Later, we defined a new *concordant score* coding all concordant benign variants with "-1", all concordant pathogenic variants with "+1", and all other variants with "0".Finally, we obtained Log2 LRs by running the new concordant score in an on-line LR calculator (gwiggins.shinyapps.io/lr_shiny), with cut-off thresholds set at -0.5 and +0.5.

In the second approach (**Figure S10**), we first stratified variants according to RSA into buried/partially buried variants (RSA≤60%) and exposed variants (RSA>60%), and later we analyze the *concordant score* as explained above.

In the third approach (**Figure S11**), we first stratified by RSA. Later, we used the on-line LR calculator to produce AlphaMissense-based Log2 LRs in the buried/partially-buried and exposed sub-groups. Next, we used the on-line LR calculator to produce ΔΔG-based Log2 LRs in four variant sub-groups (RSA≤60% + AlphaMissense ≤0.65, RSA≤60% + AlphaMissense ≥0.75, RS>60% + AlphaMissense ≤0.65, and RSA>60% + AlphaMissense ≥0.75). Finally, we added ΔΔG-based Log2 LR values to the corresponding AlphaMissense-based Log2 LR value to obtain combined Log2 LR values.

Supplemental Bibliography

1. Deng CX, Brodie SG. Roles of BRCA1 and its interacting proteins. Bioessays. 2000 Aug;22(8):728–37.

2. Christou CM, Kyriacou K. BRCA1 and Its Network of Interacting Partners. Biology (Basel). 2013 Jan 2;2(1):40–63.

3. Epasto LM, Pötzl C, Peterlik H, Khalil M, Saint-Pierre C, Gasparutto D, et al. NMR-identification of the interaction between BRCA1 and the intrinsically disordered monomer of the Myc-associated factor X. Protein Sci. 2024 Jan;33(1):e4849.

4. Wang Q, Zhang H, Kajino K, Greene MI. BRCA1 binds c-Myc and inhibits its transcriptional and transforming activity in cells. Oncogene. 1998 Oct 15;17(15):1939–48.

5. Findlay GM, Daza RM, Martin B, Zhang MD, Leith AP, Gasperini M, et al. Accurate classification of BRCA1 variants with saturation genome editing. Nature. 2018 Oct;562(7726):217–22.

6. Jaganathan K, Kyriazopoulou Panagiotopoulou S, McRae JF, Darbandi SF, Knowles D, Li YI, et al. Predicting Splicing from Primary Sequence with Deep Learning. Cell. 2019 Jan 24;176(3):535-548.e24.

7. Dürr SL, Levy A, Rothlisberger U. Metal3D: a general deep learning framework for accurate metal ion location prediction in proteins. Nat Commun. 2023 May 11;14(1):2713.

8. Adasme MF, Linnemann KL, Bolz SN, Kaiser F, Salentin S, Haupt VJ, et al. PLIP 2021: expanding the scope of the protein-ligand interaction profiler to DNA and RNA. Nucleic Acids Res. 2021 Jul 2;49(W1):W530–4.

9. Brzovic PS, Rajagopal P, Hoyt DW, King MC, Klevit RE. Structure of a BRCA1-BARD1 heterodimeric RING-RING complex. Nat Struct Biol. 2001 Oct;8(10):833–7.

10. Clapperton JA, Manke IA, Lowery DM, Ho T, Haire LF, Yaffe MB, et al. Structure and mechanism of BRCA1 BRCT domain recognition of phosphorylated BACH1 with implications for cancer. Nat Struct Mol Biol. 2004 Jun;11(6):512–8.

11. Pandurangan AP, Ochoa-Montaño B, Ascher DB, Blundell TL. SDM: a server for predicting effects of mutations on protein stability. Nucleic Acids Res. 2017 Jul 3;45(W1):W229–35.

12. Savojardo C, Fariselli P, Martelli PL, Casadio R. INPS-MD: a web server to predict stability of protein variants from sequence and structure. Bioinformatics. 2016 Aug 15;32(16):2542–4.

13. Dehouck Y, Kwasigroch JM, Gilis D, Rooman M. PoPMuSiC 2.1: a web server for the estimation of protein stability changes upon mutation and sequence optimality. BMC Bioinformatics. 2011 May 13;12:151.

14. Rodrigues CHM, Pires DEV, Ascher DB. DynaMut2: Assessing changes in stability and flexibility upon single and multiple point missense mutations. Protein Sci. 2021 Jan;30(1):60–9.

15. Laimer J, Hiebl-Flach J, Lengauer D, Lackner P. MAESTROweb: a web server for structure-based protein stability prediction. Bioinformatics. 2016 May 1;32(9):1414–6.

16. Parthiban V, Gromiha MM, Schomburg D. CUPSAT: prediction of protein stability upon point mutations. Nucleic Acids Res. 2006 Jul 1;34(Web Server issue):W239-242.

17. Breast Cancer Association Consortium, Dorling L, Carvalho S, Allen J, González-Neira A, Luccarini C, et al. Breast Cancer Risk Genes - Association Analysis in More than 113,000 Women. N Engl J Med. 2021 Jan 20;

18. Altman DG. Practical Statistics for Medical Research (1st ed.). [Internet]. New York: Chapman and Hall/CRC; 1990. 624 p. Available from: https://doi.org/10.1201/9780429258589
