# Supplementary material for: ACMG/AMP interpretation of BRCA1 missense variants: Structure-informed scores add evidence strength granularity to the PP3/BP4 computational evidence
Source: Am J Hum Genet. 2025 Apr 14;112(5):993–1002. doi: 10.1016/j.ajhg.2024.12.011 (PMC12120176; doi:10.1016/j.ajhg.2024.12.011)
Supplement: Document S2. Article plus supplemental information [file mmc3.pdf]

# ACMG/AMP interpretation of *BRCA1* missense variants: Structure-informed scores add evidence strength granularity to the PP3/BP4 computational evidence

## Graphical abstract

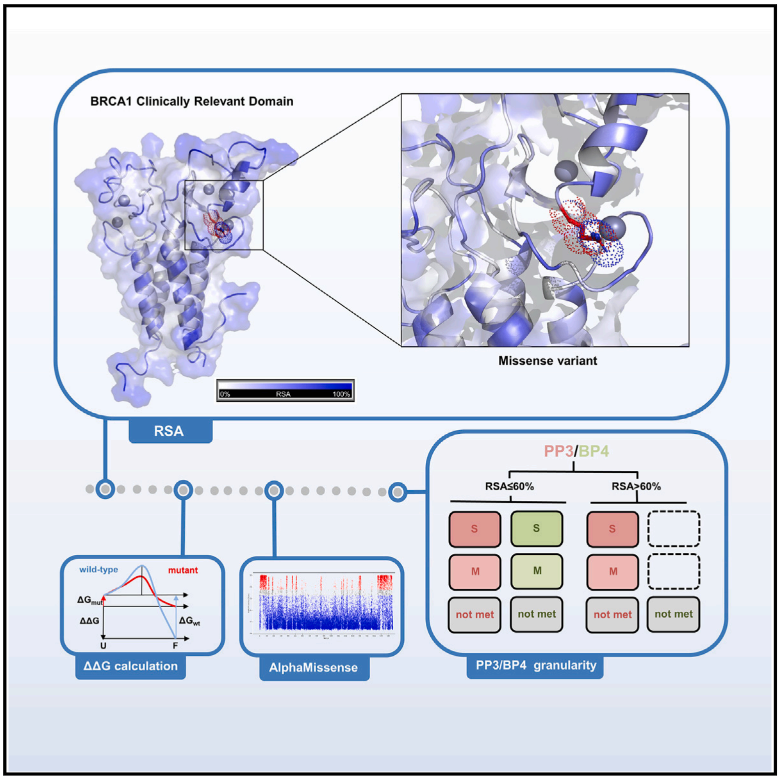


## Authors

Lobna Ramadane-Morchadi,
Nitsan Rotenberg,
Ada Esteban-Sánchez, ...,
Pedro Pérez-Segura,
Amanda B. Spurdle, Miguel de la Hoya

## Correspondence

miguel.hoya@salud.madrid.org

**Specifications for the BRCA1 ACMG/AMP classification system recommend supporting strength for application of PP3/BP4 computational evidence. Focusing on missense variants targeting BRCA1 clinically relevant domains, we show that a structure-informed approach combining RSA with AlphaMissense and ΔΔG provides evidence strength granularity, with PP3/BP4 moderate or strong strength met by certain variants.**

Ramadane-Morchadi et al., 2025, The American Journal of Human Genetics
*112*, 993–1002
May 1, 2025 © 2024 The Author(s). Published by Elsevier Inc. on behalf of American Society of Human Genetics.
https://doi.org/10.1016/j.ajhg.2024.12.011

# ARTICLE

# ACMG/AMP interpretation of *BRCA1* missense variants: Structure-informed scores add evidence strength granularity to the PP3/BP4 computational evidence

Lobna Ramadane-Morchadi,[1] Nitsan Rotenberg,[2,3] Ada Esteban-Sánchez,[1] Cristina Fortuno,[3] Alicia Gómez-Sanz,[1] Matthew J. Varga,[4] Adam Chamberlin,[4] Marcy E. Richardson,[4] Kyriaki Michailidou,[5] Pedro Pérez-Segura,[1] Amanda B. Spurdle,[2,3] and Miguel de la Hoya[1,*]

## Summary

Classification of missense variants is challenging. Lacking compelling clinical and/or functional data, ACMG/AMP lines of evidence are restricted to PM2 (rarity code applied at supporting level) and PP3/BP4 (computational evidence based mostly on multiple-sequence-alignment conservation tools). Currently, the ClinGen ENIGMA *BRCA1/2* Variant Curation Expert Panel uses BayesDel to apply PP3/BP4 to missense variants located in the BRCA1 RING/BRCT domains. The ACMG/AMP framework does not refer explicitly to protein structure as a putative source of pathogenic/benign evidence. Here, we tested the value of incorporating structure-based evidence such as relative solvent accessibility (RSA), folding stability (ΔΔG), and/or AlphaMissense pathogenicity to the classification of *BRCA1* missense variants. We used MAVE functional scores as proxies for pathogenicity/benignity. We computed RSA and FoldX5.0 ΔΔG predictions using as alternative input templates for either PDB files or AlphaFold2 models, and we retrieved pre-computed AlphaMissense and BayesDel scores. We calculated likelihood ratios toward pathogenicity/benignity provided by the tools (individually or combined). We performed a clinical validation of major findings using the large-scale BRIDGES case-control dataset. AlphaMissense outperforms ΔΔG and BayesDel, providing similar PP3/BP4 evidence strengths with lower rate of variants in the uninformative score range. AlphaMissense combined with ΔΔG increases evidence strength granularity. AlphaFold2 models perform well as input templates for ΔΔG predictions. Regardless of the tool, BP4 (but not PP3) is highly dependent on RSA, with benignity evidence provided only to variants targeting buried or partially buried residues (RSA $\leq$ 60%). Stratification by functional domain did not reveal major differences. In brief, structure-based analysis improves PP3/BP4 assessment, uncovering a relevant role for RSA.

## Introduction

The introduction of next-generation sequencing in the clinical setting has revolutionized genetic diagnostics. However, the identification of an ever-growing number of genetic variants of uncertain significance (VUSs) presents a major challenge in the clinical interpretation of the findings. For genes where loss-of-function is the gene/disease association mechanism, most nonsense/frameshift variants are readily annotated as pathogenic, but assessment of missense changes is far more complex.

Mounting evidence suggests that a high proportion of missense changes have little or no effect on protein function, albeit some are severely damaging[1] and, depending on the specific gene, the ratio of tolerated/damaging changes is very variable.[2] Several studies have shown that reduced thermodynamic stability is a major driver of pathogenicity for missense variants.[1,3–6]

Studies conducted in cancer susceptibility genes have shown that missense variants predicted to be destabilizing are likely non-functional.[5,7,8] However, the reverse is not necessarily true, as missense variants not disturbing stability may still cause loss of function via other mechanisms, such as perturbing critical protein-protein or protein-ligand interactions.[9]

Many computational algorithms predict Gibbs free energy changes (ΔΔG) in protein folding (or protein interaction) upon mutation, with FoldX[10] outperforming others in identifying pathogenic missense variants.[11] Since FoldX uses Protein Data Bank atomic coordinate (PDB) files as input templates, the availability of experimental structures might limit the ability of structure to provide evidence. Recently, deep-learning algorithms have dramatically improved the accuracy of structure predictions from amino acid sequences, potentially expanding the clinical application of ΔΔG predictions to any protein of interest. AlphaFold2 has demonstrated outstanding performance, predicting the structure of protein globular domains with an accuracy matching X-ray crystallography, nuclear magnetic resonance (NMR), or cryogenic electron microscopy data.[12,13] Assessment of AlphaFold2 models as templates to evaluate protein structural features show results that (for high-confidence predicted regions) consistently match or surpass those obtained with experimental templates.[14]

Recently, Google DeepMind has developed AlphaMissense, a machine-learning tool that utilizes the AlphaFold2-based structural context to predict pathogenicity

[1]Molecular Oncology Laboratory, Hospital Clínico San Carlos, IdISSC (Instituto de Investigación Sanitaria del Hospital Clínico San Carlos), 28040 Madrid, Spain; [2]University of Queensland, Brisbane, QLD, Australia; [3]Molecular Cancer Epidemiology Laboratory, QIMR Berghofer MRI, Herston, QLD 4006, Australia; [4]Ambry Genetics, Aliso Viejo, CA 92656, USA; [5]Biostatistics Unit, The Cyprus Institute of Neurology & Genetics, 2371 Nicosia, Cyprus
*Correspondence: miguel.hoya@salud.madrid.org
https://doi.org/10.1016/j.ajhg.2024.12.011.
© 2024 The Author(s). Published by Elsevier Inc. on behalf of American Society of Human Genetics.
This is an open access article under the CC BY license (http://creativecommons.org/licenses/by/4.0/).

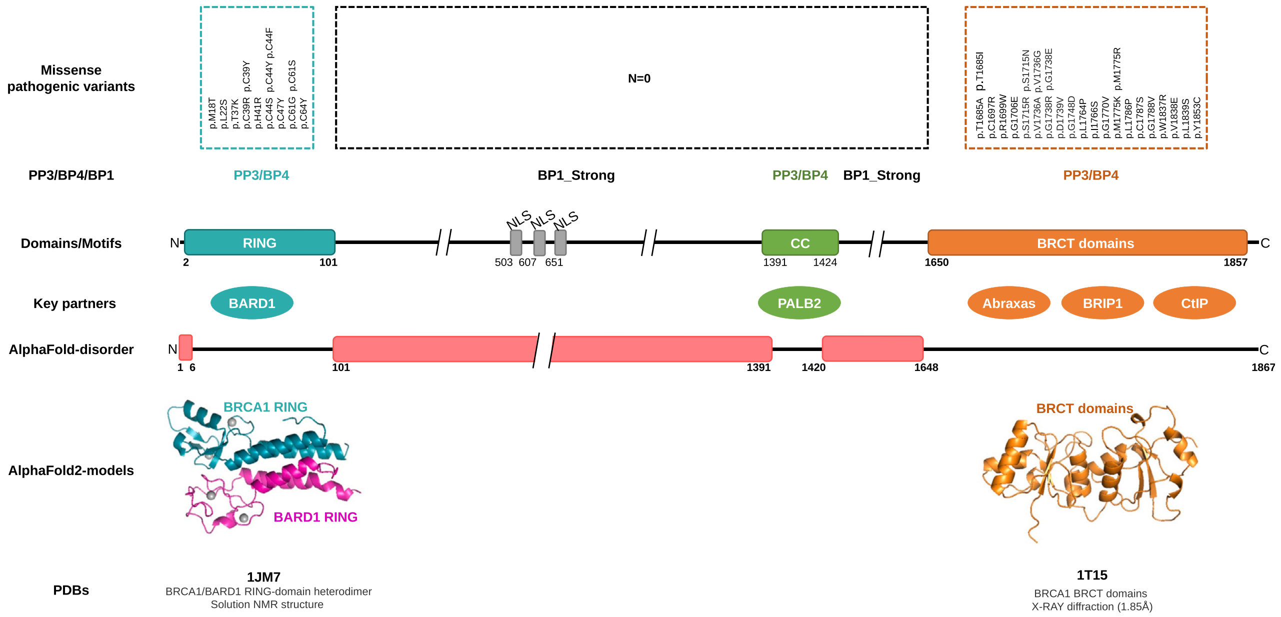


**Figure 1. Summary of relevant structural, functional, and clinical annotations of the 1,867-aa BRCA1 protein**

The Missense pathogenic variants track displays all BRCA1 missense changes ($N = 38$) classified as pathogenic by the ClinGen ENIGMA BRCA1/2 VCEP (last consulted August 22, 2024) (scale not preserved). Note that pathogenic missense variants cluster at the RING and BRCT domains, with no pathogenic missense variants reported so far in other regions. The PP3/BP4/BP1 track summarizes ClinGen ENIGMA BRCA1/2 VCEP rules to apply ACMG/AMP predictive evidence to BRCA1 missense changes. Depending on BayesDel-noAF scores, PP3 ($\geq 0.28$) or BP4 ($\leq 0.15$) computational evidence is applied to missense variants targeting the RING, CC, or BRCT domains. By contrast, PP3/BP4 is not applied to missense variants targeting other regions (mostly, disordered regions). Instead, the BP1_Strong code is applied (regardless of computational predictions). The Domains/Motifs cartoon track represents BRCA1 conserved domains/motifs as defined by the ClinGen ENIGMA BRCA1/2 VCEP, with the RING and BRCT domains defined as clinically important functional domains and the CC (coiled-coil) motif as potentially clinically important (note that the precise boundaries of these domains might be slightly different according to other sources such as UniProt: P38398). The Key partners track shows BRCA1 key interacting proteins BARD1 (interacting with the RING domain), PALB2 (interacting with the CC motif), Abraxas, BRIP1 (also known as BACH1), and CtIP (the latter three interacting with the BRCT domains). The AlphaFold-disorder cartoon track represents BRCA1 disordered regions as deduced from the AlphaFold-2 model AF-P38398-F1 (p.LDDT score $<70$). The AlphaFold2-models track displays the BRCA1/BARD1 RING heterodimer and BRCT-domain AlphaFold2 models generated for this study. The PDBs track shows ID, descriptive name, and method for experimentally determined 3D structures used in this study.

for all human proteome missense variants.[15] A study comparing predictions with data from multiplexed assays of variant effect (MAVE) suggests that AlphaMissense outperforms existing multiple-sequence-alignment conservation (meta-)predictors.[16]

ClinGen Variant Curation Expert Panel (VCEP) classification using gene-level specifications of the American College of Medical Genetics and Genomics/Association for Molecular Pathology (ACMG/AMP) framework,[17] a process that has Food and Drug Administration (FDA) recognition, is increasingly acknowledged as a gold standard for clinical classification of germline variants in Mendelian disease genes. Despite the comprehensiveness of the framework, classification of rare missense variants remains challenging, and many variants remain VUSs after VCEP review. In the absence of compelling clinical and/or functional data, ACMG/AMP pathogenicity/benignity lines of evidence are restricted to the absence/rarity code PM2 with strength level decreased by ClinGen to "supporting" (SVI recommendation for absence/rarity PM2 v.1.0, available at https://clinicalgenome.org/working-groups/sequence-variant-interpretation), and computational codes PP3/BP4, based mostly on multiple-sequence-alignment conservation (meta-)predictors.[18,19] Surprisingly, the current ACMG/AMP framework does not refer explicitly to structural features as a putative source of pathogenic/benign evidence and certainly does not incorporate structure-based pathogenicity and/or benignity codes.

Currently, the ClinGen ENIGMA *BRCA1* and *BRCA2* VCEP[20] uses scores generated by the meta-predictor BayesDel[21] to apply PP3/BP4 computational evidence to *BRCA1* missense variants located in the really interesting new gene (RING), coiled-coil (CC), and BRCA1 C-terminal (BRCT) domains (see Figure 1 for further details). Here, we tested the value of incorporating structural features, based on ΔΔG predictions and/or AlphaMissense pathogenicity scoring, for the ACMG/AMP classification of *BRCA1* missense variants. Overall, we show that regardless of the *BRCA1* functional domain analyzed (RING or BRCT), AlphaMissense outperforms BayesDel (PP3/BP4 moderate evidence strength applied to more variants), ΔΔG stratifies AlphaMissense evidence strength, and relative solvent accessibility (RSA) is a critical factor in evaluating the PP3/BP4 computational evidence (PP3/BP4 performing better for buried/partially buried residues than for exposed residues). In addition, our analysis suggests that regions other

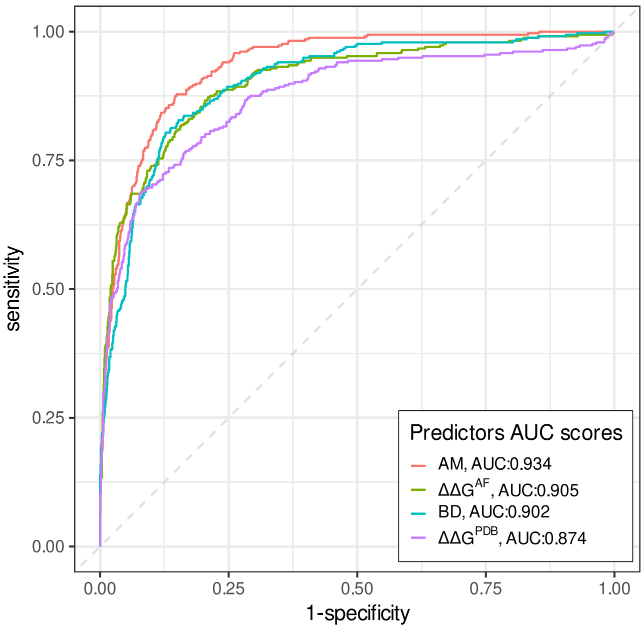


**Figure 2. AlphaMissense, $\Delta\Delta G^{PDB}$, $\Delta\Delta G^{AF}$, and BayesDel performance at discriminating LoF and FUNC variants at the RING and BRCT domains**
The figure displays ROC plots and the corresponding auROC value. Overall, AlphaMissense (AM) provides the best discrimination. $\Delta\Delta G^{AF}$ outperforms $\Delta\Delta G^{PDB}$.

than the RING, CC, and BRCT domains might be functionally important.

## Material and methods

Starting with a *BRCA1* MAVE experiment reporting RNA and functional scores for 2,086 genetic variants annotated as missense,[22] we generated a test cohort of 1,638 bona fide missense variants (536 targeting RING and 1,102 targeting BRCT residues) with associated functional data (MAVE dataset, see supplemental methods). The MAVE dataset included 1,182 variants scoring functional (FUNC, score $> -0.748$), 337 variants scoring non-functional (LoF, score $< -1.328$), and 119 variants scoring intermediate (INT, $-1.328 <$ score $< -0.748$) (Table S1).

We predicted ΔΔG with FoldX5.0[10] and six web-based methods. As input templates for FoldX5.0, we used experimental PDB files ($\Delta\Delta G^{PDB}$) and AlphaFold2 models ($\Delta\Delta G^{AF}$) (see supplemental methods for further details). All predicted ΔΔG values ($N$ = 14,742) are shown in Table S1.

RSA was computed with the psa module of JOY.[23] Computed RSA scores ($N$ = 1,638) are shown in Table S1. Variants were stratified into those targeting buried (RSA < 30%), partially buried (30% ≤ RSA ≤ 60%), and exposed (RSA > 60%) residues. BayesDel scores (see Table S1) were retrieved from the database for non-synonymous SNPs' functional predictions (dbNSFP) using the Ensembl Variant Effect Predictor (https://www.ensembl.org/Tools/VEP).[24] Pre-computed AlphaMissense scores (see Table S1) were retrieved at console.cloud.google.com/storage/browser/dm_alphamissense.

Statistical analyses and graphical plots were performed using the statistical software R and packages ggplot2 (https://ggplot2.tidyverse.org), ROCR,[25] and pROC.[26] Statistical comparison of LoF, FUNC, and INT distributions was performed with non-parametric Wilcoxon test with the R ggpubr package (https://rpkgs.datanovia.com/ggpubr/). Differences between areas under the receiver-operating characteristics curve (auROCs) were assessed using the roc.test function of pROC through 10,000 bootstraps.[26] The likelihood ratios toward pathogenicity or benignity provided by different AlphaMissense, ΔΔG, and BayesDel cutoff scores were assessed using an R-based online tool set up to simplify likelihood ratio (LR) calculations for bioinformatic prediction tool categories (gwiggins.shinyapps.io/lr_shiny). To transform LRs into evidence strengths, we followed recommendations arising from the Bayesian modeling of the ACMG/AMP rules.[27] Accordingly, $\log_2$ LRs rounded to 1, 2, and 4 (point scores) equate to ACMG/AMP Supporting, Moderate, and Strong evidence strength, respectively.[28] Stratified LR analysis was used to assign evidence weights to variants based on the combination of RSA, AlphaMissense, and ΔΔG (see supplemental methods for further details). Diagnostic test evaluation was performed online with MedCalc statistical software (www.medcalc.org/calc).

We retrieved variant-level counts for 122 bona fide missense variants targeting the BRCA1 RING or BRCT domains from the Breast Cancer After Diagnostic Gene Sequencing (BRIDGES) breast cancer association study[29] (see supplemental methods).

## Results

We have evaluated missense variants targeting the *BRCA1* clinically relevant domains RING or BRCT and for which MAVE functional data were available (Table S1). In total, we have evaluated 1,638 missense variants (536 targeting the RING domain and 1,102 targeting the BRCT domain) with MAVE data indicating LoF ($n$ = 337), INT ($n$ = 119), or FUNC ($n$ = 1,182). Stratification by functional domain did not reveal differences, with variants demonstrating impaired activity (LoF + INT) representing 28% of the variants in both domains. In contrast, stratification by RSA revealed major differences, with impaired activity variants representing 42% of the variants targeting buried residues ($n$ = 879), 16% of the variants targeting partially buried residues ($n$ = 331), and 7% of the variants targeting exposed residues ($n$ = 428). We observed this trend in both domains, but it appeared most striking in the subgroup of variants targeting the BRCT domain. Table S2 and Figures S1A and S1B summarize relevant features of the MAVE dataset stratified by domain, RSA, functional category, or residue subtype (within the RING domain).

Using PDB files as input templates ($\Delta\Delta G^{PDB}$), FoldX5.0 predicted, on average, a strong destabilizing effect for LoF variants (ΔΔG = +5.77 kcal/mol), a mild destabilizing effect for FUNC variants (ΔΔG = +0.64 kcal/mol), and an intermediate destabilizing effect for INT variants (ΔΔG = +3.09 kcal/mol). Using AlphaFold2 models ($\Delta\Delta G^{AF}$), we observed similar results, with average destabilizing impacts of +6.55 kcal/mol for LoF, +1.04 kcal/mol for FUNC, and +3.41 kcal/mol for INT variants. $\Delta\Delta G^{PDB}$ (or $\Delta\Delta G^{AF}$) stratification per domain or per RSA suggested that the average destabilizing effect is higher both for variants targeting RING residues and for variants targeting buried residues (Table S2; Figures S2 and S3).

**Table 1. PP3/BP4 computational evidence based on AlphaMissense, $\Delta\Delta G^{AF}$, $\Delta\Delta G^{PDB}$, or BayesDel at different benignity and pathogenicity cutoff thresholds**

| | Benignity evidence (BP4) | | No bioinformatic code applicable | Pathogenicity evidence (PP3) | |
|---|---|---|---|---|---|
| | Threshold | Evidence strength, $\log_2$ LR (95% CI) | | Threshold | Evidence strength, $\log_2$ LR (95% CI) |
| AM | ≤0.34[a] | −4.603 (−5.535 to −3.671) | 12% | ≥0.56[a] | +2.083 (+1.919 to +2.247) |
| | ≤0.60 | −3.038 (−3.508 to −2.569) | 10% | ≥0.80 | +3.007 (+2.746 to +3.269) |
| | ≤0.65 | −2.914 (−3.354 to −2.474) | 5% | ≥0.75 | +2.810 (+2.578 to +3.042) |
| $\Delta\Delta G^{AF}$[b] | ≤+1.0 | −3.417 (−4.051 to −2.784) | 26% | ≥+3.0 | +2.691 (+2.448 to +2.933) |
| | ≤+1.5 | −2.946 (−3.433 to −2.456) | 8% | ≥+2.5 | +2.367 (+2.164 to +2.570) |
| $\Delta\Delta G^{PDB}$[b] | ≤+1.0 | −2.663 (−3.135 to −2.191) | 26% | ≥+3.0 | +2.780 (+2.515 to +3.045) |
| | ≤+1.5 | −2.264 (−2.632 to −1.895) | 12% | ≥+2.5 | +2.309 (+2.093 to +2.525) |
| BD | ≤0.15[c] | −2.923 (−3.401 to −2.444) | 14% | ≥0.28[c] | +2.643 (+2.415 to +2.872) |

[a]Generic thresholds as per AlphaMissense developers (note that benignity evidence strength is very strong but pathogenicity evidence strength is weaker, and rate of variants in the uninformative score range is high).
[b]FoldX5.0 predictions.
[c]BP4/PP3 BRCA1 VCEP thresholds.

Overall, the data suggest that MAVE functional class stratification by $\Delta\Delta G$ is better for variants targeting buried/partially buried residues (RSA ≤ 60%) than for variants targeting exposed residues (RSA > 60%). In the latter case, we did not observe statistically significant differences in the average $\Delta\Delta G^{PDB}$ (or $\Delta\Delta G^{AF}$) value of INT and FUNC variants (Figures S2 and S3). Stratification by functional domain (RING vs. BRCT) did not reveal major differences (Figures S2 and S3).

Interestingly, AlphaMissense and BayesDel showed similar trends, with INT variants displaying intermediate scores, variants targeting RING residues scoring higher than variants targeting BRCT residues, and variants targeting buried residues scoring higher than others (Table S2). As observed for $\Delta\Delta G$, MAVE functional class stratification by AlphaMissense was better for variants targeting buried/partially buried residues (RSA ≤ 60%) than for variants targeting exposed residues (RSA > 60%), with no major differences between RING and BRCT domains (Figure S4). BayesDel performed similarly, with some evidence that it might outperform $\Delta\Delta G$ and AlphaMissense at variants targeting exposed (RSA>60%) residues (Figure S5).

Overall, the (negative) correlation of AlphaMissense with MAVE functional scores ($r = -0.67$) is higher compared to the correlation with BayesDel ($r = -0.61$), $\Delta\Delta G^{AF}$($r = -0.55$), and $\Delta\Delta G^{PDB}$ ($r = -0.51$). Note that $\Delta\Delta G^{AF}$ correlated better than $\Delta\Delta G^{PDB}$ (Figure S6).

We next tested the performance of $\Delta\Delta G^{AF}$, $\Delta\Delta G^{PDB}$, AlphaMissense, and BayesDel to distinguish LoF (used here as a proxy for pathogenicity) from FUNC variants (proxy for benignity). For this analysis, we filtered out INT variants (the association of partial activity with disease predisposition is unclear), restricting our analysis to an MAVE cohort of 1,519 variants (337 LoF and 1,182 FUNC). Overall, AlphaMissense (auROC = 0.93) outperformed $\Delta\Delta G^{AF}$ (auROC = 0.91, $p = 0.003$), BayesDel (auROC = 0.90, $p = 0.0001$), and $\Delta\Delta G^{PDB}$ (auROC = 0.87, $p = 1.2 \times 10^{-6}$) (Figure 2).

Six web-based $\Delta\Delta G$ predictors perform poorly, with auROCs ranging from 0.64 (CUPSAT) to 0.78 (INPS3D), and correlation with MAVE functional scores ranging from $r = -0.21$ (CUPSAT) to $r = -0.44$ (INSP3D) (Figures S6 and S7).

Stratification by functional domain showed that: (1) AlphaMissense and $\Delta\Delta G^{AF}$ (each with auROC = 0.94) outperformed BayesDel (auROC = 0.92, $p$ not significant) and $\Delta\Delta G^{PDB}$ (auROC = 0.86, $p = 0.003$) at discriminating LoF and FUNC variants at the RING domain ($N = 408$); and (2) AlphaMissense (auROC = 0.95) outperformed BayesDel (auROC = 0.90, $p = 1.2 \times 10^{-5}$), $\Delta\Delta G^{AF}$ (auROC = 0.89, $p = 3.9 \times 10^{-6}$), and $\Delta\Delta G^{PDB}$ (auROC = 0.88, $p = 3.7 \times 10^{-8}$) at discriminating LoF and FUNC variants in the BRCT ($N = 1{,}032$) domain. Note that overall, $\Delta\Delta G^{AF}$ outperformed $\Delta\Delta G^{PDB}$, mostly due to a better performance in the RING domain (Figure S8).

We next evaluated the performance of computational evidence based on AlphaMissense or $\Delta\Delta G$ (FoldX5.0 predictions) and how it compared with the BayesDel-based PP3/BP4 evidence currently specified for the ACMG/AMP classification of *BRCA1* missense variants. To start with, we analyzed the evidence strength against (or toward) pathogenicity provided by AlphaMissense at various thresholds, including an uninformative score-range category (i.e., bioinformatic evidence not applicable) centered at the optimal binary cutpoint. A trade-off iterative process aimed at maximizing strength of evidence and minimizing the proportion of variants in the uninformative range led us to conclude that AlphaMissense performed well with benignity/pathogenicity thresholds set near 0.7. Applying ≤0.65 (benignity) and ≥0.75 (pathogenicity) thresholds, 1,015 variants would receive evidence in the benign direction ($\log_2$ LR = −2.91) and 426 variants in the pathogenic direction ($\log_2$ LR = +2.81), while only 78 variants (5%) would fall in the uninformative score range (Table 1 and Figure 3).

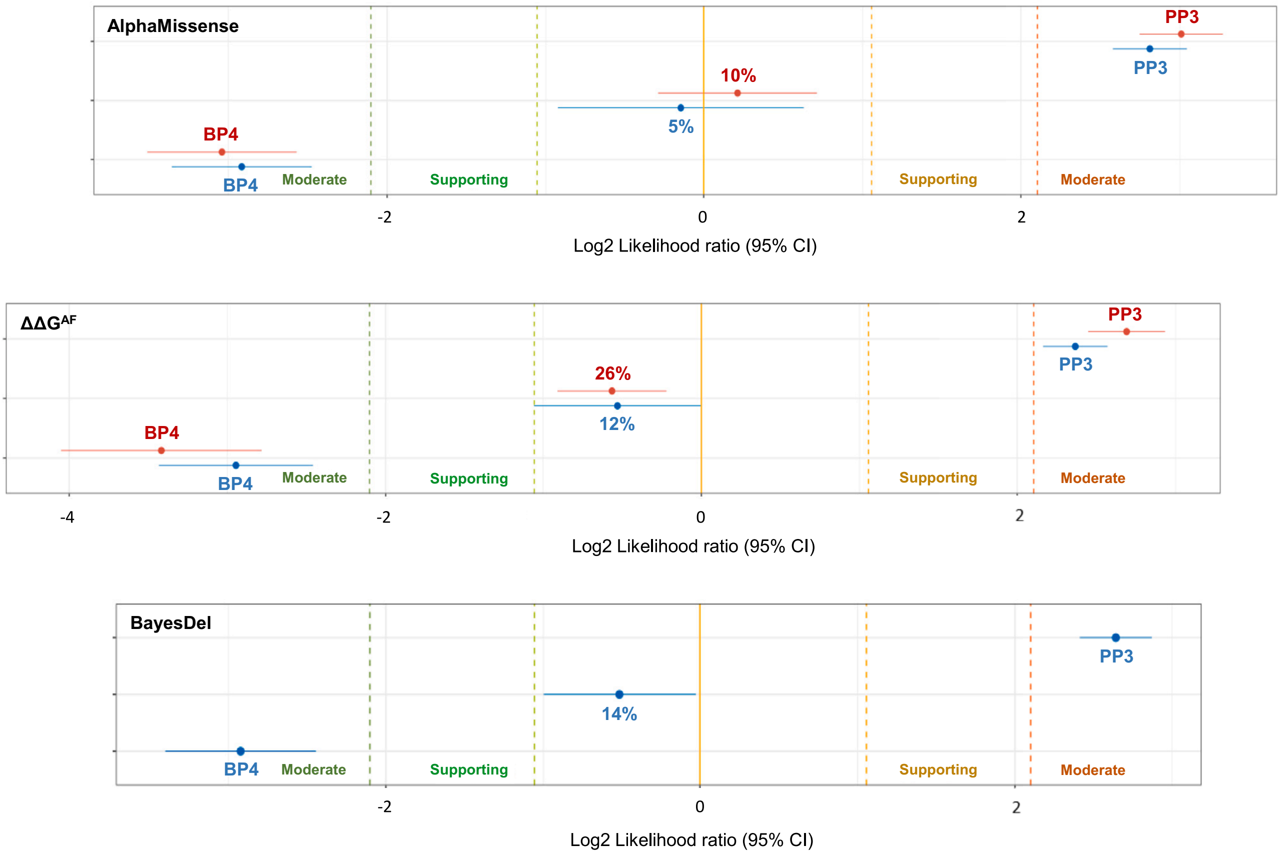


**Figure 3. PP3/BP4 computational evidence strengths provided by AlphaMissense, $\Delta\Delta G^{AF}$, and BayesDel**
AlphaMissense evidence strengths using ≤0.6 (BP4)//≥0.8 (PP3) (red) or ≤0.65 (BP4)//≥0.75 (PP3) (blue) thresholds (top). $\Delta\Delta G^{AF}$ evidence strengths using ≤+1 kcal/mol (BP4)//≥+3 kcal/mol (PP3) (red) or ≤+1.5 kcal/mol (BP4)//≥+2.5 kcal/mol (PP3) (blue) thresholds (middle). BayesDel evidence strength with ClinVar ENIGMA BRCA1/2 VCEP recommended thresholds (bottom). Percent of variants falling in the non-informative score range. $\log_2$ LR calculations and plot (including Log2 LR 95% confidential intervals) generated at gwiggins.shinyapps.io/lr_shiny/.

A similar trade-off iterative process led us to conclude that $\Delta\Delta G^{AF}$ performed well with benignity/pathogenicity thresholds set near +2.0 kcal/mol. Applying ≤+1.5 and ≥+2.5 kcal/mol thresholds, 869 variants received evidence of benignity and 462 variants received evidence of pathogenicity ($\log_2$ LR = −2.95 and +2.37, respectively), with 12% of the variants falling in the uninformative score range. See Table 1 and Figure 2 for further details. Of note, $\Delta\Delta G^{PDB}$ did not outperform $\Delta\Delta G^{AF}$ either in benignity/pathogenicity evidence strengths ($\log_2$ LRs) or in the rate of variants in the uninformative range (Table 1).

Using current *BRCA1* VCEP BayesDel thresholds for BP4 and PP3, 883 missense variants receive evidence of benignity (and 420 evidence of pathogenicity) ($\log_2$ LR = −2.92 and +2.64), and 216 variants (14%) fall in the uninformative score range (0.15 < BayesDel < 0.28). See Table 1 and Figure 2 for further details.

In brief, AlphaMissense, ΔΔG (FoldX5.0 predictions), and BayesDel provide evidence toward pathogenicity ($+2 < \log_2 \text{LR} < +4$) and benignity ($-4 < \log_2 \text{LR} < -2$) with evidence strengths in the moderate to strong range. A diagnostic test evaluation does not reveal major differences between the tools (Table 2).

We conclude that an AlphaMissense-based PP3/BP4 evidence would outperform BayesDel-based (or ΔΔG-based) PP3/BP4 evidence, since it would provide similar pathogenicity and benignity evidence strengths but with a lower rate of variants in the uninformative score range (Table 1). Stratification by functional domain (RING vs. BRCT) did not reveal major differences, with all three computational tools supporting moderate to strong pathogenicity/benignity evidence strength (and AlphaMissense providing a lower rate of variants in the uninformative score range) in both domains (Table S3).

Burden-type association analysis (see material and methods) confirmed that, on average, AlphaMissense ≥ 0.75 (odds ratio [OR] = 4.69), $\Delta\Delta G^{AF}$ ≥ 2.5 kcal/mol (OR = 4.35), $\Delta\Delta G^{PDB}$ ≥ 2.5 kcal/mol (OR = 3.62), and BayesDel ≥ 0.28 (OR = 3.59) each identify *BRCA1* missense variants with clinically actionable BC risk levels.[30] Equally relevant, the analysis confirmed that variants scoring below PP3 thresholds are not associated with clinically actionable risk levels (ORs < 1.3) (Table 3 and Figure 4).

Stratification by domain (RING vs. BRCT) did not reveal major differences (Table S4). Further, the analysis revealed

**Table 2. Diagnostic test evaluation**

| Threshold | Sensitivity | Specificity | PPV | NPV | Accuracy |
|---|---|---|---|---|---|
| **AM** | | | | | |
| ≥0.75 | 84.3 (79.9–88.0) | 88.0 (86.0–89.8) | 66.7 (63.0–70.1) | 95.2 (93.8–96.2) | 87.2 (85.4–88.8) |
| **ΔΔG$^{AF}$[a]** | | | | | |
| ≥+3 | 89.1 (84.9–92.5) | 85.9 (83.5–88.0) | 64.0 (60.2–67.6) | 96.6 (92.3–97.5) | 86.6 (84.6–88.4) |
| ≥+2.5 | 81.6 (77.1–85.6) | 84.2 (81.2–86.2) | 59.5 (56.1–62.8) | 94.1 (92.8–95.3) | 83.7 (81.7–85.4) |
| **ΔΔG$^{PDB}$[a]** | | | | | |
| ≥+3 | 87.7 (83.1–91.4) | 85.9 (83.4–88.2) | 63.7 (59.7–67.6) | 96.1 (94.7–97.2) | 86.3 (84.2–88.3) |
| ≥+2.5 | 74.2 (69.2–78.8) | 85.0 (82.9–87.0) | 58.5 (54.9–62.1) | 92.0 (90.6–93.3) | 82.6 (80.6–84.5) |
| **BD** | | | | | |
| ≥0.28 | 79.8 (75.1–84.0) | 87.2 (85.2–89.1) | 64.1 (60.3–67.6) | 93.8 (92.5–94.9) | 85.6 (83.7–87.3) |

With the indicated thresholds, the table shows the performance of AlphaMissense, ΔΔG$^{AF}$, ΔΔG$^{PDB}$, and BayesDel discriminating MAVE LoF variants. AM, AlphaMissense; BD, BayesDel; PPV, positive predictive value; NPV, negative predictive value.
[a]FoldX5.0 predictions.

that ΔΔG (or BayesDel) scoring provides clinically relevant risk stratification to AlphaMissense ≥ 0.75 variants. On average, concordant scores identified high-risk variants (ORs > 6.0), while discordant scores identified variants not associated with risk (Figure 4).

Since average ΔΔG, AlphaMissense, and BayesDel scoring of LoF, INT, and FUNC variants was influenced by RSA, we suspected that pathogenicity/benignity evidence strength might be RSA dependent too. To test the hypothesis, we first analyzed ΔΔG$^{AF}$ discriminating performance stratifying target residues into buried (RSA ≤ 30%), partially buried (30% < RSA ≤ 60%), and exposed (RSA > 60%). Overall, we observe that ΔΔG$^{AF}$ (≥+2.5 kcal/mol) provides evidence toward pathogenicity regardless of RSA. By contrast, ΔΔG$^{AF}$ (≤+1.5 kcal/mol) provides evidence toward benignity for buried/partially buried but not for exposed residues (Table S5). ΔΔG$^{PDB}$-based analysis provided similar results (Table S5). Interestingly, the target residue RSA influenced AlphaMissense-based and BayesDel-based computational evidence similarly. For variants targeting exposed residues, neither AlphaMissense nor BayesDel provided statistically significant evidence toward benignity (Table S5). This RSA influence on computational evidence was observed in both the RING and BRCT domains (Table S6).

We next explored the possibility of combining AlphaMissense with other computational scores to maximize PP3/BP4 code utility in the ACMG/AMP classification of *BRCA1* missense variants. Since RSA, ΔΔG$^{PDB}$, and ΔΔG$^{AF}$ correlations with AlphaMissense are moderate ($r = -0.49$, $+0.46$, and $+0.50$, respectively), while BayesDel correlation ($r = +0.74$) is high (Figure S6), we focused our analysis on combining AlphaMissense with RSA and ΔΔG. We tested different approaches. Computational evidence based on AlphaMissense/ΔΔG concordance provided Strong level of pathogenic and benign evidence at the cost of a high rate of variants (31%) in the uninformative score range (Figure S9). RSA stratification revealed that AlphaMissense/ΔΔG concordance does not provide statistically significant benignity evidence at even Supporting strength to variants targeting exposed residues (Figure S10), confirming the relevance of RSA stratification in computational scoring. In our hands, cascade stratification by RSA, AlphaMissense, and ΔΔG performed well for variants targeting buried/partially buried residues, providing pathogenic/benign evidence strength stratification into Strong and Moderate, with only 16% of variants falling in the uninformative score range (Figure S11). Regarding variants targeting exposed residues, cascade stratification improved the rate of variants with evidence of pathogenicity (Moderate or Strong strength) but failed to provide evidence of benignity with Supporting strength (Figure S11).

Finally, we performed a protein-wide AlphaMissense analysis to investigate whether pathogenic *BRCA1* missense variants might cluster in regions other than the RING and BRCT domains. The analysis suggested that, in addition to CC residues 1,391–1,424 (already highlighted as "potentially clinically important" in the BRCA1/2 VCEP specifications, see Figure 1), BRCA1 regions spanning residues 127–133, 180–185, 378–386, 458–465, 515–519, and 853–869 might be candidates for further investigation and potentially important for impact on protein function (Figure S12).

## Discussion

In the present study, we have evaluated the AlphaMissense-based and ΔΔG-based PP3/BP4 computational evidence contribution to the ACMG/AMP classification of *BRCA1* missense variants located at the RING and BRCT domains and how these compare with the current BayesDel-based PP3/BP4 applied by the *BRCA1* VCEP. The analysis is based on a *BRCA1* MAVE study,[22] using LoF and FUNC categories as proxies for pathogenicity and benignity. We showed

Table 3. **BRIDGES-based breast cancer risk estimates (burden analysis) stratified by AlphaMissense, $\Delta\Delta G^{AF}$, $\Delta\Delta G^{PDB}$, and BayesDel scoring**

| Threshold | Unique *BRCA1* missense variants | BC[a] | Controls[b] | OR (95% CI) | *p* |
|---|---|---|---|---|---|
| **AM** | | | | | |
| $\geq$0.75 | 33 | 94 | 18 | *4.69 (2.83–7.76) | *$4.15 \times 10^{-9}$ |
| 0.65 < AM < 0.75 | 7 | 22 | 16 | 1.23 (0.68–2.35) | 0.53 |
| $\leq$0.65 | 82 | 146 | 101 | 1.30 (1.01–1.66) | 0.04 |
| **$\Delta\Delta G^{AF}$[c]** | | | | | |
| $\geq$+2.5 | 32 | 97 | 20 | *4.35 (2.69–7.05) | *$4.52 \times 10^{-9}$ |
| +1.5 < ΔΔG < +2.5 | 23 | 43 | 24 | 1.61 (0.98–2.65) | 0.06 |
| $\leq$+1.5 | 67 | 122 | 91 | 1.20 (0.92–1.58) | 0.18 |
| **$\Delta\Delta G^{PDB}$[c]** | | | | | |
| $\geq$+2.5 | 35 | 121 | 30 | *3.62 (2.43–5.40) | *$7.04 \times 10^{-10}$ |
| +1.5 < ΔΔG < +2.5 | 19 | 16 | 15 | 0.96 (0.47–1.93) | 1.08 |
| $\leq$+1.5 | 68 | 125 | 90 | 1.25 (0.95–1.63) | 0.11 |
| **BD** | | | | | |
| $\geq$0.28 | 33 | 120 | 30 | *3.59 (2.41–5.36) | *$9.25 \times 10^{-10}$ |
| 0.15 < BD < 0.28 | 19 | 54 | 30 | 1.61(1.03–2.52) | 0.04 |
| $\leq$0.15 | 70 | 88 | 75 | 1.05 (0.77–1.43) | 0.75 |

Asterisk (*) indicates statistically significant. See Figure 3 and Table S4 for further information.
[a]53,572 population-based breast cancer (BC) cases.
[b]48,048 matched controls.
[c]FoldX5.0 predictions

that, overall, PP3/BP4 evidence strengths provided by AlphaMissense, ΔΔG, and BayesDel are similar (in all cases in the moderate to strong range), but AlphaMisense outperformed ΔΔG and BayesDel in the lower proportion of missense variants with uninformative scores (PP3/BP4 not applicable). Our data are compatible with recent studies indicating that AlphaMissense correlates better with MAVE data for five genes (*DDX3X*, *BRCA1*, *MSH2*, *PTEN*, and *KCNQ4*) than earlier prediction algorithms, including BayesDel.[16]

AlphaMissense developers recommend benignity/pathogenicity default score thresholds of ≤0.34 and ≥0.56, specifying that (depending on the desired use) different gene-level cutoffs may improve trade-offs and general performance.[15] Here, we showed that AlphaMissense achieves best PP3/BP4 performance with benignity/pathogenicity score thresholds set much higher, at ≤0.65 and ≥0.75, respectively.

ΔΔG achieved the best PP3/BP4 performance with thresholds near +2 kcal/mol. This is in agreement with previous studies in other proteins indicating that ΔΔG values >+3 kcal/mol predict pathogenicity.[7,9] Interestingly, $\Delta\Delta G^{AF}$ and $\Delta\Delta G^{PDB}$ performed similarly. To what extent AlphaFold2 models can substitute experimental structures in predicting the impact of missense variants is currently a matter of debate.[31,32]

Our data on BRCA1 suggest that AlphaFold2-generated models may replace experimental PDBs in the very specific task of applying FoldX5.0-predicted ΔΔG-based PP3/BP4 computational evidence. The observation is remarkable but not necessarily true for other proteins and, in particular, for proteins lacking experimental structural data (it is possible to argue that $\Delta\Delta G^{AF}$ performs well for human BRCA1 RING and BRCT domains precisely because existing experimental data contribute to generation of high-quality AlphaFold2 models).

Perhaps more relevant is the finding that, in the subset of variants targeting the BRCT domain, $\Delta\Delta G^{AF}$ and $\Delta\Delta G^{PDB}$ performed similarly, while $\Delta\Delta G^{AF}$ outperformed $\Delta\Delta G^{PDB}$ in the subset of variants targeting the RING domain. The difference might be related to the fact that the BRCT structure PDB: 1T15 was solved by X-ray diffraction,[33] while the human BRCA1/BARD1 RING heterodimer structure PDB: 1JM7 was solved by solution NMR.[34] First, it is known that FoldX ΔΔG prediction accuracy is higher in crystallographic structures.[10] Second, on computing $\Delta\Delta G^{PDB}$ for variants targeting the RING domain, we used as template the best ranked out of 14 conformers. Perhaps the corresponding AlphaFold2 model reflects the average conformation more accurately than each individual NMR conformer, thus generating more accurate ΔΔG predictions. That said, it is worth mentioning that computing $\Delta\Delta G^{AF}$ at the RING domain was challenging and that initial attempts using a model of the BRCA1 RING monomer (equivalent to AF-P38398-F1) performed poorly (data not shown). We achieve good $\Delta\Delta G^{AF}$ PP3/BP4 performance at the RING

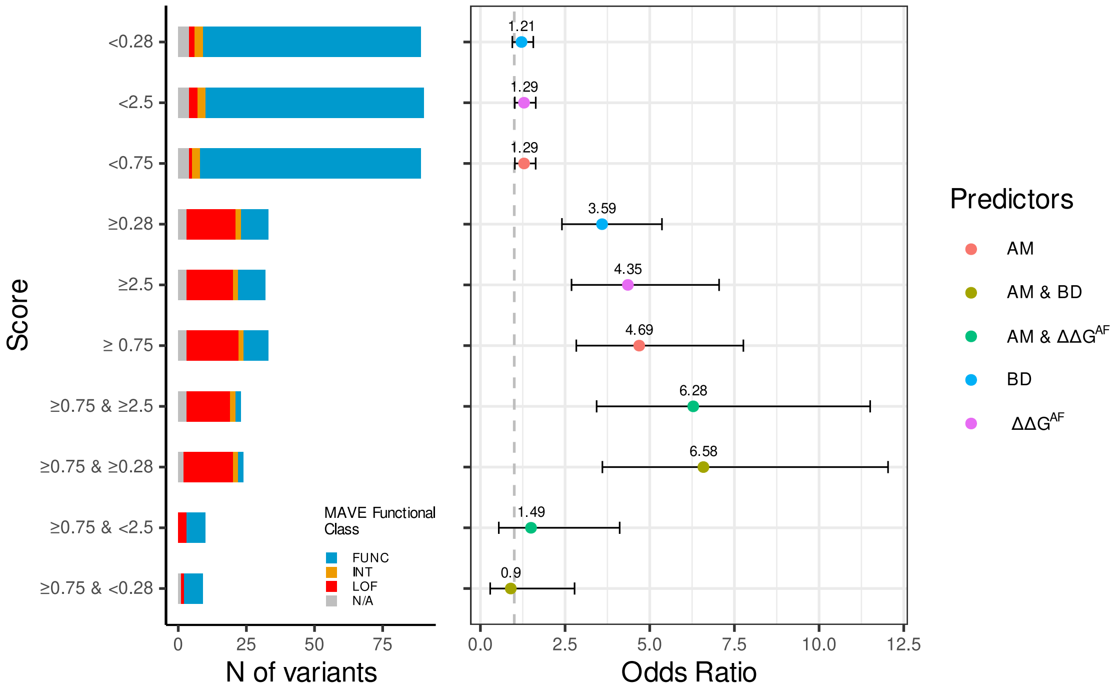


**Figure 4. BRIDGES-based breast cancer risk estimates (burden analysis) stratified by computational scores**
The plot on the right displays breast cancer ORs (and 95% confidential intervals) for variants ≥ (<) the indicated cutoff. The plot on the left shows corresponding distribution of MAVE functional classes. Note that higher ORs correspond to higher proportion of MAVE LoF variants. AM, AlphaMissense; BD, BayesDel; N/A, BRIDGES variants not assessed in MAVE.

domain only after creating a model of the BRCA1/BARD1 heterodimer that was latterly modified to introduce four critical $Zn^{2+}$ atoms (supplemental methods). In this regard, is worth mentioning that AlphaFold3 (an AlphaFold2 update that simplifies the modeling of metalloproteins and other biomolecular entities) has been released very recently.[35]

Our study points to RSA as a relevant factor to evaluate ΔΔG-based PP3/BP4 performance. Overall, the data indicate that ΔΔG provides computational pathogenicity evidence (PP3) regardless of RSA but benignity evidence (BP4) only for variants targeting buried/partially buried (RSA ≤ 60%) residues. The finding is not without a rationale. ΔΔG identifies pathogenic variants only if causing reduced thermodynamic stability, i.e., predicts benignity if stability is not impacted. Since most core pathogenic variants are likely to lead to loss of stability, the absence of destabilization is predictive of benignity for core missense variants. By contrast, surface pathogenic variants may act through protein destabilization (explaining that ΔΔG provides pathogenicity evidence) but also through alternative mechanisms such as impairing critical protein-protein interactions (without affecting folding/stability).[9,14] Consequently, absence of destabilization does not necessarily guarantee functionality for protein changes at the surface (explaining that ΔΔG does not provide benignity evidence).

In principle, AlphaMissense and BayesDel identify pathogenic variants regardless of the underlying driving mechanism, so that RSA is not an obvious factor in modulating PP3/BP4 performance. Yet we find somewhat unexpectedly that both tools, similarly to ΔΔG, are RSA modulated, and that neither of them provides computational benignity evidence (BP4) to variants targeting exposed residues. The finding probably reflects the fact that, indirectly, AlphaMissense and BayesDel (and other computational tools) capture stability-related features (unsurprisingly, since tools have been trained to discriminate pathogenic and benign missense variants and most missense pathogenic variants impact stability). At any rate, the putative relevance of RSA on evaluating PP3/BP4 computational evidence warrants further analyses in other clinically relevant proteins.

As expected, AlphaMissense shows a positive correlation with ΔΔG and BayesDel scores (Figure S6). The correlation with BayesDel is strong ($r = 0.74$), but the correlation with ΔΔG is weaker ($r = 0.46$), opening the possibility of independent AlphaMissense and ΔΔG contributions to variant classification. Indeed, we show that combined use of AlphaMissense and ΔΔG (and RSA) adds granularity to the pathogenicity and benignity evidence strengths provided by computational tools (Figures S9–S11).

Apart from increasing PP3/BP4 evidence strength granularity, the combined analysis with AlphaMissense and ΔΔG may provide additional information. For instance, we speculate that AlphaMissense and ΔΔG discordance may encapsulate relevant information contributing to the identification of INT missense variants (INT variants might be associated with AlphaMissense and ΔΔG

discordance; Figure S13). Further, AlphaMissense pathogenicity scores do not provide direct mechanistic interpretability. Combining analysis with ΔΔG may provide such interpretability, contributing to the mapping of protein regions/residues with relevant functions other than protein stability (Figures S14 and S15).

Finally, we think that our study (limited to missense variants in two functional domains of the tumor-suppressor gene *BRCA1*) illustrates some general principles and recommendations that might be relevant in evaluating PP3/BP4 computational evidence in other proteins.

(1) PP3/BP4 assessment benefits from a structure-based analysis.
(2) AlphaMissense likely outperforms earlier computational tools, but optimal gene-specific pathogenic/benign cutoffs might be very different from the generic cutoffs originally proposed.
(3) Regardless of the computational tool under assessment, we recommend a comprehensive evaluation of performance stratified by RSA (anticipating that meeting computational benignity evidence BP4 for changes at the protein surface will be challenging for many proteins).
(4) ΔΔG (FoldX5.0 predictions) adds evidence strength granularity to AlphaMissense-based computational evidence.
(5) If structural data available for the protein of interest is based on NMR, we recommend considering ΔΔG (FoldX5.0 predictions) based on an AlphaFold model as an alternative (provided that the model reflects the physiologically relevant monomeric, homo[hetero]-dimeric, or multimeric structure, and that, in the case of modeling metalloproteins, metal ions have been added).

To what extent these five principles are truly generic (applicable to other proteins), or BRCA1 specific, warrants further studies.

## Data and code availability

The datasets generated during this study are available in Table S1.

## Acknowledgments

M.d.l.H.'s research activity has been funded by Instituto de Salud Carlos III grants PI20/00110 and PI24/00267 co-funded by the European Union (ERDF/ESF, "A way to make Europe"/"Investing in your future") and a National Institutes of Health (NIH) grant 5U24CA258058-02. N.R. and A.B.-S. were supported by NHMRC funding (APP177524). C.F. was supported by funding from the National Breast Cancer Foundation, Australia (IIRS-21-102).

## Author contributions

L.R.-M.: methodology, formal analysis, investigation, data curation, writing – review & editing, and visualization. N.R.: methodology and writing – review & editing. A.E.-S.: data curation and writing – review & editing. C.F.: methodology and writing – review & editing. A.G.-S.: investigation and writing – review & editing. M.J.V.: methodology and writing – review & editing. A.C.: methodology and writing – review & editing. M.E.R.: conceptualization, writing – review & editing, and project administration. K.R.: methodology and writing – review & editing. P.P.-S.: funding acquisition and writing – review & editing. A.B.S.: conceptualization, methodology, and writing – review & editing. M.d.l.H.: conceptualization, methodology, formal analysis, writing – original draft, writing – review & editing, and funding acquisition.

## Declaration of interests

M.J.V. is an employee of Ambry Genetics. A.C. is an employee of Ambry Genetics. M.E.R. is an employee of Ambry Genetics.

## Web resources

Ensembl Variant Effect Predictor, https://www.ensembl.org/Tools/VEP
ggplot2, https://ggplot2.tidyverse.org
MedCalc statistical software, www.medcalc.org/calc
R ggpubr package, https://rpkgs.datanovia.com/ggpubr/
SVI recommendation for absence/rarity PM2—version 1.0, https://clinicalgenome.org/working-groups/sequence-variant-interpretation

## Supplemental information

Supplemental information can be found online at https://doi.org/10.1016/j.ajhg.2024.12.011.

Received: May 29, 2024
Accepted: December 12, 2024
Published: April 14, 2025

## References

1. Stein, A., Fowler, D.M., Hartmann-Petersen, R., and Lindorff-Larsen, K. (2019). Biophysical and Mechanistic Models for Disease-Causing Protein Variants. Trends Biochem. Sci. *44*, 575–588.
2. Schaafsma, G.C.P., and Vihinen, M. (2017). Large differences in proportions of harmful and benign amino acid substitutions between proteins and diseases. Hum. Mutat. *38*, 1613–1848.
3. Casadio, R., Vassura, M., Tiwari, S., Fariselli, P., and Luigi Martelli, P. (2011). Correlating disease-related mutations to their effect on protein stability: a large-scale analysis of the human proteome. Hum. Mutat. *32*, 1161–1170.
4. Pal, L.R., and Moult, J. (2015). Genetic Basis of Common Human Disease: Insight into the Role of Missense SNPs from Genome-Wide Association Studies. J. Mol. Biol. *427*, 2271–2289.
5. Petrosino, M., Novak, L., Pasquo, A., Chiaraluce, R., Turina, P., Capriotti, E., and Consalvi, V. (2021). Analysis and Interpretation of the Impact of Missense Variants in Cancer. Int. J. Mol. Sci. *22*, 5416.
6. Redler, R.L., Das, J., Diaz, J.R., and Dokholyan, N.V. (2016). Protein Destabilization as a Common Factor in Diverse Inherited Disorders. J. Mol. Evol. *82*, 11–16.

7. Nielsen, S.V., Stein, A., Dinitzen, A.B., Papaleo, E., Tatham, M.H., Poulsen, E.G., Kassem, M.M., Rasmussen, L.J., Lindorff-Larsen, K., and Hartmann-Petersen, R. (2017). Predicting the impact of Lynch syndrome-causing missense mutations from structural calculations. PLoS Genet. *13*, e1006739.
8. Reza, M.N., Ferdous, N., Emon, M.T.H., Islam, M.S., Mohiuddin, A.K.M., and Hossain, M.U. (2021). Pathogenic genetic variants from highly connected cancer susceptibility genes confer the loss of structural stability. Sci. Rep. *11*, 19264.
9. Høie, M.H., Cagiada, M., Beck Frederiksen, A.H., Stein, A., and Lindorff-Larsen, K. (2022). Predicting and interpreting large-scale mutagenesis data using analyses of protein stability and conservation. Cell Rep. *38*, 110207.
10. Schymkowitz, J., Borg, J., Stricher, F., Nys, R., Rousseau, F., and Serrano, L. (2005). The FoldX web server: an online force field. Nucleic Acids Res. *33*, W382–W388.
11. Gerasimavicius, L., Liu, X., and Marsh, J.A. (2020). Identification of pathogenic missense mutations using protein stability predictors. Sci. Rep. *10*, 15387.
12. Tunyasuvunakool, K., Adler, J., Wu, Z., Green, T., Zielinski, M., Žídek, A., Bridgland, A., Cowie, A., Meyer, C., Laydon, A., et al. (2021). Highly accurate protein structure prediction for the human proteome. Nature *596*, 590–596.
13. Jumper, J., Evans, R., Pritzel, A., Green, T., Figurnov, M., Ronneberger, O., Tunyasuvunakool, K., Bates, R., Žídek, A., Potapenko, A., et al. (2021). Highly accurate protein structure prediction with AlphaFold. Nature *596*, 583–589.
14. Akdel, M., Pires, D.E.V., Pardo, E.P., Jänes, J., Zalevsky, A.O., Mészáros, B., Bryant, P., Good, L.L., Laskowski, R.A., Pozzati, G., et al. (2022). A structural biology community assessment of AlphaFold2 applications. Nat. Struct. Mol. Biol. *29*, 1056–1067.
15. Cheng, J., Novati, G., Pan, J., Bycroft, C., Žemgulytė, A., Applebaum, T., Pritzel, A., Wong, L.H., Zielinski, M., Sargeant, T., et al. (2023). Accurate proteome-wide missense variant effect prediction with AlphaMissense. Science *381*, eadg7492.
16. Ljungdahl, A., Kohani, S., Page, N.F., Wells, E.S., Wigdor, E.M., Dong, S., and Sanders, S.J. (2023). AlphaMissense is better correlated with functional assays of missense impact than earlier prediction algorithms. Preprint at bioRxiv. https://doi.org/10.1101/2023.10.24.562294.
17. Richards, S., Aziz, N., Bale, S., Bick, D., Das, S., Gastier-Foster, J., Grody, W.W., Hegde, M., Lyon, E., Spector, E., et al. (2015). Standards and guidelines for the interpretation of sequence variants: a joint consensus recommendation of the American College of Medical Genetics and Genomics and the Association for Molecular Pathology. Genet. Med. *17*, 405–424.
18. Tian, Y., Pesaran, T., Chamberlin, A., Fenwick, R.B., Li, S., Gau, C.L., Chao, E.C., Lu, H.M., Black, M.H., and Qian, D. (2019). REVEL and BayesDel outperform other in silico meta-predictors for clinical variant classification. Sci. Rep. *9*, 12752.
19. Pejaver, V., Byrne, A.B., Feng, B.J., Pagel, K.A., Mooney, S.D., Karchin, R., O'Donnell-Luria, A., Harrison, S.M., Tavtigian, S.V., Greenblatt, M.S., et al. (2022). Calibration of computational tools for missense variant pathogenicity classification and ClinGen recommendations for PP3/BP4 criteria. Am. J. Hum. Genet. *109*, 2163–2177.
20. Parsons, M.T., de la Hoya, M., Richardson, M.E., Tudini, E., Anderson, M., Berkofsky-Fessler, W., Caputo, S.M., Chan, R.C., Cline, M.S., Feng, B.J., et al. (2024). Evidence-based recommendations for gene-specific ACMG/AMP variant classification from the ClinGen ENIGMA BRCA1 and BRCA2 Variant Curation Expert Panel. Am. J. Hum. Genet. *9*, S0002–S9297.
21. Feng, B.J. (2017). PERCH: A Unified Framework for Disease Gene Prioritization. Hum. Mutat. *38*, 243–251.
22. Findlay, G.M., Daza, R.M., Martin, B., Zhang, M.D., Leith, A.P., Gasperini, M., Janizek, J.D., Huang, X., Starita, L.M., and Shendure, J. (2018). Accurate classification of BRCA1 variants with saturation genome editing. Nature *562*, 217–222.
23. Mizuguchi, K., Deane, C.M., Blundell, T.L., Johnson, M.S., and Overington, J.P. (1998). JOY: protein sequence-structure representation and analysis. Bioinformatics *14*, 617–623.
24. McLaren, W., Gil, L., Hunt, S.E., Riat, H.S., Ritchie, G.R.S., Thormann, A., Flicek, P., and Cunningham, F. (2016). The Ensembl Variant Effect Predictor. Genome Biol. *17*, 122.
25. Sing, T., Sander, O., Beerenwinkel, N., and Lengauer, T. (2005). ROCR: visualizing classifier performance in R. Bioinformatics *21*, 3940–3941.
26. Robin, X., Turck, N., Hainard, A., Tiberti, N., Lisacek, F., Sanchez, J.C., and Müller, M. (2011). pROC: an open-source package for R and S+ to analyze and compare ROC curves. BMC Bioinf. *12*, 77.
27. Tavtigian, S.V., Greenblatt, M.S., Harrison, S.M., Nussbaum, R.L., Prabhu, S.A., Boucher, K.M., Biesecker, L.G.; and ClinGen Sequence Variant Interpretation Working Group ClinGen SVI (2018). Modeling the ACMG/AMP variant classification guidelines as a Bayesian classification framework. Genet. Med. *20*, 1054–1060.
28. Tavtigian, S.V., Harrison, S.M., Boucher, K.M., and Biesecker, L.G. (2020). Fitting a naturally scaled point system to the ACMG/AMP variant classification guidelines. Hum. Mutat. *41*, 1734–1737.
29. Dorling, L., Carvalho, S., Allen, J., González-Neira, A., Luccarini, C., Wahlström, C., Pooley, K.A., Parsons, M.T., Fortuno, C., et al.; Breast Cancer Association Consortium (2021). Breast Cancer Risk Genes - Association Analysis in More than 113,000 Women. N. Engl. J. Med. *384*, 428–439.
30. Spurdle, A.B., Greville-Heygate, S., Antoniou, A.C., Brown, M., Burke, L., de la Hoya, M., Domchek, S., Dörk, T., Firth, H.V., Monteiro, A.N., et al. (2019). Towards controlled terminology for reporting germline cancer susceptibility variants: an ENIGMA report. J. Med. Genet. *56*, 347–357.
31. Buel, G.R., and Walters, K.J. (2022). Can AlphaFold2 predict the impact of missense mutations on structure? Nat. Struct. Mol. Biol. *29*, 1–2.
32. Pak, M.A., Markhieva, K.A., Novikova, M.S., Petrov, D.S., Vorobyev, I.S., Maksimova, E.S., Kondrashov, F.A., and Ivankov, D.N. (2023). Using AlphaFold to predict the impact of single mutations on protein stability and function. PLoS One *18*, e0282689.
33. Clapperton, J.A., Manke, I.A., Lowery, D.M., Ho, T., Haire, L.F., Yaffe, M.B., and Smerdon, S.J. (2004). Structure and mechanism of BRCA1 BRCT domain recognition of phosphorylated BACH1 with implications for cancer. Nat. Struct. Mol. Biol. *11*, 512–518.
34. Brzovic, P.S., Rajagopal, P., Hoyt, D.W., King, M.C., and Klevit, R.E. (2001). Structure of a BRCA1-BARD1 heterodimeric RING-RING complex. Nat. Struct. Biol. *8*, 833–837.
35. Abramson, J., Adler, J., Dunger, J., Evans, R., Green, T., Pritzel, A., Ronneberger, O., Willmore, L., Ballard, A.J., Bambrick, J., et al. (2024). Accurate structure prediction of biomolecular interactions with AlphaFold 3. Nature *636*, E4.

The American Journal of Human Genetics, Volume *112*

## Supplemental information

# ACMG/AMP interpretation of *BRCA1* missense variants: Structure-informed scores add evidence strength granularity to the PP3/BP4 computational evidence

**Lobna Ramadane-Morchadi, Nitsan Rotenberg, Ada Esteban-Sánchez, Cristina Fortuno, Alicia Gómez-Sanz, Matthew J. Varga, Adam Chamberlin, Marcy E. Richardson, Kyriaki Michailidou, Pedro Pérez-Segura, Amanda B. Spurdle, and Miguel de la Hoya**

## Supplemental Figures

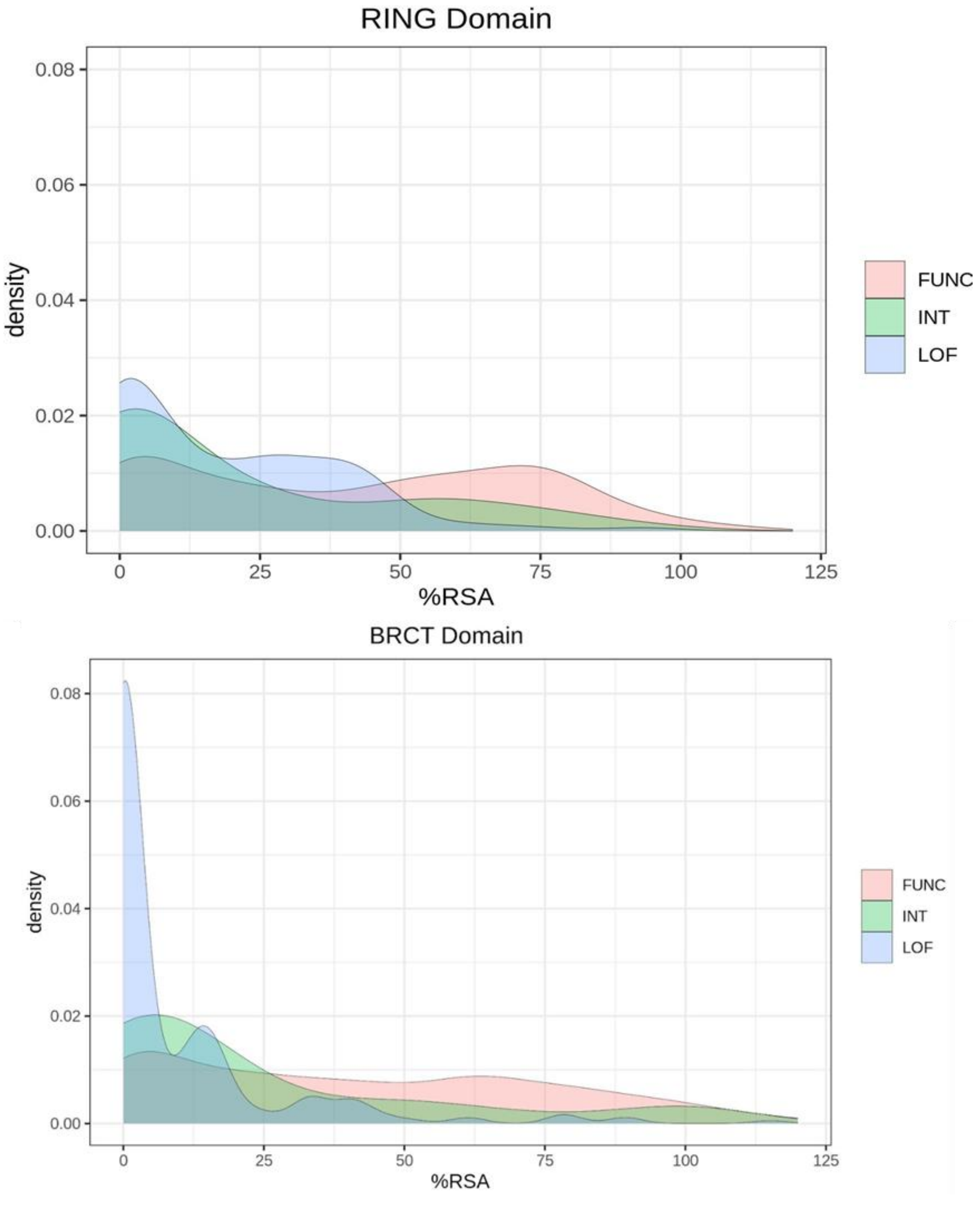


**Figure S1A.** The density plots show for the RING (top) and BRCT (bottom) domains, the distribution of residue solvent accessibility (%RSA) stratified by MAVE functional class. Residues targeted by functional (FUNC), intermediate (INT), and loss-of-function (LOF) variants are shown. Overall, INT and LOF variants tend to target buried residues. This trend is particularly striking for LOF variants targeting BRCT residues.

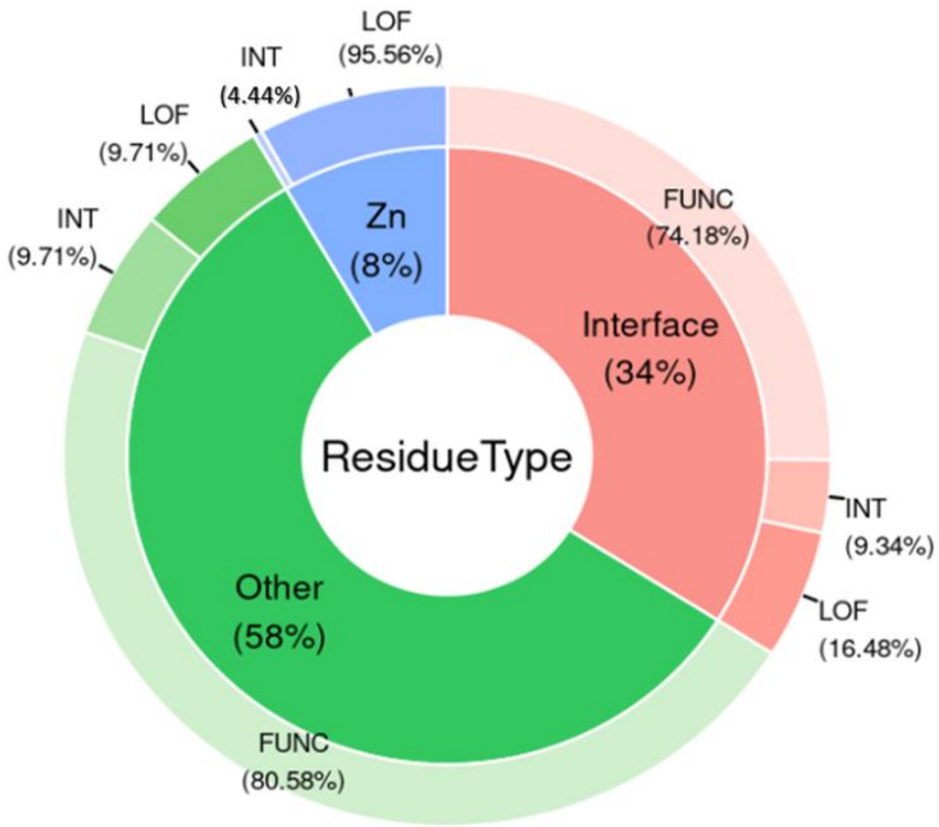


**Figure S1B.** The donut plot shows MAVE functional categories stratified by residue subtype in the BRCA1 RING domain. The inner circle shows RING residues manually stratified into the following subtypes: Zn-interacting, located in the interface with BARD1, and others. The external circle shows the proportion of MAVE functional categories per residue subtype. Essentially, all variants targeting Zn-interacting residues are LOF. The proportion of LOF variants targeting interface residues is higher than the proportion of LOF variants targeting other residues (16.5% vs. 9.7%). Interestingly, the proportion of INT variants targeting interface or other residues is similar.

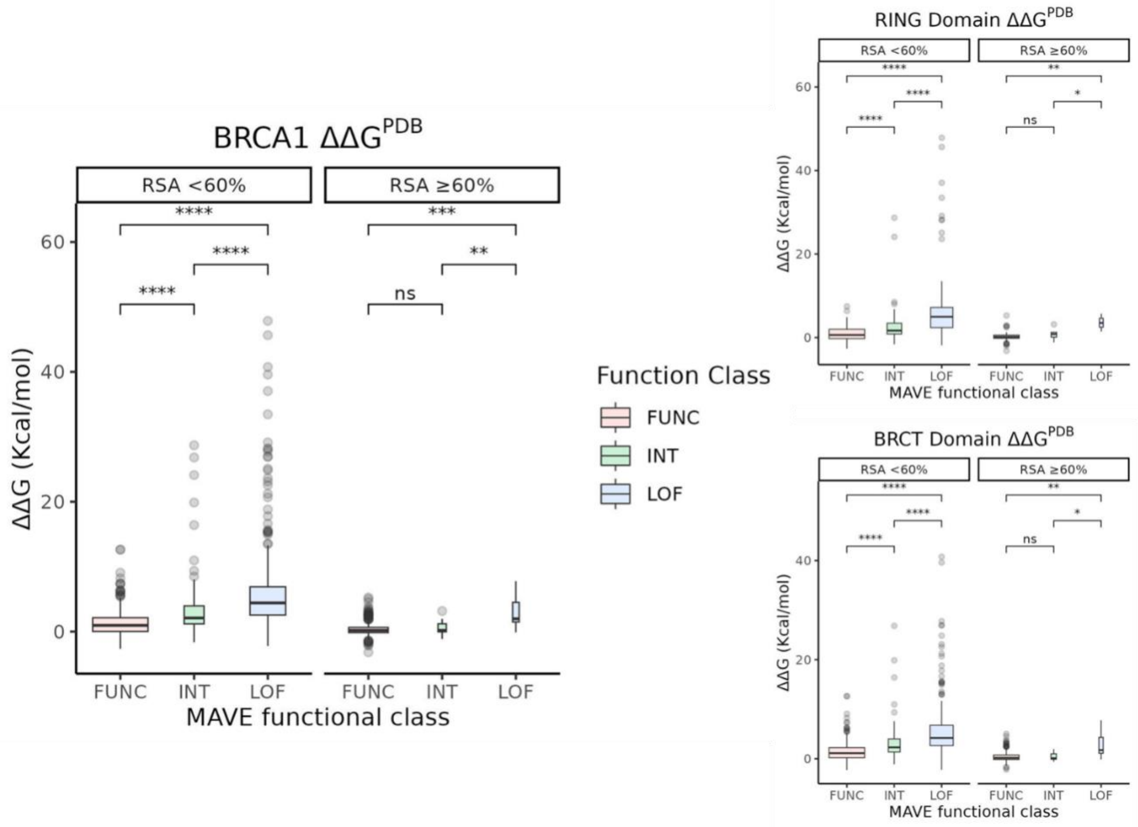


**Figure S2.** The boxplots show $\Delta\Delta G^{PDB}$ distribution *per* MAVE functional class. Left panel shows stratification by RSA into buried-partially buried (RSA ≤60%) and exposed (RSA>60%) residues. Right panel shows additional stratification by functional domain into RING (top) and BRCT (bottom) variants.Box sizes are proportional to the number of variants (N) in each class. Upper and lower box hinges correspond to Q1 and Q3 (25% and 75% percentiles), while the upper and lower whiskers extends to +/- 1.5*IQR (interquartile range). Dots correspond to outlier values**.** Wilcoxon test p-values for pair comparisons are represented as: ns for p> 0.05 (non-significant),* p≤ 0.05, ** p≤ 0.01, *** p≤ 0.001 and **** p≤ 0.0001.

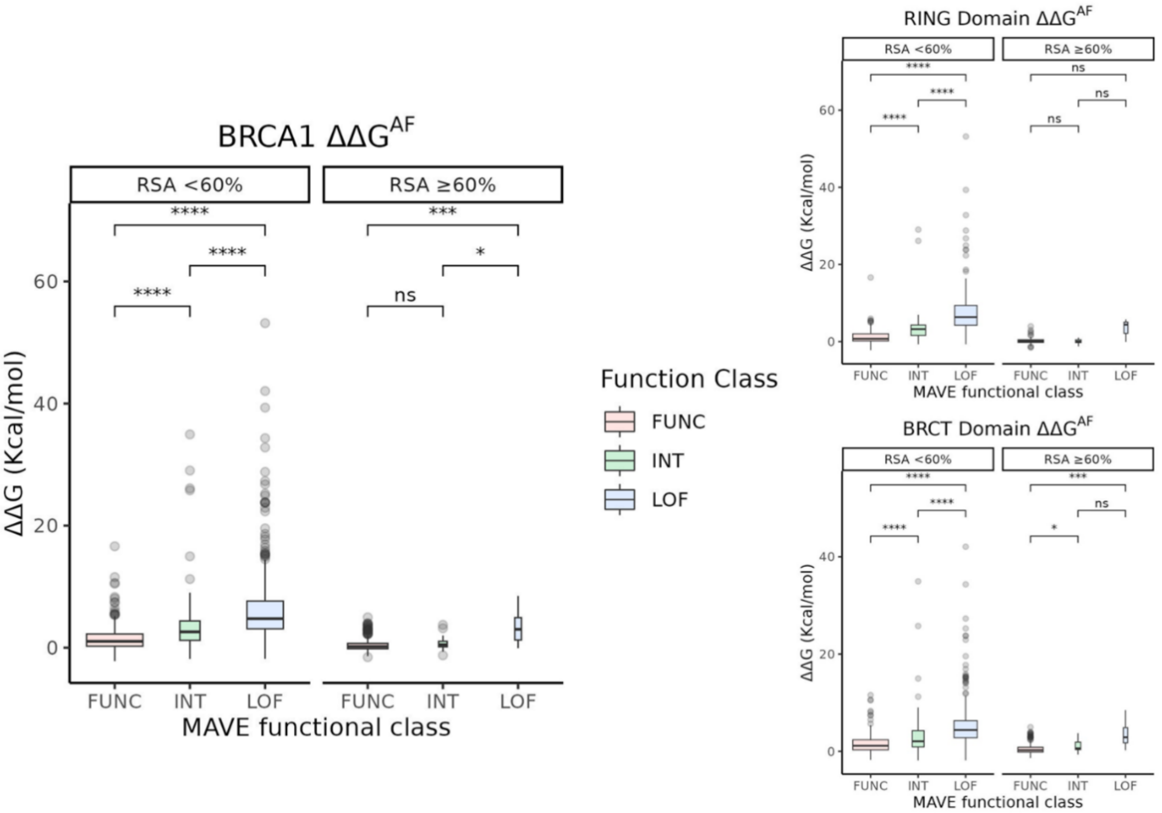


**Figure S3.** The boxplots show **ΔΔG$^{AF}$** distribution *per* MAVE functional class. Left panel shows stratification by RSA into buried/partially buried (RSA ≤60%) and exposed (RSA>60%) residues. Right panel shows additional stratification by functional domain into RING (upper) and BRCT (bottom) variants.Box sizes are proportional to the number of variants (N) in each class. Upper and lower box hinges correspond to Q1 and Q3 (25% and 75% percentiles), while the upper and lower whiskers extends to +/- 1.5*IQR (interquartile range). Dots correspond to outlier values**.** Wilcoxon test p-values for pair comparisons are represented as: ns for p> 0.05 (non-significant),* p≤ 0.05, ** p≤ 0.01, *** p≤ 0.001 and **** p≤ 0.0001.

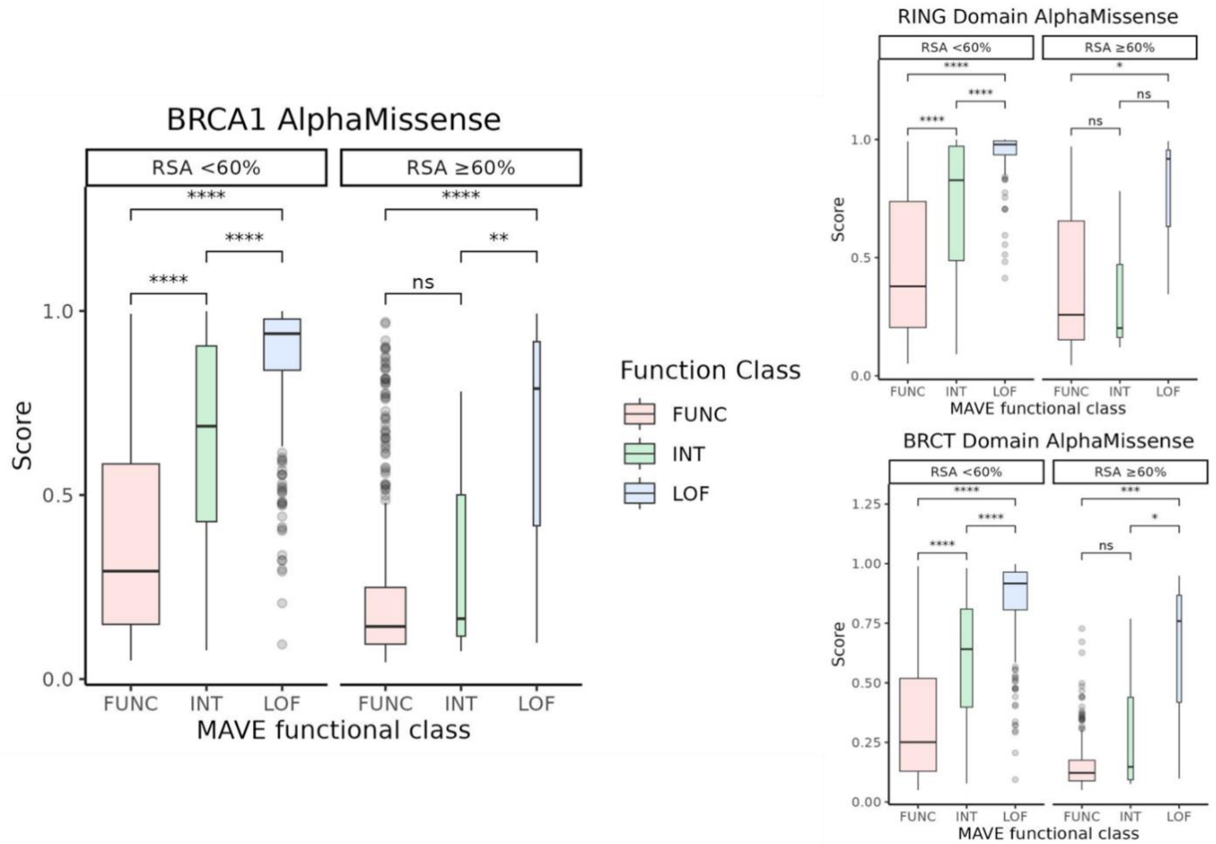


**Figure S4.** The boxplots show **AlphaMissense** pathogenicity score distribution *per* MAVE functional class. Left panel shows stratification by RSA into buried/partially buried (RSA ≤60%) and exposed (RSA>60%) residues. Right panel shows stratification by functional domain into RING (upper) and BRCT (bottom) variants.Box sizes are proportional to the number of variants (N) in each class. Upper and lower box hinges correspond to Q1 and Q3 (25% and 75% percentiles), while the upper and lower whiskers extends to +/- 1.5*IQR (interquartile range). Dots correspond to outlier values**.** Wilcoxon p-values for pair comparisons are represented as: ns for p> 0.05 (non-significant),*  p≤ 0.05, ** p≤ 0.01, *** p≤ 0.001 and **** p≤ 0.0001.

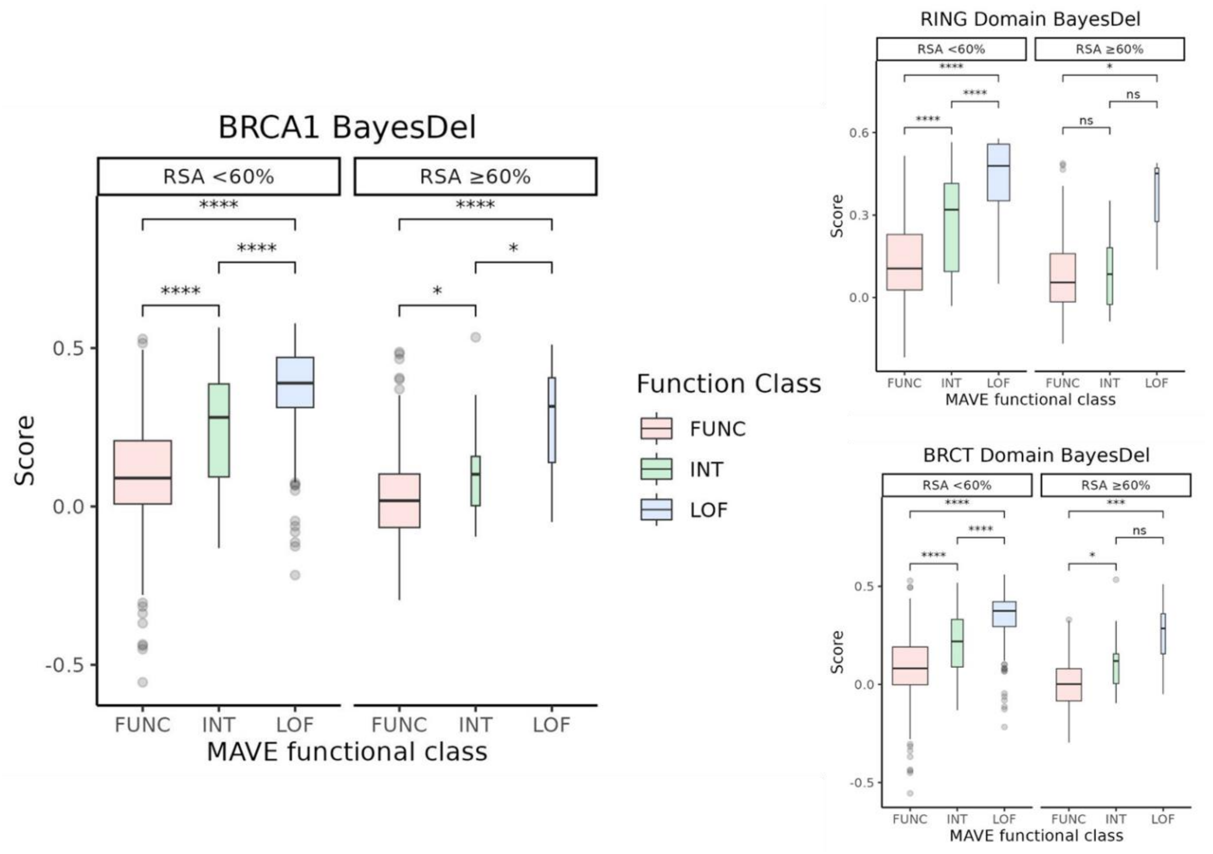


**Figure S5.** The boxplots show **BayesDel** score distribution *per* MAVE functional class. Left panel shows stratification by RSA into buried/partially buried (RSA ≤60%) and exposed (RSA>60%) residues. Right panel shows additional stratification by functional domain into RING (top) and BRCT (bottom) variants.Box sizes are proportional to the number of variants (N) in each class. Upper and lower box hinges correspond to Q1 and Q3 (25% and 75% percentiles), while the upper and lower whiskers extends to +/- 1.5*IQR (interquartile range). Dots correspond to outlier values**.** Wilcoxon p-values for pair comparisons are represented as: ns for p> 0.05 (non-significant),* p≤ 0.05, ** p≤ 0.01, *** p≤ 0.001 and **** p≤ 0.0001.

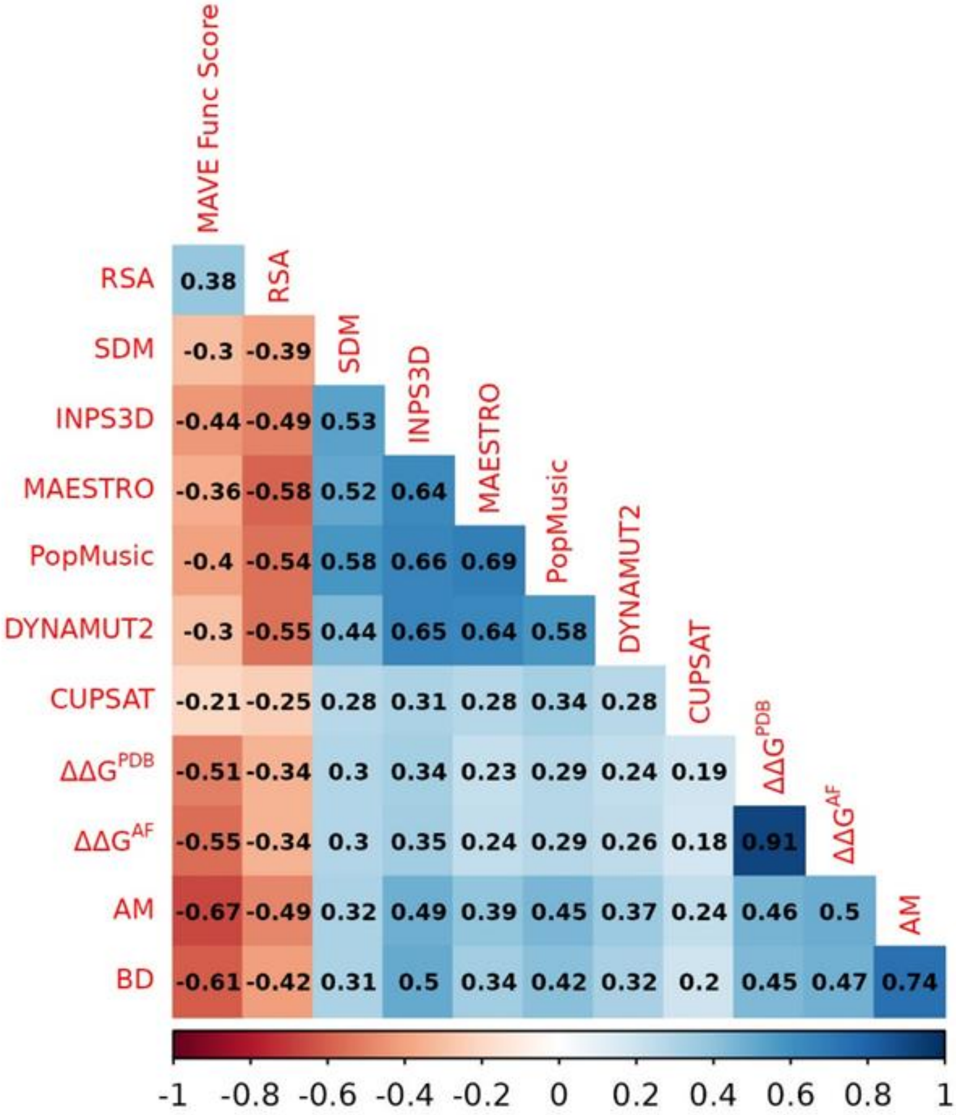


**Figure S6.** The plot summarizes the linear dependence (Pearson correlation coefficient **r**) between pairs of variables analysed in the present study. Note that correlation between FoldX5.0 ΔΔG predictions using experimental or AF2 templates (**$\Delta\Delta G^{PDB}$** vs. **$\Delta\Delta G^{AF}$)** is very high (r=0.91), but correlation between FoldX5.0 and web-based ΔΔG predictions is much lower (**r** ranging from 0.18 to 0.35). For the present correlation analysis, MAVE functional scores include INT variants. **RSA** (Residue solvent accessibility). **AM** (AlphaMissense). **BD** (BayesDel). **SDM, INPS3D, MAESTRO, PopMusic, DYNAMUT2, CUPSAT** (web-based ΔΔG predictors).

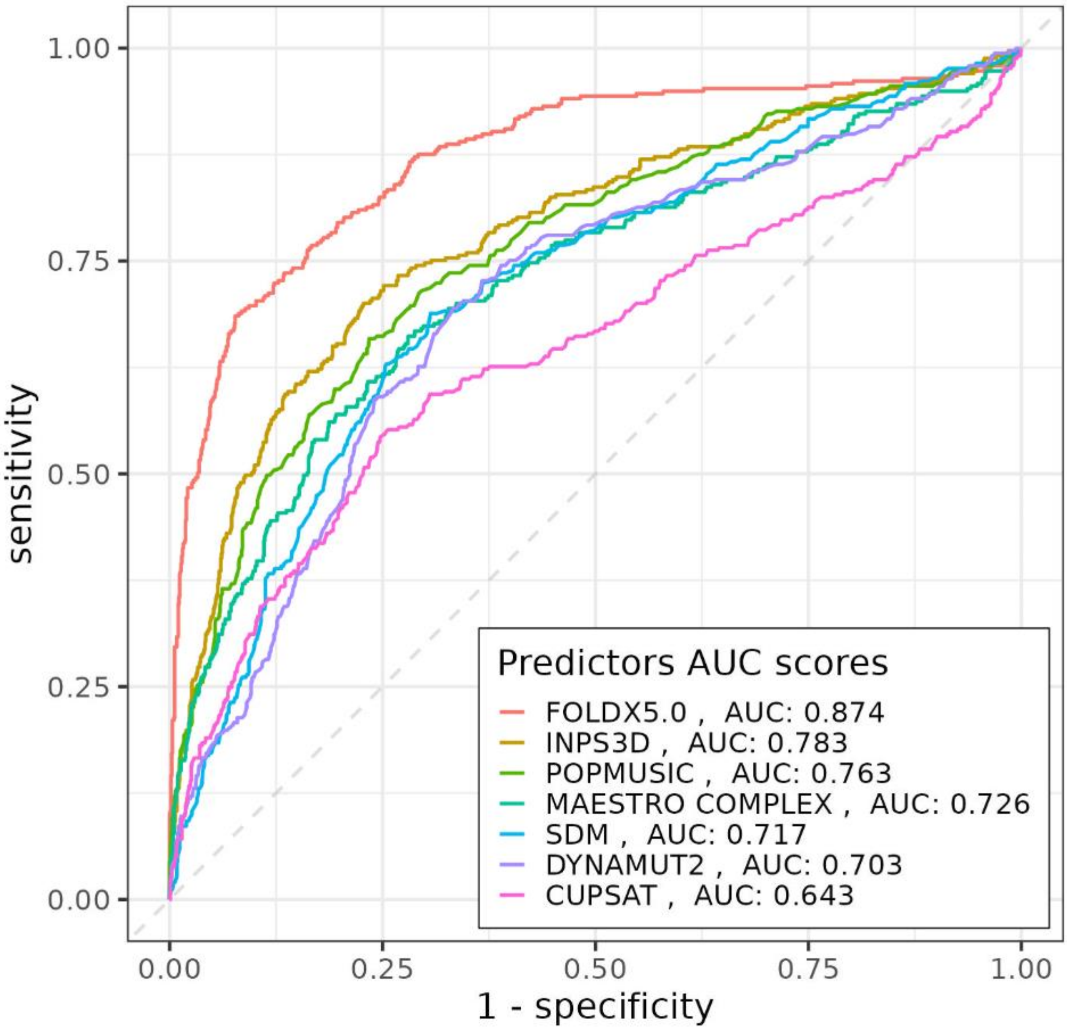


**Figure S7.** Analysis of FoldX5.0 $\Delta\Delta G^{PDB}$ and six web-based ΔΔG predictors (SDM, INPS3D, POPMUSIC, DYNAMUT, MAESTRO and CUPSAT) at discriminating LOF and FUNC variants at the RING and BRCT domains (MAVE dataset with INT variants filtered-out). For each predictor, a ROC plot and the corresponding auROC value are displayed. Overall, FoldX5.0 $\Delta\Delta G^{PDB}$ outperforms web-based programs. Delong's test shows a statistically significant difference between $\Delta\Delta G^{PDB}$ and INPS3D, the best web-based predictor (p-value = $3.35\times10^{-10}$). We performed an alternative analysis in which ΔΔG-stability was replaced by ΔΔG-interaction in the subset of variants targeting the BRCA1-BARD1 heterodimer interface, but no improvement on auROC was observed (data not shown).

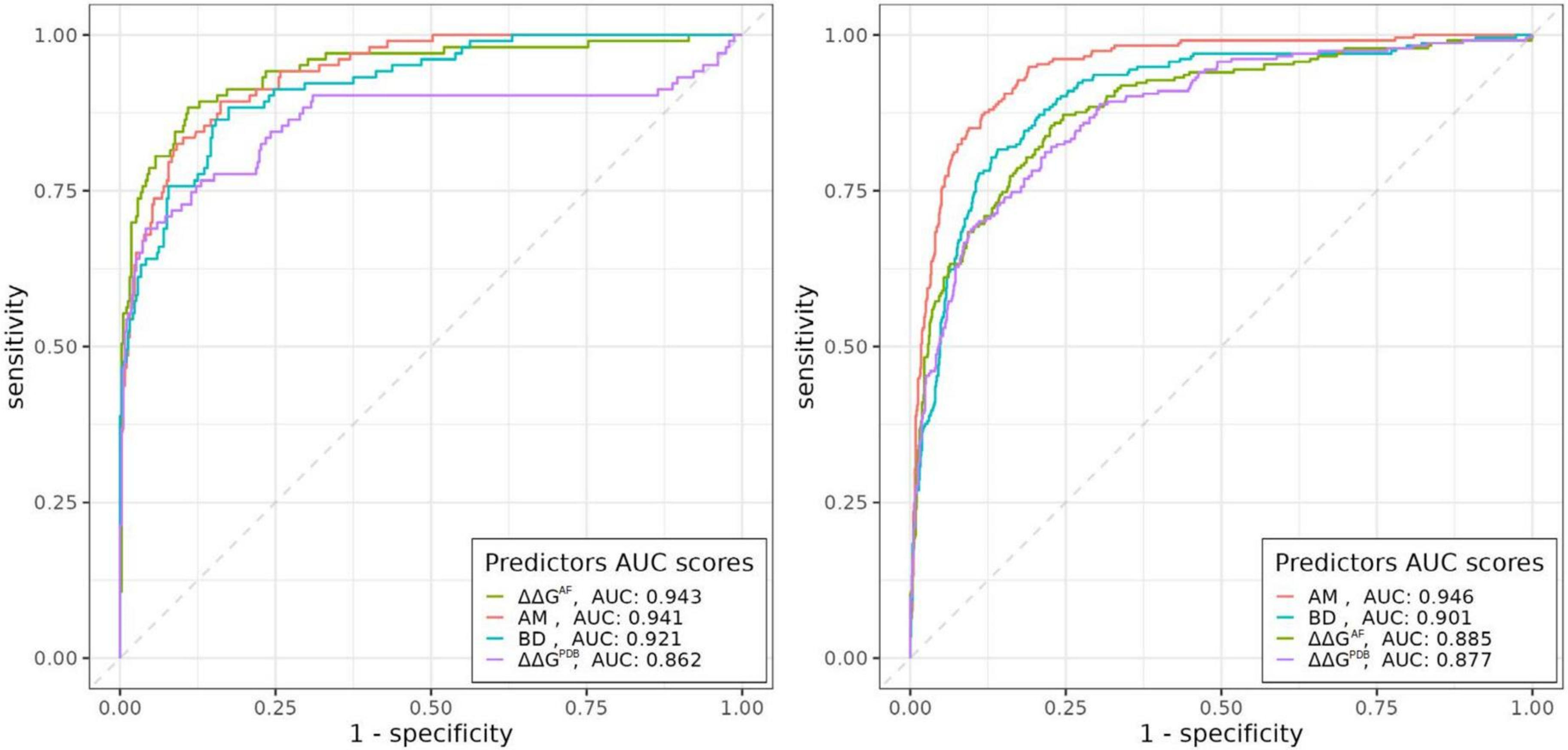


**Figure S8.** Analysis of AM**,** $\Delta\Delta G^{PDB}$, $\Delta\Delta G^{AF}$, and BayesDel performance at discriminating LOF and FUNC variants (MAVE dataset with INT variants filtered-out) at the RING (left panel) and BRCT domains (right panel). For each predictor, a ROC plot and the corresponding auROC value are displayed. Overall, $\Delta\Delta G^{AF}$ provides the best discrimination at the RING domain, while AM provides the best discrimination at the BRCT domain. Interestingly, $\Delta\Delta G^{AF}$ outperforms $\Delta\Delta G^{PDB}$, in particular at the RING domain (AUC 0.943 vs. 0.862). See text for further details.

| | BD-based PP3/BP4 | | | AM-based PP3/BP4 | | | (AM+$\Delta\Delta G^{AF}$)-based PP3/BP4 | | |
|---|---|---|---|---|---|---|---|---|---|
| **score** | **<=.15** | **><** | **>=.28** | **<=.65** | **><** | **>=.75** | **<=.65<=1.5** | **><** | **>=.75>=2.5** |
| **Log2 LR** | -2.9 | -0.5 | +2.6 | -2.9 | ** | +2.8 | -4.5 | -0.5 | +4.2 |
| **N** | 883 | 216 | 420 | 1015 | 78 | 426 | 748 | 478 | 293 |
| **LR-based strength** | **BP4_M** | n/a | **PP3_M** | **BP4_M** | n/a | **PP3_M** | **BP4_S** | n/a | **PP3_S** |
| **Recommended strength** | **BP4** | n/a | **PP3** | **BP4** | n/a | **PP3** | **BP4_M** | n/a | **PP3_M** |

**Figure S9. BayesDel, AlphaMissense, and (AlphaMissense+ΔΔG)-based PP3/BP4 computational evidence.** Our study indicates that the BayesDel-based PP3/BP4 computational evidence (**BD-based PP3/BP4**) currently recommended by the *ClinGen ENIGMA BRCA1 and BRCA2 Variant Curation Expert Panel* (VCEP) for *BRCA1* missense variants at the RING and BRCA domains provides pathogenicity and benignity evidence with **LR-based strength** in the moderate to strong range (±2<Log2 LR<±4). This is in agreement with previous studies by the VCEP estimating at least moderate evidence for BayesDel. However, the VCEP opted, conservatively, to downgrade the evidence strength one level, applying the ACMG/AMP baseline supporting. Similarly, here we recommend downgrading evidence strength one level (**recommended strength**). An AlphaMissense-based PP3/BP4 computational evidence (**AM-based PP3/BP4**) is superior in that provides very similar evidence strengths to more variants (in our MAVE cohort of 337 LOF and 1182 FUNC variants, 216 variants have no PP3/BP4 evidence applicable with BD, while only 78 variants have no PP3/BP4 evidence applicable with AlphaMissense). A ΔΔG-based computational evidence performs very similar to BayesDel (**see Figure 2**). Concordant AlphaMissense and $\Delta\Delta G^{AF}$ scoring [**(AM+ $\Delta\Delta G^{AF}$)-based PP3/BP4**] provides BP4 and PP3 computational evidences with strong strengths (Log2 LRs -4.5 and +4.2, respectively), but the proportion of variants with no PP3/BP4 evidence strength applicable is higher (478 MAVE cohort variants have no PP3/BP4 evidence applicable with this combined approach). For clarity, ACMG/AMP Strong (S), moderate (M) and supporting (P) evidences are color-coded (green for benignity and brown for pathogenicity). (><) scoring in the uncertain zone between benignity and pathogenicity thresholds. (**) No statistically significant benign or pathogenic evidence (Log2 LR 95%CI includes 0). (n/a) not applicable. (**BD**) BayesDel. (**AM**) AlphaMissense. (**$\Delta\Delta G^{AF}$**) FoldX5.0 ΔΔG predictions using AlphaFold2 PDB templates. **(><)** Bioinformatic code not applicable (**N**) MAVE dataset variants in each scoring category.

| | (AM+ΔΔG$^{AF}$)-based PP3/BP4 | | | | | |
|---|---|---|---|---|---|---|
| RSA score | <=60% | | | >60% | | |
| N | 1109 | | | 410 | | |
| AM + ΔΔG score | <=.65<=1.5 | >< | >=.75>=2.5 | <=.65<1.5 | >< | >=.75>=2.5 |
| Log2 LR | -4.9 | -0.7 | +3.7 | -1.6* | ** | +7.5 |
| N | 426 | 396 | 287 | 322 | 82 | 6 |
| LR-based strength | BP4_S | n/a | PP3_M | BP4 | n/a | PP3_S |
| Recommended strength | BP4_M | n/a | PP3 | n/a | n/a | PP3_M |

**Figure S10. Impact of RSA in (AlphaMissense+ΔΔG$^{AF}$)-based PP3/BP4 computational evidence**. In the subgroup of variants targeting exposed residues (RSA>60%), the approach did not provide statistically significant benignity evidence of supporting strength (**Log2 LR=-1.55, 95%CI -2.94 to -0.16**). For clarity, ACMG/AMP strong (S), moderate (M) and supporting (P) evidence strengths are color-coded green (benignity) and brown (pathogenicity). Following *ClinGen ENIGMA BRCA1 and BRCA2 Variant Curation Expert Panel* recommendations, we distinguish LR-based and recommended (conservative instance) strengths. (*****) No statistically significant benignity evidence (Log2 LR 95% CI overlaps -1). (******) No statistically significant benignity or pathogenicity evidence (Log2 LR 95% CI overlaps 0). **(RSA)** Residue Solvent Accessibility. **(AM)** AlphaMissense. **(><)** Bioinformatic code not applicable. **(N)** MAVE dataset variants in each score category.

| | | | | | | | | | | | | | | |
|---|---|---|---|---|---|---|---|---|---|---|---|---|---|---|
| | **(RSA/AM/$\Delta\Delta G^{AF}$)-based PP3/BP4** | | | | | | | | | | | | | |
| **RSA score** | | | | **<=60%** | | | | | | | **>60%** | | | |
| **N** | | | | **1109** | | | | | | | **410** | | | |
| **AM score** | | **<=.65** | | **><** | | **>=.75** | | | **<=.65** | | **><** | | **>=.75** | |
| **Log2 LR** | | -3.0 | | | | +2.5 | | | -1.3* | | | | +3.1 | |
| **N** | | 649 | | 66 | | 394 | | | 366 | | 12 | | 32 | |
| **ΔΔG score** | **<=1.5** | **><** | **>=3.5** | | **<=1.5** | **><** | **>=3.5** | **<=0** | **><** | **>=2** | | **<=0** | **><** | **>=2** |
| **Log2 LR** | -1.9 | | +2.4 | | -2.5 | | +1.7 | -0.6** | | +1.9** | | -2.1** | | +3.5 |
| **N** | 426 | 167 | 56 | 66 | 63 | 97 | 234 | 139 | 203 | 24 | 12 | 8 | 16 | 8 |
| **Log2 LR + Log2 LR** | -4.9 | -3.0 | -0.6 | n/a | 0 | +2.5 | +4.2 | | -1.3* | | n/a | +3.1 | | +6.6 |
| **Evidence Strength:** | | | | | | | | | | | | | | |
| **LR-based** | **BP4_S** | **BP4_M** | | **n/a** | | **PP3_M** | **PP3_S** | | **n/a** | | | **PP3_M** | | **PP3_S** |
| **Recommended** | **BP4_M** | **BP4** | | **n/a** | | **PP3** | **PP3_M** | | **n/a** | | | **PP3** | | **PP3_M** |
| **N** | 426 | 167 | | 185 | | 97 | 234 | | 378 | | | 24 | | 8 |

**Figure S11. Cascade stratification by RSA, AM and ΔΔG provide PP3/BP4 evidence strength granularity.** We first stratified 1519 MAVE functional scores by **RSA** (60% cut-off) into variants targeting buried-exposed residues (N=1109) and variants targeting very exposed residues (N=410). We next stratified by **AM** score applying the indicated benignity (<=0.65) and pathogenicity (>=0.75) thresholds. Evidence strengths (Log2 LR) provided by **AM** are in the moderate to strong range (2<Log2 LR<4), except for benignity evidence in the very exposed subgroup (N=366), that reaches supporting strength only (Log2 LR=-1.3). We next stratified by **$\Delta\Delta G^{AF}$** (FoldX5.0 prediction) using different benignity and pathogenicity thresholds depending on **RSA**. In the (RSA<=60+AM<=0.65)-subgroup of variants (N=649), **$\Delta\Delta G^{AF}$** provides additional benignity evidence with near moderate strength (Log2 LR=-1.9) to 426 variants and, remarkably, pathogenicity evidence with strength in the moderate to strong range (Log2 LR=+2.4) to 56 variants. Similarly, **$\Delta\Delta G^{AF}$** provides benignity and pathogenicity evidences to the (RSA<=60+AM>=0.75)- and (RSA>60+AM>=0.75)-subgroups of variants. We next combine **AM** and **$\Delta\Delta G^{AF}$** evidence strengths (Lo2 LR + Log2 LR) in each subgroup of variants, and transform Log2 LR values into **ACMG/AMP** Strong (**S**), Moderate (**M**), Supporting (**P**), and no evidence strength (see methods). For clarity, benignity and pathogenicity evidence strengths are color-coded (green and brown palettes, respectively).(**N**) MAVE variants in each subgroup. (**n/a**) not applicable. (**><**) MAVE variants in the no evidence score range. (*****) Log2 LRs 95 CI overlaps -1. (******) Log2 LRs 95 CI overlaps zero.

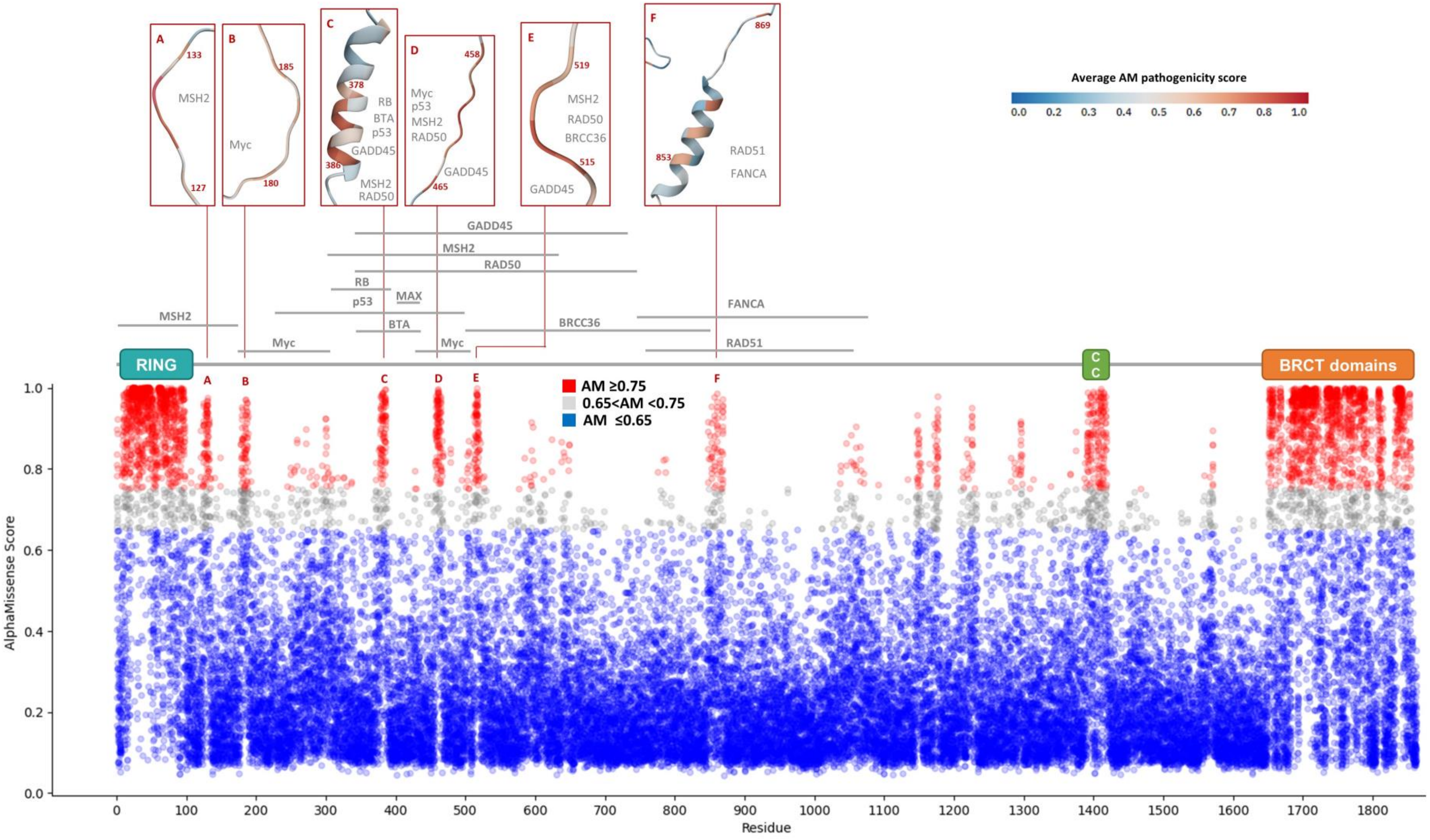

A
133
MSH2
127
B
185
Myc
180
C
378
RB
BTA
p53
GADD45
386
MSH2
RAD50
D
458
Myc
p53
MSH2
RAD50
GADD45
465
E
519
MSH2
RAD50
BRCC36
515
GADD45
F
869
853
RAD51
FANCA
Average AM pathogenicity score
0.0 0.2 0.3 0.4 0.5 0.6 0.7 0.8 1.0
GADD45
MSH2
RAD50
RB
p53
MAX
BTA
MSH2
Myc
Myc
BRCC36
FANCA
RAD51
RING
C C
BRCT domains
AM ≥0.75
0.65<AM <0.75
AM ≤0.65
AlphaMissense Score
Residue

**Figure S12**. The bottom plot (generated with Matplotlib) shows a graphical representation of AlphaMissense (AM) pathogenicity scores for each of the 19 possible substitutions at each of the 1863 BRCA1 residues. BRCA1-specific cut-offs for pathogenicity/benignity evidence are color-coded red/blue as indicated (no evidence color-coded grey). The plot shows hot AM clusters (pathogenicity scores enriched/benignity scores depleted) at the **RING** (residues 2-103), **CC** (residues 1391-1424), and **BRCT** (residues 1650-1857) domains, while the rest of the protein (mostly intrinsically disordered regions) show a very low rate of pathogenicity scores. Overall, this is in agreement with *expert panel* ACMG/AMP criteria specifications that apply PP3/BP4 computational evidence to missense variants targeting RING, CC and BRCT residues only, applying BP1_Strong to missense variants targeting other residues. Yet, the plot shows as well hot AM clusters spanning residues 127-133 (**A**), 180-185 (**B**), 378-386 (**C**), 458-465 (**D**), 515-519 (**E**) and 857-869 (**F**), suggesting that these BRCA1 regions might be relevant, and that BP1_Strong might not be warranted for missense variants targeting these hot AM clusters. Unfortunately, the scarcity of missense variants targeting the hot AM clusters in the BRIDGES dataset did not allow us to perform any clinical validation. As far as we know, no high quality BRCA1 functional annotation maps to any of these regions (Uniprot P38398, feature viewer, last access 22/08/2024). However, we hypothesize that these hot AM clusters might be functionally and clinically relevant, as they overlap BRCA1 regions with *in vitro* data supporting binding to partners proteins (1–4). Data is summarized in the central part of the figure. Cluster A (disordered region) overlaps a **MSH2** binding region (residues 1-175). Cluster B (disordered region) overlaps a **Myc** binding region (175-303). Cluster C (α–helix) overlaps **p53** (224-500), **Rb** (304-394), **RAD50** (341-758), **MSH2** (303-625), and **GADD45** (341-748) binding regions, as well as the BRCA1 basal transcriptional activation (**BTA**) region (4). Cluster D (disordered region) overlaps **Myc** (433-511), **p53** (224-500), **RAD50** (341-758), **MSH2** (303-625), and **GADD45** (341-748) binding regions. Cluster E (disordered region) overlaps **BRCC36** (502-852), **RAD50** (341-758), **MSH2** (303-625), and **GADD45** (341-748) binding regions. Finally, Cluster F (α–helix plus disordered region) overlaps **RAD51** (758-1064) and **FANCA** (740-1083) regions. For clarity, we have indicated BRCA1 interactions potentially disturbed by missense changes at the hot AM clusters in the corresponding top panels.

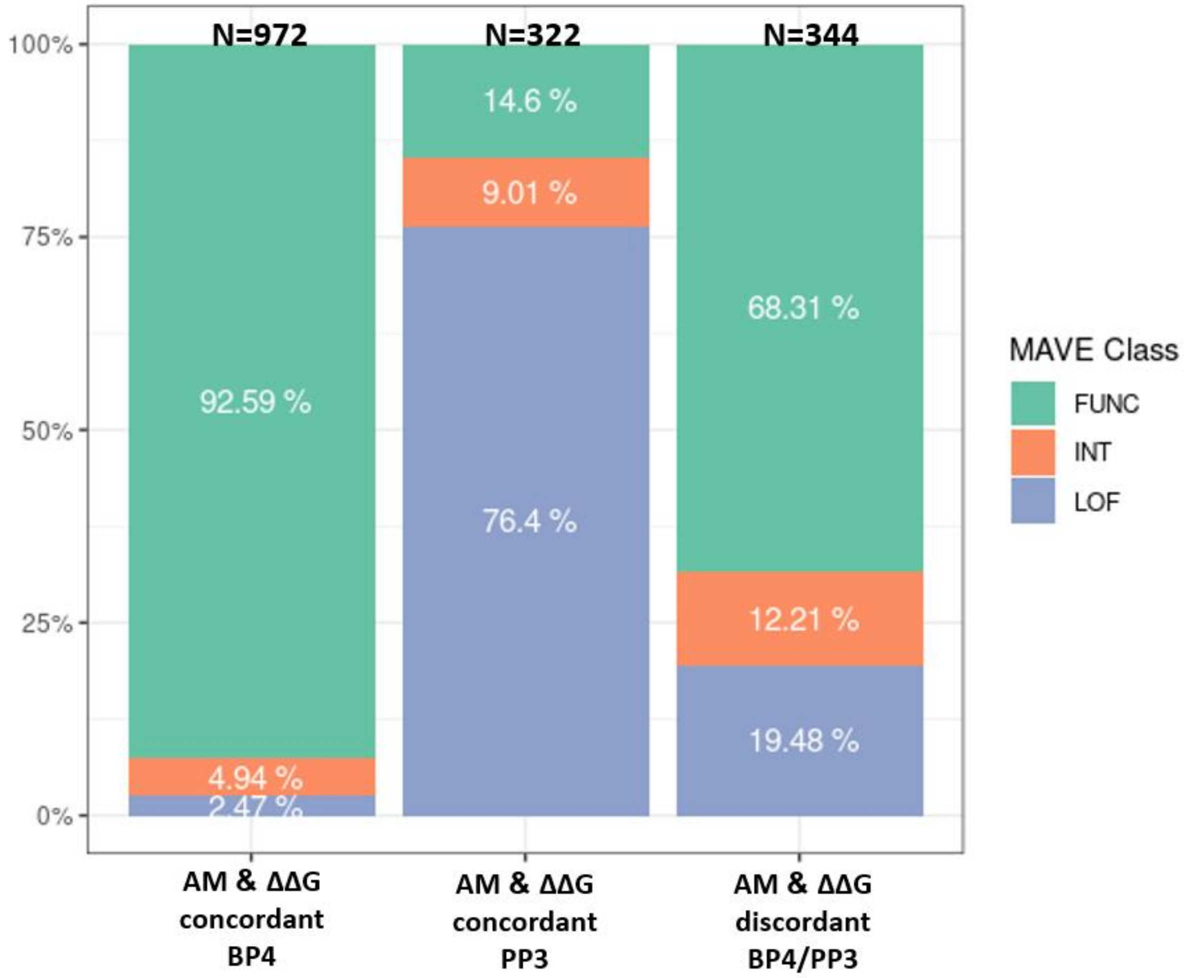


**Figure S13.** The bar plot shows the distribution of MAVE functional classes in the subgroup of variants (N=972) with concordant AlphaMissense- and $\Delta\Delta G^{AF}$-based BP4 evidence (left), in the subgroup of variants (N=322) with concordant AlphaMissense- and $\Delta\Delta G^{AF}$-based PP3 evidence (center), and in the subgroup of variants (N=344) with discordant AlphaMissense- and $\Delta\Delta G^{AF}$-based computational evidence. Interestingly, the higher proportion of INT variants (12.2%) is observed in the subgroup of variants with AM and $\Delta\Delta G^{AF}$ discordance. For the purpose of this analysis, concordant AM- and $\Delta\Delta G^{AF}$-based BP4 evidence if AM<0.75 and $\Delta\Delta G^{AF}$ <2.5, concordant AM- and $\Delta\Delta G^{AF}$-based PP3 evidence if AM ≥0.75 and $\Delta\Delta G^{AF}$ ≥2.5), and discordant AM- and $\Delta\Delta G^{AF}$ if AM<0.75 and $\Delta\Delta G^{AF}$ ≥2.5 (or AM ≥0.75 and $\Delta\Delta G^{AF}$ <2.5).

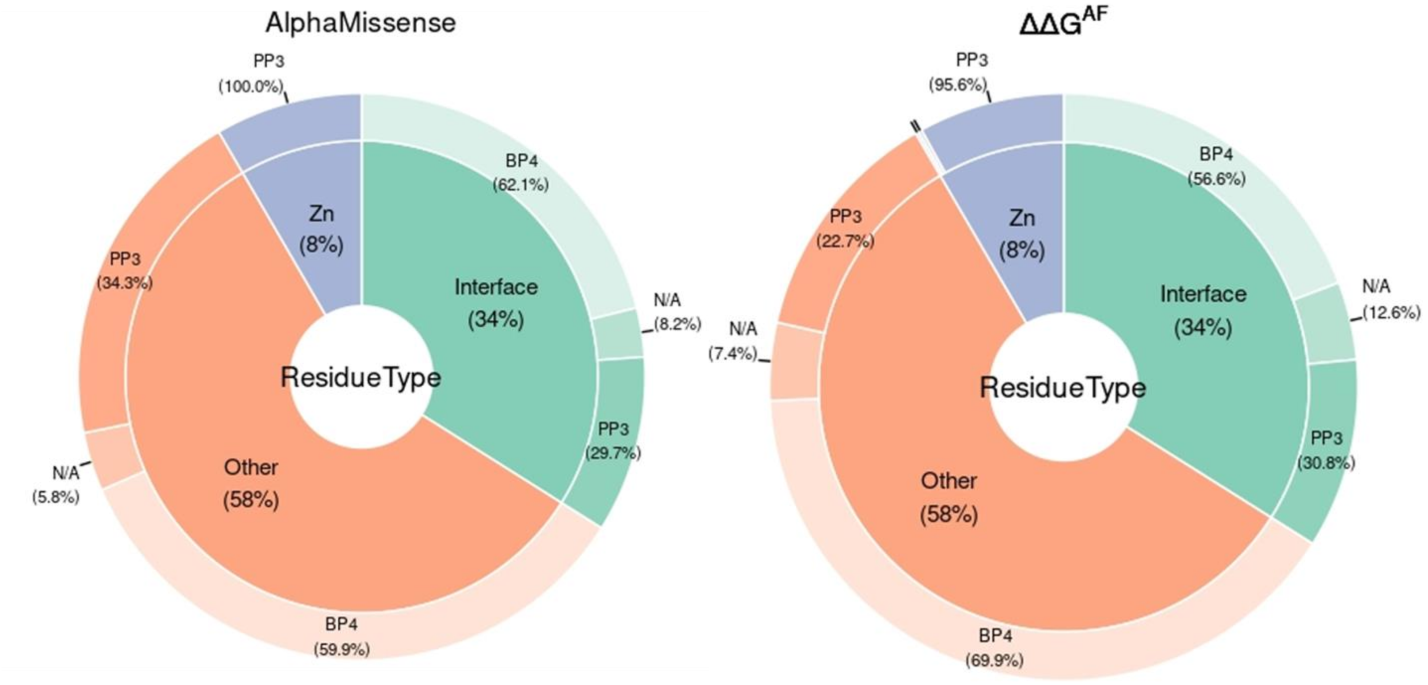


**Figure S14.** The inner circle of the donut plots show BRCA1 RING residues manually stratified into three residue subtypes: Zn-interacting, BARD1 interface, and others. The external circle (left) shows the proportion of AlphaMissense-based PP3, BP4, and no computational code applicable (N/A) missense variants *per* subtype. The external circle (right) shows the proportion of $\Delta\Delta G^{AF}$-based PP3, BP4, and no computational code applicable (N/A) missense variants *per* class. Note that no major differences between AlphaMissense and $\Delta\Delta G^{AF}$ are observed in the subtype of variants targeting Zn-interacting residues (essentially, all variants score PP3), or BARD1 interacting residues. By contrast, AlphaMissense provides a higher proportion of PP3 variants (34% vs. 23%) in the subtype of variants targeting "other residues". The data suggest that most BRCA1 missense variants targeting Zn- or BARD1-interacting residues are pathogenic via impact on protein stability, while a significant proportion of missense variants targeting "other residues" might be pathogenic via mechanisms other than destabilization.

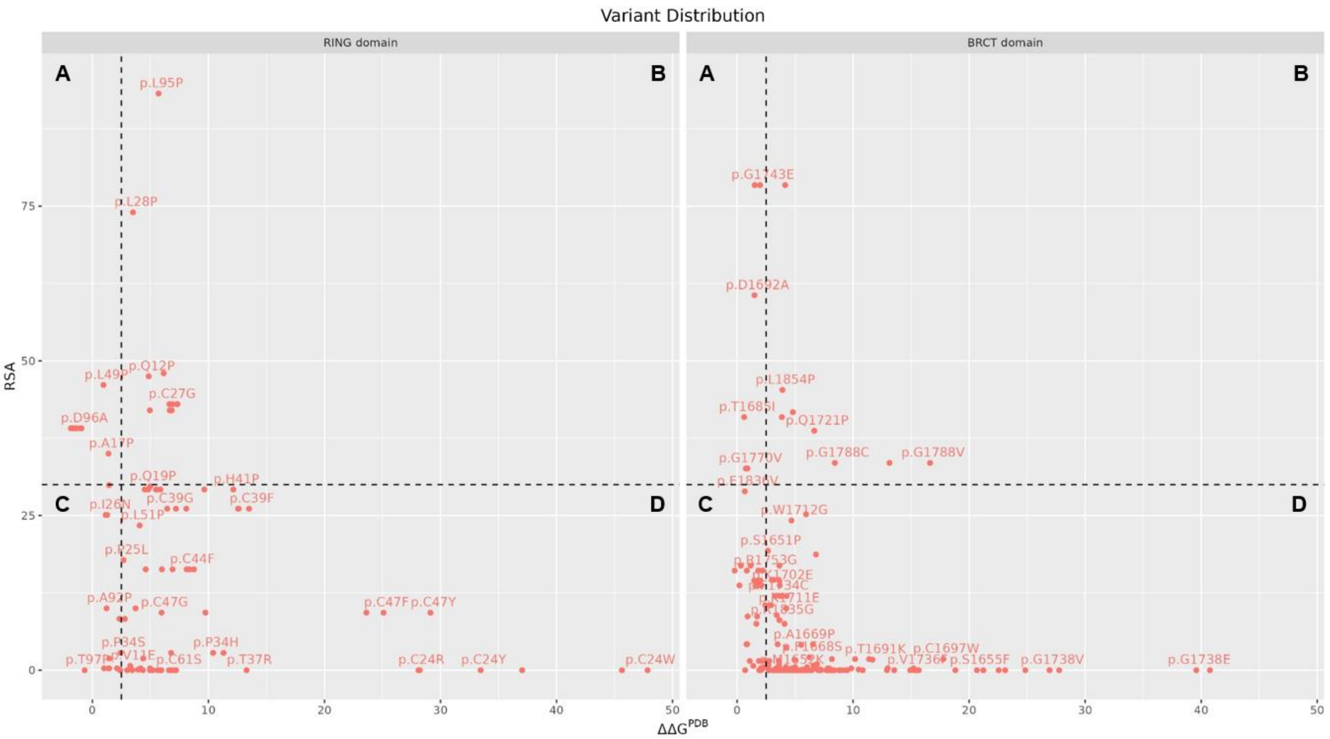


**Figure S15.** All displayed variants score LOF (MAVE) and PP3 (AlphaMissense). For the purpose of this example, we will refer to pathogenic variants. The left (RING domain) and right (BRCT domain) plots show BRCA1 missense variants (91 RING and 188 BRCT) distributed by RSA and $\Delta\Delta G^{PDB}$ (FoldX5.0 prediction). Dashed lines set at 30% RSA and ΔΔG +2.5 Kcal/mol define four plot sectors named A (non-destabilizing pathogenic variants targeting exposed residues), B (destabilizing pathogenic variants targeting exposed residues), C (non-destabilizing pathogenic variants targeting buried residues), and D (destabilizing pathogenic variants targeting buried residues). Most RING (64%) and BRCT (77%) pathogenic variants cluster in D, supporting that reduced thermodynamic stability is a major driver of pathogenicity for BRCA1 missense variants at the RING and BRCT domains. As expected, non-destabilizing pathogenic variants tend to target exposed residues (45% of A+B variants vs. 16% of C+D variants). A+C variants target residues that probably have relevant functions other than contributing to stabilization of the folded domain. For instance, A variants (partially exposed) might target surface residues involved in interactions with relevant protein partners, and C variants (buried) might be relevant for intermediate steps of the folding process.

## Supplemental Tables

**Table S1.** Structure-informed scores for 1638 *BRCA1* missense variants with functional data (see accompanying Excel file)

| Location of target residues | MAVE functional class | N | MAVE functional score | RSA (%) | ΔΔG[PDB] (kcal/mol) | ΔΔG[AF] (kcal/mol) | AM | BD |
|---|---|---|---|---|---|---|---|---|
| Domain stratification | | | | | | | | |
| **RING** | LOF | 103 | -2.29 | 18.27 | 7.09 | 8.51 | 0.93 | 0.43 |
| | INT | 49 | -1.01 | 21.16 | 3.00 | 3.54 | 0.67 | 0.25 |
| | FUNC | 384 | -0.16 | 41.65 | 0.64 | 0.85 | 0.44 | 0.12 |
| | | | | | | | | |
| **BRCT** | LOF | 234 | -2.06 | 9.42 | 5.77 | 5.69 | 0.84 | 0.34 |
| | INT | 70 | -1.02 | 24.41 | 3.16 | 3.31 | 0.55 | 0.20 |
| | FUNC | 798 | -0.07 | 41.81 | 1.08 | 1.13 | 0.28 | 0.06 |
| RSA stratification | | | | | | | | |
| **Buried** (RSA<30%) | LOF | 286 | -2.11 | 5.53 | 6.61 | 6.74 | 0.88 | 0.37 |
| | INT | 87 | -1.03 | 6.83 | 3.99 | 4.22 | 0.69 | 0.26 |
| | FUNC | 506 | -0.15 | 9.81 | 1.54 | 1.70 | 0.44 | 0.13 |
| | | | | | | | | |
| **Partially Buried** (30%≤RSA≤60%) | LOF | 40 | -2.17 | 40.56 | 3.93 | 6.09 | 0.81 | 0.39 |
| | INT | 14 | -0.98 | 47.76 | 0.82 | 1.72 | 0.44 | 0.08 |
| | FUNC | 277 | -0.09 | 45.30 | 0.70 | 0.79 | 0.27 | 0.07 |
| | | | | | | | | |
| **Exposed** (RSA>60%) | LOF | 11 | -1.97 | 80.20 | 2.93 | 3.36 | 0.66 | 0.28 |
| | INT | 18 | -0.96 | 82.39 | 0.56 | 0.77 | 0.30 | 0.11 |
| | FUNC | 399 | -0.05 | 79.81 | 0.35 | 0.38 | 0.23 | 0.03 |

**Table S2** displays relevant MAVE dataset average scores stratified by functional domain (RING vs. BRCT), or residue solvent accessibility (**RSA**). MAVE functional categories and functional scores as reported in the original publication (see methods). **$\Delta\Delta G^{PDB}$** (FoldX5.0 ΔΔG predictions using experimental PDBs as input templates). **$\Delta\Delta G^{AF}$** (FoldX5.0 ΔΔG predictions using AlphaFold2 models as input templates). **AM** (AlphaMissense pathogenicity scores). **BD** (BayesDel scores).

| Tool | BRCA1 domain | Benignity evidence (BP4) | | Bioinformatic code not applicable (%) | Pathogenicity evidence (PP3) | |
|---|---|---|---|---|---|---|
| | | threshold | Evidence strength Log2 LR (95% CI) | | threshold | strength Log2 LR (95% CI) |
| **AM** | RING | <=0.65 | **-3.599** (-4.723 to -2.476) | 6% | >=0.75 | **+2.043** (+1.760 to +2.328) |
| | BRCT | | **-2.741** (-3.216 to -2.267) | 5% | | **+3.507** (+3.135 to +3.878) |
| | | | | | | |
| **$\Delta\Delta G^{AF}$** | RING | <=1.5 | **-3.312** (-4.274 to -2.348) | 8% | >=2.50 | **+2.778** (+2.393 to +3.163) |
| | BRCT | | **-2.789** (-3.353 to -2.225) | 14% | | **+2.187** (+1.947 to +2.428) |
| | | | | | | |
| **BD** | RING | <=0.15 | **-3.038** (-4.004 to -2.018) | 17% | >=0.28 | **+2.397** (+2.053 to +2.741) |
| | BRCT | | **-2.888** (-3.438 to -2.339) | 13% | | **+2.802** (+2.500 to +3.104) |

**Table S3.** The table shows the performance of **AM** (AlphaMissense), **$\Delta\Delta G^{AF}$** (FoldX5.0 predictions), and **BD** (BayesDel)-based PP3/BP4 computational evidence stratified by BRCA1 domain (RING vs. BRCT). We use the MAVE dataset (INT variants filtered-out) as proxy for pathogenicity and benignity. For **AM** and **$\Delta\Delta G^{AF}$,** we use optimal cut-off thresholds (see Table 1 and Figure 2). For **BD**, we used cut-off thresholds defined by the ClinGen ENIGMA *BRCA1* and *BRCA2* variant curation expert panel. Regardless of the BRCA1 functional domain, the computational tools provide benignity and pathogenicity evidence strengths in the moderate to strong range. AM outperforms other computational tools in providing similar evidence strengths with a lower proportion of variants in the BP4/PP3 not applicable range.

| | threshold | BRCA1 Domain | unique missense variants | BC | controls | OR (95% CI) | *p* |
|---|---|---|---|---|---|---|---|
| **AM** | >=0.75 | RING | 12 | 61 | 14 | 3.9 (2.2-7.0) | $5.5x10^{-6}$ |
| | | BRCT | 21 | 33 | 4 | 7.4 (2.4-20.9) | $1.7x10^{-4}$ |
| | | | | | | | |
| | <=0.65 | RING | 24 | 33 | 23 | 1.3 (0.8-2.2) | n.s. |
| | | BRCT | 58 | 113 | 78 | 1.3 (1.0-1.7) | n.s. |
| | | | | | | | |
| **$\Delta\Delta G^{AF}$** | >=+2.5 | RING | 10 | 62 | 11 | 5.1 (2.7-9.6) | $1.1x10^{-6}$ |
| | | BRCT | 22 | 35 | 9 | 3.5 (1.8-7.3) | $8.7x10^{-4}$ |
| | | | | | | | |
| | <=+1.5 | RING | 20 | 26 | 18 | 1.3 (0.7-2.4) | n.s. |
| | | BRCT | 47 | 96 | 73 | 1.2 (0.9-1.6) | n.s. |
| | | | | | | | |
| **$\Delta\Delta G^{PDB}$** | >=+2.5 | RING | 10 | 59 | 13 | 4.1 (2.2-7.4) | $6.1x10^{-6}$ |
| | | BRCT | 25 | 62 | 17 | 3.3 (1.9-5.6) | $1.8x10^{-5}$ |
| | | | | | | | |
| | <=+1.5 | RING | 24 | 32 | 23 | 1.3 (0.7-2.1) | n.s. |
| | | BRCT | 44 | 93 | 67 | 1.2 (0.9-1.1) | n.s. |
| | | | | | | | |
| **BD** | >=0.28 | RING | 7 | 58 | 9 | 5.8 (2.9-11.7) | $1.4x10^{-6}$ |
| | | BRCT | 26 | 62 | 21 | 2.6 (1.6-4.3) | $1.3x10^{-4}$ |
| | | | | | | | |
| | <=0.15 | RING | 21 | 16 | 17 | 0.8 (0.4-1.7) | n.s. |
| | | BRCT | 49 | 72 | 58 | 1.1 (0.8-1.6) | n.s. |

**Table S4. BRIDGES-based Breast Cancer risk estimates stratified by AM, $\Delta\Delta G^{AF}$, and BD scoring.** The BRIDGES-based burden analysis (population-based only, 53,572 **BC** cases and 48,048 matched **controls**, see methods for details) demonstrates that, on average, RING or BRCT missense variants scoring AM≥0.75, $\Delta\Delta G^{AF}$≥2.5, $\Delta\Delta G^{PDB}$≥2.5, or BD≥0.28 are risk associated, supporting PP3 evidence. Similarly, the study supports BP4 for RING or BRCT missense variants scoring AM ≤0.65, $\Delta\Delta G^{AF}$≤1.5, $\Delta\Delta G^{PDB}$≤1.5, or BD ≤0.15. AM (AlphaMissense). (n.s.) no statistically significant

| Tool | Benignity evidence (**BP4**) Log2 LR (95% CI) | RSA | Pathogenicity evidence (**PP3**) Log2 LR (95% CI) |
|---|---|---|---|
| AM | **-3.11** (-3.66 to-2.55) | <=30% | **+2.10** (+1.85 to +2.36) |
| | **-2.00** (-2.84 to-1.17) | 30-60% | **+3.89** (+3.11 to +4.67) |
| | -1.32 (-2.45 to -0.19)* | >60% | **+3.07** (+2.12 to +4.01) |
| | | | |
| $\Delta\Delta G^{AF}$ | **-2.62** (-3.18 to -2.05) | <=30% | **+1.52** (+1.31 to +1.74) |
| | **-3.02** (-4.36 to -1.68) | 30-60% | **+3.35** (+2.76 to +3.95) |
| | -1.69 (-3.09 to -0.30)* | >60% | **+3.67** (+2.77 to +4.56) |
| | | | |
| $\Delta\Delta G^{PDB}$ | **-2.24** (-2.73 to -1.76) | <=30% | **+1.57** (+1.34 to +1.80) |
| | **-2.11** (-2.50 to -1.73) | 30-60% | **+2.51** (+1.87 to +3.95) |
| | -1.73 (-3.12 to -0.34)* | >60% | **+3.41** (+2.27 to +3.14) |
| | | | |
| BD | **-2.83** (-3.39 to -2.27) | <=30% | **+1.93** (+1.67 to +2.18) |
| | **-2.61** (-3.80 to -1.43) | 30-60% | **+3.16** (+2.56 to +3.76) |
| | -1.63 (-3.02 to -0.24)* | >60% | **+3.52** (+2.56 to +4.52) |

**Table S5.** The table shows the performance of **AM** (AlphaMissense)-, $\Delta\Delta \mathbf{G}^{\mathbf{AF}}$ (FoldX5.0 predictions)-, $\Delta\Delta \mathbf{G}^{\mathbf{PDB}}$ (FoldX5.0 predictions)-, and **BD** (BayesDel)-based PP3/BP4 computational evidence stratified by RSA. We use the MAVE dataset (INT variants filtered-out) as proxy for pathogenicity and benignity. For **AM**, $\Delta\Delta \mathbf{G}^{\mathbf{AF}}$**,** and $\Delta\Delta \mathbf{G}^{\mathbf{PDB}}$ we use optimal cut-off thresholds (see Table 1 and Figure 2). For BD, we use cut-off thresholds defined by the ClinGen ENIGMA BRCA1 and BRCA2 variant curation expert panel. Note that no bioinformatic tool provides statistically significant benignity evidence of supporting strength for missense variants targeting exposed (RSA>60%) residues (in all cases the Log2 LR 95% CI overlaps -1).

| | RSA | Benignity evidence (**BP4**) Log2 LR (95% CI) | Tool | Pathogenicity evidence (**PP3**) Log2 LR (95% CI) |
|---|---|---|---|---|
| RING | ≤60% (N=354) | **-3.79** (-5.03 to -2.55) | AM | **+1.99** (+1.65 to +2.31) |
| | | **-3.27** (-4.31 to -2.23) | $\Delta\Delta G^{AF}$ | **+2.28** (+1.90 to +2.66) |
| | | **-3.10** (-4.15 to -2.06) | BD | **+2.23** (+1.84 to +2.62) |
| | | | | |
| | >60% (N=134) | -1.35 (-3.76 to +1.05)* | AM | +1.52 (+0.12 to +2.92)** |
| | | -1.73 (-4.12 to +0.65)* | $\Delta\Delta G^{AF}$ | **+4.63** (+2.58 to +6.71) |
| | | -1.30 (-3.70 to +1.09)* | BD | +2.22 (+0.75 to +3.68)** |
| | | | | |
| BRCT | ≤60% (N=755) | **-2.75** (-3.25 to -2.24) | AM | **+2.93** (+2.57 to +3.30) |
| | | **-2.67** (-3.27 to -2.08) | $\Delta\Delta G^{AF}$ | **+1.80** (+1.55 to +2.04) |
| | | **-2.81** (-3.39 to -2.23) | BD | **+2.28** (+1.98 to +2.58) |
| | | | | |
| | >60% (N=227) | -1.49 (-2.80 to -0.18)* | AM | **+8.16** (+4.05 to +12.25) |
| | | -1.76 (-3.49 to -0.03)* | $\Delta\Delta G^{AF}$ | **+3.31** (+2.28 to +4.32) |
| | | -1.86 (-3.59 to -0.13)* | BD | **+5.49** (+3.58 to +7.39) |

**Table S6.** The table shows the performance of **AM** (AlphaMissense), $\Delta\Delta$**G**$^{AF}$ (FoldX5.0 predictions), and **BD** (BayesDel)-based PP3/BP4 computational evidence stratified by BRCA1 functional domain (RING vs. BRCT) and RSA (≤60% vs. >60%). We use the MAVE dataset (INT variants filtered-out) as proxy for pathogenicity and benignity. (N) Sample size (number of MAVE variants in each category) is indicated. For **AM** and $\Delta\Delta$**G**$^{AF}$**,** we use optimal cut-off thresholds (see Table 1 and Figure 2). For **BD**, we use cut-off thresholds defined by the ClinGen ENIGMA BRCA1 and BRCA2 variant curation expert panel. (*) The bioinformatics tool does not provide statistically significant benignity evidence of supporting strength for missense variants targeting very exposed (RSA>60%) residues (Log2 LR 95% CI overlaps -1). (**) The bioinformatics tool do not provides statistically significant pathogenicity evidence of supporting strength for missense variants targeting exposed (RSA>60%) residues (Log2 LR 95% CI overlaps +1)

## Supplemental Methods

*Alamut Visual Plus*

We used Alamut Visual Plus version v1.6.1 (© 2022 SOPHiA GENETICS) to generate SpliceAI and VEP input vcf files.

*SpliceAI-based splicing predictions*

SpliceAI Δ scores [donor loss (DL), acceptor loss (AL), acceptor gain (AG), and donor gain (DG)] were calculated locally using the following parameters: genome version hg38, score type raw, and max distance ±4999. Variants were annotated as bonafide missense (i.e. spliceogenicity discarded) only if none of the four Δ scores was above the 0.20 high recall threshold.

*MAVE dataset*

We worked with a *BRCA1* MAVE experiment reporting RNA and functional scores for 2086 genetic variants annotated as missense (5); 651 target residues mapping to the RING domain (residues 1-101), and 1318 target residues mapping to the BRCT domain (residues 1649-1855). We calculated SpliceAI Δ scores (6) for all 2086 variants. We filtered out variants targeting the initiation codon, and variants predicted spliceogenic (MAVE RNA scores ≤-3 and/or SpliceAI Δ score ≥0.2) to generate a cohort of bonafide missense variants that included mostly singletons, but some redundant doubletons too (two different single nucleotide variants coding the same amino-acid change). For each doubleton with concordant functional scores, we filtered out the variant with the lowest RNA score. For doubletons with discordant functional scores, we filtered-out both variants. We ended up with a cohort of 1638 bonafide missense variants.

*AlphaFold2 models*

Models of the BRCA1 RING and BRCT domains were generated using protein prediction software AlphaFold2. We modeled the RING domain as a BRCA1/BARD1 RING-domain heterodimer, and the BRCT domain as a monomer. In brief, we generate AlphaFold2 models on LatchBio (https://console.latch.bio/) with default parameters. The pipeline generates 10 structures (five relaxed and five unrelaxed) ranked by average pLDDT. As output for $\Delta\Delta G$ predictions, we used the best ranked (highest average pLDDT) relaxed model. Metal3D tool (https://colab.research.google.com/github/lcbc-epfl/metal-site-prediction/blob/main/Metal3D/ColabMetal.ipynb#scrollTo=IsKnyVvLXcj1) was used with default parameters to introduce Zn atoms in the correct conformational space of the BRCA1/BARD1 RING-domain heterodimer model(7). Afterwards, intramolecular distance and correct coordination bonding with Cys and His residues was evaluated using Pymol and Protein-Ligand Interaction Profiler Web tool (https://plip-tool.biotec.tu-dresden.de/plip-web/plip/index)(8).

*ΔΔG predictions*

To compute $\Delta\Delta G^{PDB}$ at the RING domain, we used the only available structure: a NMR solution structure (PDB 1JM7) of the human BRCA1/BARD1 RING-domain heterodimer (9). To compute $\Delta\Delta G^{PDB}$ at the BRCT domain, we selected a high-resolution (1.85 Å) X-Ray diffraction structure (PDB 1T15) of the human BRCA1 BRCT Domains in Complex with the Phosphorylated Interacting Region from Bach1 Helicase (10). To compute $\Delta\Delta G^{AF}$, we generate AlphaFold2 models for a BRCA1/BARD1 RING-domain heterodimer and for a BRCA1 BRCT monomer.

We run FoldX5.0 locally. First, we perform a preprocessing step of the input PDB file with the RepairPDB command. Later, we run the BuildModel command in triplicate to compute an average ΔΔG per missense variant.

Command line executed:

*foldx –command RepairPDB –pdb protein_structure.pdb*

*foldX –command BuildModel –pdb protein_structure_Repair.pdb –mutant-file individual_list.txt –numberOfRuns 3*

Protein_structure.pdb corresponds to experimental PDBs 1JM7and 1T15, and AF models for ΔΔG PDB and ΔΔG AF, respectively.

In the subgroup of variants targeting the RING domain, we perform the following additional analyses: (i) we label interface residues using AnalyseComplex, (ii) we label Zn-interacting residues manually (9), and (iii) we compute ΔΔG for protein-protein interaction (ΔΔGint) using the PSSM command.

*Command line executed:*

*foldX –command AnalyseComplex –pdb protein_structure_Repair.pdb*

*foldX –command PSSM –pdb protein_structure_Repair.pdb*

In addition, we predicted ΔΔG for the entire MAVE dataset by running SDM (11), INPS-3D (12), POPMUSIC (13), Dynamut2 (14), MAESTRO (15), and CUPSAT (16) in their corresponding web-servers (default parameters). For SDM, DYNAMUT2, INPS3D and CUPSAT we reversed the output sign, so that positive ΔΔG changes denote reduced stability. Collectively, we will refer to web-based ΔΔGs.

<u>Case-control Validation Dataset</u>

BRIDGES (17) variant-level counts from breast cancer cases (population-based only, N=53,572) and matched controls (N=48,048) were retrieved from <u>bcac.ccge.medschl.cam.ac.uk/bcacdata/</u> (last accessed 22/10/2023). In total, 765 missense variants annotated as missense were obtained. Of these, 135 variants targeted the RING or BRCT domains. After filtering-out likely

spliceogenic (SpliceAI Δ score ≥0.2) variants, and one common variant targeting the BRCT domain [c.4956G>A p.(Met1652Ile), MAF=0.014], we end-up with a cohort of 122 bona-fide missense variants with case-control counts (**BRIDGES dataset**). Statistical analyses were performed in R. Odds ratio (OR) of cases and controls for these genes was calculated using selected ΔΔG cutoffs and methodology based on Altman, 1991(18).

Stratified LR analysis

To assign evidence weights based on the combination of RSA, AlphaMissense and ΔΔG we used the approaches summarized in Figures S9-S11.

In the first approach (**Figure S9**), we simply annotated all variants under assessment as concordant benign (if AlphaMissense ≤0.65 and $\Delta\Delta G^{AF}$ ≤Δ1.5), concordant pathogenic (if AlphaMissense ≥0.75 and $\Delta\Delta G^{AF}$ ≥Δ2.5), and others (variants with any other combination of AlphaMissense and $\Delta\Delta G^{AF}$ scores). Later, we defined a new *concordant score* coding all concordant benign variants with "-1", all concordant pathogenic variants with "+1", and all other variants with "0".Finally, we obtained Log2 LRs by running the new concordant score in an on-line LR calculator (gwiggins.shinyapps.io/lr_shiny), with cut-off thresholds set at -0.5 and +0.5.

In the second approach (**Figure S10**), we first stratified variants according to RSA into buried/partially buried variants (RSA≤60%) and exposed variants (RSA>60%), and later we analyze the *concordant score* as explained above.

In the third approach (**Figure S11**), we first stratified by RSA. Later, we used the on-line LR calculator to produce AlphaMissense-based Log2 LRs in the buried/partially-buried and exposed sub-groups. Next, we used the on-line LR calculator to produce ΔΔG-based Log2 LRs in four variant sub-groups (RSA≤60% + AlphaMissense ≤0.65, RSA≤60% + AlphaMissense ≥0.75, RS>60% + AlphaMissense ≤0.65, and RSA>60% + AlphaMissense ≥0.75). Finally, we added ΔΔG-based Log2 LR values to the corresponding AlphaMissense-based Log2 LR value to obtain combined Log2 LR values.

**Supplemental Bibliography**


1. Deng CX, Brodie SG. Roles of BRCA1 and its interacting proteins. Bioessays. 2000 Aug;22(8):728–37.

2. Christou CM, Kyriacou K. BRCA1 and Its Network of Interacting Partners. Biology (Basel). 2013 Jan 2;2(1):40–63.

3. Epasto LM, Pötzl C, Peterlik H, Khalil M, Saint-Pierre C, Gasparutto D, et al. NMR-identification of the interaction between BRCA1 and the intrinsically disordered monomer of the Myc-associated factor X. Protein Sci. 2024 Jan;33(1):e4849.

4. Wang Q, Zhang H, Kajino K, Greene MI. BRCA1 binds c-Myc and inhibits its transcriptional and transforming activity in cells. Oncogene. 1998 Oct 15;17(15):1939–48.

5. Findlay GM, Daza RM, Martin B, Zhang MD, Leith AP, Gasperini M, et al. Accurate classification of BRCA1 variants with saturation genome editing. Nature. 2018 Oct;562(7726):217–22.

6. Jaganathan K, Kyriazopoulou Panagiotopoulou S, McRae JF, Darbandi SF, Knowles D, Li YI, et al. Predicting Splicing from Primary Sequence with Deep Learning. Cell. 2019 Jan 24;176(3):535-548.e24.

7. Dürr SL, Levy A, Rothlisberger U. Metal3D: a general deep learning framework for accurate metal ion location prediction in proteins. Nat Commun. 2023 May 11;14(1):2713.

8. Adasme MF, Linnemann KL, Bolz SN, Kaiser F, Salentin S, Haupt VJ, et al. PLIP 2021: expanding the scope of the protein-ligand interaction profiler to DNA and RNA. Nucleic Acids Res. 2021 Jul 2;49(W1):W530–4.

9. Brzovic PS, Rajagopal P, Hoyt DW, King MC, Klevit RE. Structure of a BRCA1-BARD1 heterodimeric RING-RING complex. Nat Struct Biol. 2001 Oct;8(10):833–7.

10. Clapperton JA, Manke IA, Lowery DM, Ho T, Haire LF, Yaffe MB, et al. Structure and mechanism of BRCA1 BRCT domain recognition of phosphorylated BACH1 with implications for cancer. Nat Struct Mol Biol. 2004 Jun;11(6):512–8.

11. Pandurangan AP, Ochoa-Montaño B, Ascher DB, Blundell TL. SDM: a server for predicting effects of mutations on protein stability. Nucleic Acids Res. 2017 Jul 3;45(W1):W229–35.

12. Savojardo C, Fariselli P, Martelli PL, Casadio R. INPS-MD: a web server to predict stability of protein variants from sequence and structure. Bioinformatics. 2016 Aug 15;32(16):2542–4.

13. Dehouck Y, Kwasigroch JM, Gilis D, Rooman M. PoPMuSiC 2.1: a web server for the estimation of protein stability changes upon mutation and sequence optimality. BMC Bioinformatics. 2011 May 13;12:151.

14. Rodrigues CHM, Pires DEV, Ascher DB. DynaMut2: Assessing changes in stability and flexibility upon single and multiple point missense mutations. Protein Sci. 2021 Jan;30(1):60–9.

15. Laimer J, Hiebl-Flach J, Lengauer D, Lackner P. MAESTROweb: a web server for structure-based protein stability prediction. Bioinformatics. 2016 May 1;32(9):1414–6.

16. Parthiban V, Gromiha MM, Schomburg D. CUPSAT: prediction of protein stability upon point mutations. Nucleic Acids Res. 2006 Jul 1;34(Web Server issue):W239-242.

17. Breast Cancer Association Consortium, Dorling L, Carvalho S, Allen J, González-Neira A, Luccarini C, et al. Breast Cancer Risk Genes - Association Analysis in More than 113,000 Women. N Engl J Med. 2021 Jan 20;

18. Altman DG. Practical Statistics for Medical Research (1st ed.). [Internet]. New York: Chapman and Hall/CRC; 1990. 624 p. Available from: https://doi.org/10.1201/9780429258589
